# Impact of Stress on Adrenal and Neuroendocrine Responses, Body Composition, and Physical Performance Amongst Women in Demanding Tactical Occupations: A Scoping Review

**DOI:** 10.3390/metabo15080506

**Published:** 2025-07-29

**Authors:** Tunde K. Szivak, Erica A. Schafer, Hayley V. MacDonald, Catherine Saenz

**Affiliations:** 1 Department of Athletic Training and Exercise Science, School of Health Sciences, Merrimack College, North Andover, MA 01845, USA; szivakt@merrimack.edu; 2 Department of Kinesiology, College of Education, The University of Alabama, Tuscaloosa, AL 35487, USA; easchafer@crimson.ua.edu (E.A.S.); hvmacdonald@ua.edu (H.V.M.); 3 Exercise Science, Kinesiology, Department of Human Sciences, College of Education and Human Ecology, The Ohio State University, Columbus, OH 43210, USA

**Keywords:** women, stress, tactical athletes, body composition, exercise performance, tactical occupations, scoping review

## Abstract

Background/Objectives: This scoping review critically evaluated existing literature and summarized the impact of occupational, physiological, and psychological stressors on adrenal and neuroendocrine responses, body composition, and physical performance amongst women in tactical occupations. Methods: Boolean searches identified potentially qualifying reports involving: (1) adult women (≥19 y) currently employed or completing their training for a tactical profession; (2) ≥1 marker of “stress”; and (3) ≥1 adrenal, neuroendocrine, body composition, or fitness/performance outcome. Quantitative data (e.g., sample characteristics, outcomes of interest) were extracted and summarized. The completeness of reporting for each study was documented using existing checklists and quantified as: low (<50%), moderate (50–79%), or high (≥80%). Results: 40 studies (*k*) of moderate reporting quality (~64%) were included in the final sample (3693 women); 11 studies (28%) focused on women exclusively, and 16 studies identified sex differences in ≥1 outcome. Most studies involved military trainee populations (80%, *k* = 32). Occupation-related stress tended to negatively impact adrenal, neuroendocrine, body composition, and performance outcomes. Conclusions: This review highlights progress in assessing occupational performance in female tactical personnel exposed to diverse stressors; however, our understanding remains incomplete due to methodological and conceptual limitations in the literature. Holistic research strategies are needed to capture the complexity of performance readiness in women, integrating how stress affects key tactical performance aspects such as muscle physiology, reproductive health, and energy and nutrient balance in realistic operational contexts. Integrating such data is vital for informing policy, improving readiness, and enhancing the health and career longevity of female tactical personnel.

## 1. Introduction

Women now represent approximately 9–20% of the workforce in tactical professions, including military, law enforcement, firefighting, emergency medical services (EMS), and corrections [1,2]. Women’s expanding presence in physically and psychologically demanding tactical occupational roles has prompted a growing need to better understand the unique physiological and performance-related demands placed on women in these environments. In the United States (U.S.), women now serve across all branches of the military, including previously restricted combat arms roles, and are eligible for selection into elite units (e.g., Infantry, Special Forces) [3]. In other tactical sectors such as law enforcement and wildland firefighting, women have served for decades, though their representation and access to specialized teams (e.g., Special Weapons and Tactics unit) remain variable across jurisdictions. Internationally, women serve in both volunteer and conscripted forces, with some integrated into combat arms units [4]. Despite advancements in access and representation, critical gaps persist in understanding the physiological, psychological, and occupational implications of tactical service for women.

**Figure 1:**
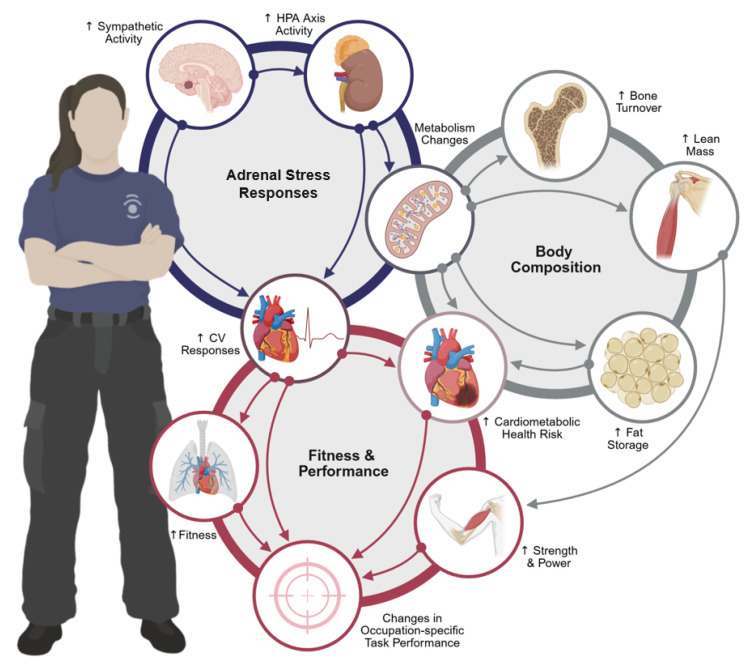
Relationships (identified through arrows) between acute and chronic stressors and their potential impacts on adrenal and neuroendocrine responses, body composition, and physical performance. The cumulative impact of occupational stressors (i.e., allostatic load) and the inter-connected nature of systemic effects can lead to outcomes such as an overdriven adrenal and neuroendocrine system, worsening cardiometabolic health and body composition profiles, and undesirable changes in physical performance. This image was created using BioRender.com (https://BioRender.com), Procreate^®^ (version 5.3.15), and PowerPoint (version 2.98.4).

Tactical professions are characterized by high physiological demands, including heavy load carriage [5,6,7], environmental exposures (e.g., heat, cold, smoke, high altitude) [8,9,10,11], prolonged operational stress [12,13,14], and disrupted sleep and circadian rhythms [14,15]. These are further compounded by psychosocial challenges, including exposure to trauma [14,16], underrepresentation in male-dominated units, and elevated risk for gender-specific stressors such as sexual harassment or assault [17,18,19]. This stress may accumulate over years of service (i.e., chronic) or may result from short-term events (i.e., acute) such as high-intensity training programs or brief, demanding operations (e.g., Basic Combat Training, disaster response scenarios). Tactical training and occupational stress have systemic physiological impacts and may negatively impact readiness and performance. The cumulative impact of occupational stressors (i.e., allostatic load) and the interconnected nature of systemic effects can lead to outcomes such as an overdriven adrenal and neuroendocrine system, worsening cardiometabolic health and body composition profiles, and undesirable changes in physical performance. This combination of outcomes has the potential to negatively influence occupation-specific task performance (Figure 1).

The adrenal stress response (Figure 1) refer to the physiological adaptation relative to imposed stress (i.e., adaptation to imposed demands) [20]. The adaptive response to imposed demands serves specific physiological purposes, for example, mobilization of energy stores, delivery of oxygenated blood to metabolically-active tissues, and activation of contractile tissue (i.e., skeletal muscle). Core components of the physiological stress response are the hypothalamus and adrenal glands. The hypothalamic–pituitary (HPA) axis governs the synthesis and secretion of the stress hormone cortisol from the adrenal cortex, while sympathetic nervous system signaling stimulates the adrenal medulla to release catecholamines into circulation as part of the stress response. The magnitude of response to an imposed stressor is relative to the magnitude and duration of demand, regardless of the nature of the stressor (e.g., environmental exposure, physical exertion, occupational demand). Likewise, combined stressors (e.g., intense training bouts, energy deficit, sleep deprivation, psychological stress) have a cumulative impact on the magnitude of stress response [21,22]. Therefore, it follows that tactical occupational demands place a significant adaptation demand on human physiology.

Previous works have established sex differences in stress reactivity, with women initiating HPA axis activity more rapidly, leading to a heightened stress response [23]. This, coupled with sex differences in neuromuscular performance [24,25] and energy metabolism, likely impacts downstream health and performance outcomes [21], prompting notable consequences in tactical settings and thus, a need for more female tactical athlete-focused research. Current tactical training and nutrition recommendations aimed at supporting physical readiness include some female-specific guidelines but have been primarily established through a male-focused lens [26]. Occupational policies and physical training practices have not always adequately addressed, or fully appreciated, the interconnectedness between stress physiology and female-focused factors such as musculoskeletal health and body composition, reproductive health, and physical performance outcomes; each of which includes considerations that impact physical and occupational readiness.

Although the body of research on women in tactical roles is growing, it remains disproportionately focused on male populations [27]. Women’s needs relative to the tactical profession are not fully understood, and little is known about how women’s unique physiological characteristics influence their responses to stress, injury risk, recovery, and long-term performance in these professions. Key areas directly related to tactical performance, such as adrenal stress response, neuroendocrine adaptation, metabolic and skeletal muscle health, and occupational performance under load or fatigue, remain largely underexamined in female cohorts. This knowledge gap offers an opportunity to explore the relationship between tactical stress and readiness, with the intent of using this newly gained knowledge to build targeted tactical health and performance programs that incorporate women-specific considerations.

Thus, the purpose of this scoping review is to synthesize the current literature on women in tactical occupations, with a focused analysis of three interrelated domains: adrenal stress and neuroendocrine responses to occupational stressors, important metabolic health indicators (e.g., body composition), and physical and occupational performance outcomes across training, field operations, and simulated environments. The objectives of this scoping review are to (1) provide a comprehensive overview of the research examining women’s physiological responses and occupational performance to stressful environments in tactical professions; (2) identify key areas of consensus, as well as critical gaps in the literature that hinder effective policy and program development, and (3) offer evidence-informed recommendations for future research and operational readiness and performance optimization strategies that address the specific needs of women in these demanding roles. As more women enter and advance within tactical professions, it is essential to generate sex-specific data that informs occupational health, readiness standards, and long-term career sustainability. This scoping review aims to serve as a foundation in support of these efforts.

## 2. Materials and Methods

We performed a scoping review in accordance with the *P*referred *R*eporting *I*tems for *S*ystematic Review and *M*eta-*A*nalyses extension for Scoping Reviews (PRISMA-ScR) Statement [28]. Institutional review board approval was not required for this study since it is a review of existing literature and is not considered intervention research involving humans. The protocol for this scoping review was developed and revised by the research team and is available from the corresponding author upon request.

### 2.1. Inclusion Criteria

Studies were eligible for inclusion if they satisfied four pre-established inclusion criteria: (1) involved adult participants (≥19 y) that were currently employed in a tactical profession (e.g., military, police, firefighter, emergency medical services) or completing their training for employment (e.g., recruits or cadets); (2) included all women samples or mixed sample where women are analyzed/reported separately; (3) examined at least one marker of “stress”—broadly (i.e., physiological, neuroendocrine, psychological, or occupational stress or strain) or specifically (i.e., blood biomarker, such as circulating epinephrine) defined; and (4) measured at least one outcome related to body composition (e.g., lean mass, fat free mass, or muscle mass; body fat [BF], fat mass, or adiposity) or physical fitness and performance (e.g., fitness test or occupational-specific task).

### 2.2. Search Strategy

Potential qualifying reports were identified and retrieved from PubMed, EMBASE (via Scopus), and Web of Science using a Boolean search strategy using terms related to “tactical athlete” or “tactical occupation” and outcomes related to stress, body composition, physical fitness, and performance. Searches were not restricted by language, and databases were searched from their inception or earliest coverage date through 26 February 2025. The full search strategy for each electronic database is provided in Table S1 in the Supplementary Materials. Reference lists of included studies, relevant reviews, and meta-analyses were manually searched for additional reports.

**Figure 2:**
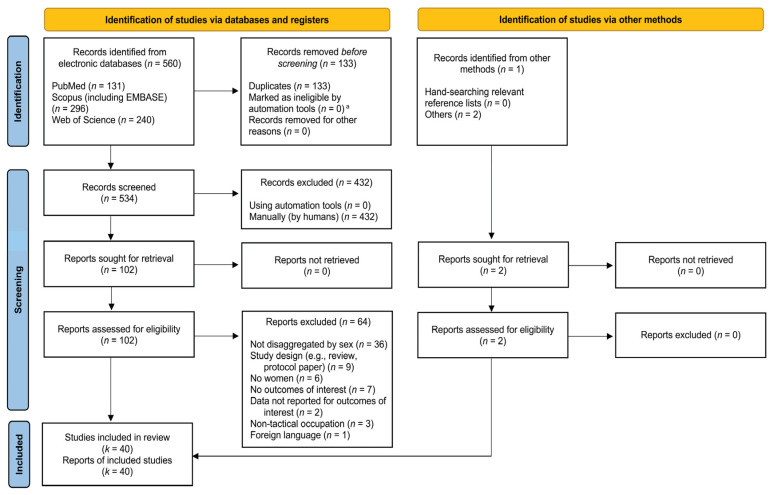
Flow chart detailing the search for potential reports (*n*) and selection process of included studies (*k*). ^a^ Rayyan.ai was used to identify potentially ineligible records during the screening process only. No records were excluded using automation tools, as all were performed by humans (EAS, HVM).

Our electronic and manual search methods yielded 667 potentially qualifying reports. After removing duplicates, 534 records remained and were reviewed for inclusion by two authors (EAS, HVM); 102 reports were retrieved for full review. Figure 2 details the search and selection process undertaken for this review.

### 2.3. Data Extraction

Variables of interest were extracted and summarized by major outcomes of interest: adrenal stress and neuroendocrine responses; metabolic health (e.g., body composition, muscle mass); physical fitness and occupational performance. Coded variables include: (1) characteristics of the study, sample, and intervention/exposure (if applicable); (2) the outcomes analyzed and relevant methodology employed; (3) study data, expressed as mean ± standard deviation (SD) (or other quantitative summary), extracted directly from the study, denoting sex differences (whenever possible); and (4) a brief summary regarding the impact of stress and sex on the outcome of interest. Data extraction was performed by one member of the research team (EAS) and checked by a second member (HVM). All disagreements were resolved by discussion.

### 2.4. Critical Appraisal of Individual Sources of Evidence

A critical appraisal of the studies included in our scoping review was performed to gauge the completeness of reporting (i.e., reporting quality) using the STROBE Checklist for observational, cross-sectional, and cohort studies (95% of our sample) [29] and the CONSORT Checklist for studies with an interventional design (5% of our sample) [30]. Of note, the sole intervention study [31] was not a randomized trial; therefore, the CONSORT checklist was modified, such that questions pertaining to randomization were omitted. For each checklist, we generated an overall score to reflect reporting completeness, which was gauged as a percentage of items satisfied. We used the following thresholds, based on the distribution of scores in our sample using quartiles, to interpret reporting quality scores: low (<50% of items satisfied, quartile 1), moderate (50–79% of items satisfied, quartiles 2 and 3), or high (≥80% of items satisfied, quartile 4), respectively. Reporting quality scores for each study are provided within the summary tables for each outcome of interest.

## 3. Results

Of the 534 potentially eligible reports identified (after removing duplicates), 104 were obtained for full-text review (102 from electronic searches; 2 from other methods) and independently evaluated by two members of the research team (EAS, HVM), revealing a final sample of 40 studies (*k*) that satisfied inclusion criteria [22,24,31,32,33,34,35,36,37,38,39,40,41,42,43,44,45,46,47,48,49,50,51,52,53,54,55,56,57,58,59,60,61,62,63,64,65,66,67,68,69]. Figure 2 shows the systematic search for potential reports and the selection process of included studies. More than half of potentially eligible reports (56%; *k =* 36) were deemed ineligible during full-text review because they failed to disaggregate data by sex. The 64 studies that were excluded following full-text review are summarized in Table S2 as part of the Supplementary Materials.

The majority (80%) of studies in this scoping review focused on military populations; only eight studies (20% of our sample) included police, firefighting, or EMS professions [35,40,41,50]. Twenty-four studies examined two or more outcomes of interest [24,34,36,37,38,40,41]; of these, only five studies evaluated all three of our major outcomes of interest [22,33,55,56,68]. Eleven studies (28%) focused on women exclusively; of the 29 studies with mixed samples, only half (55%; *k* = 16) evaluated sex differences, and 14 studies found significant differences in one or more outcomes [24,31,34,35,37,38,41,43,49,54,56,57,59,68,69].

Overall, our review summarizes data from 3693 women, most of whom were recruits or cadets (87%). Studies that included servicewomen (*k* = 12), reported ~8 years (y) of service, although this ranged from 3 to 14 y. See Table 1 for a summary of included studies in the total sample and by tactical occupation. Table 2, Table 3 and Table 4 summarize the coded dimensions for each of the studies included in this review, grouped by the major outcomes of interest.

### 3.1. Critical Appraisal of Individual Sources of Evidence

In the total sample, included studies achieved “moderate” reporting quality (~63%) despite widely varying scores (27–88%). Four studies achieved “low” reporting quality (<50% of checklist items were completely reported; mean score = 44.3%) [36,39,43,67], and only three studies achieved “high” reporting quality (≥80% of checklist items were completely reported; mean score = 83.3%) [34,45,59].

Studies were most likely to completely report (≥80%) on items relating to adequately describing the study and key findings in the abstract (item 1b), the background and rationale (item 2), the variables to be measured (including the measures and tools) and how they will be handled in analyses (items 7, 8 and 11), reporting the characteristics of their sample and outcomes of interest (items 14a, 15, and 16a), and finally, summarizing and interpreting their findings in the discussion section (items 18 and 20).

Studies were least likely to completely report on items relating to how their sample size was determined (item 10), considering and describing any sensitivity analyses (item 12e), and the use of a flow diagram to document participant flow and missing data (item 13c). A summary of these individual reporting items (expressed as a percentage of checklist items that were completely reported) is provided in Table S3 as part of the Supplementary Materials.

### 3.2. Adrenal Stress and Neuroendocrine Responses

Nineteen studies (970 women; [Mean ± SD] age = 27 ± 8 y; body mass index [BMI] = 25.3 ± 3.0 kg/m^2^; BF = 29.9% ± 4.9%) evaluated sympathoadrenal responses to occupational stress in tactical populations, the majority of which were conducted in military settings (84%; 16 studies). Only three studies focused on non-military personnel: two in police officers (105 women) [65,67] and one in EMS (28 women) [48]. Most military studies assessed women undergoing basic training or officer candidate school (63%), with only a few involving active-duty personnel (e.g., McGraw et al., 2013 [31]; Andrews et al., 2010 [33]). More than half of the included studies involved mixed samples of women and men, with only 37% (7 studies) focusing exclusively on women. See Table 2 for the individual summaries of the coded dimensions for each study.

One-fourth (26%) of the included studies used a cross-sectional design [33,43,48,65,67], and only one study [31] used an acute pre–post intervention design; the remaining studies were prospective cohort studies. Studies that examined adrenal stress and neuroendocrine responses achieved “moderate” reporting quality (63.1% ± 13.1%) despite widely varying scores (347–88%), and only one study completely reported on ≥80% of checklist items (89.4%) [59].

Cortisol was the most assessed biomarker of adrenal stress, measured either via saliva, blood plasma, or hair. Six studies evaluated salivary cortisol, ten included plasma or serum cortisol, and three incorporated hair cortisol analysis. Notably, catecholamines (e.g., epinephrine, norepinephrine), which are direct indicators of sympathetic nervous system (SNS) activity, were absent from all included studies. Only one study (Gifford et al., 2019 [44]) included an adrenal function test as a proxy for overall HPA axis reactivity. Additional hormonal markers were assessed in several studies to provide insight into stress adaptation, including testosterone (Tomei et al., 2008 [67]), insulin-like growth factor (IGF-I) and inflammatory cytokines (Nindl et al., 2012 [56]), and lastly, estradiol, prolactin, and neuropeptide Y (Cho et al., 2017 [36]). However, sex hormones were infrequently evaluated despite their relevance to stress and reproductive health in women. Sympathoadrenal activity was measured during diverse occupational conditions, including military field training, selection courses, urban policing environments, and simulated combat. Elevated cortisol concentrations were generally observed in response to physical and psychological stress, and findings varied by training phase, environmental conditions, and participant experience level. However, despite this trend in heightened cortisol response to various stressors, we note that two studies observed declining cortisol over time (Lieberman et al., 2012 [52]; Strahler et al., 2015 [65]), which would suggest possible stress adaptations.

### 3.3. Body Composition

Twenty-nine studies (2912 women; age = 25.2 ± 6.6 y; BMI = 25.2 ± 2.2 kg/m^2^; BF = 28.3% ± 5.3%) were identified that either focused on or included anthropometrics and/or body composition as part of their primary outcomes. Notably, most studies included mixed samples, and only 11 (38%) focused exclusively on women. Studies were overwhelmingly conducted in military populations (82%) including those representative of the U.S., e.g., Army (West Point or Basic Combat Training) [33,34,52,53,62,64,66], Navy [22], Marine Corps [49,51], with only five studies (17%) focusing on law enforcement [35,40,41,50] and fire service [39]. Further, over three-fourths of included studies (79%) assessed women undergoing basic training or officer candidate school, with only a few involving active-duty personnel [22,33,35,38,40,41,47,57,66]. See Table 3 for the individual summaries of the coded dimensions for each study.

Four studies used a cross-sectional study design [33,35,41,66], with another two using a retrospective study design [40,50]; the remaining studies utilized a prospective cohort design. Studies that examined body composition outcomes achieved “moderate” reporting quality (63.0% ± 11.9%) despite widely varying scores (27–84%), and only two studies completely reported on ≥80% of checklist items (81.8%) [45,64].

Ten studies reported only anthropometric data [35,36,39,40,49,50,53,54,59], eighteen studies reported body composition outcomes [22,32,33,34,37,38,41,42,45,51,52,56,57,60,61,62,64,68,69], and one study focused on self-reported anthropometric data to calculate BMI [66]. Of the body composition assessment approaches, the methods employed (presented from most used to least) included dual energy X-ray absorptiometry [33,45,51,52,57,60,61,64], bioelectrical impedance [34,37,41,68,69], three- or four-site skinfolds [42,56,62], air displacement plethysmography (via BOD POD, Cosmed USA, Concord, CA, USA) [38], and deuterium oxide [32]. Notably, none of the studies used multi-compartment models to assess body composition.

Additional markers analyzed alongside anthropometrics and body composition included energetics (i.e., energy availability, intake, expenditure, and deficits) [32,34,39,57,59,60], metabolic health and metabolomics [34,45,51,52,60], nutritional status and dietary habits [33,53,59,61,62], physical fitness and athletic performance [22,33,34,37,38,40,41,42,49,50,53,55,57,61,66,68,69], physiological markers of health and stress (i.e., oxidative stress, systemic inflammation, reproductive health, bone health, and endocrine profile) [22,33,34,35,36,42,45,49,56,57,59,60,61,64,68], sleep [38,49,57], thermal strain [39], and psychological stress or mood state [49,50,51,52,53,66].

In general, body composition shifts were sensitive to tactical stressors, which led to either positive or negative impacts on anthropometric or body composition markers such as body mass and BMI, waist circumference, muscle mass/fat-free mass, BF/fat mass, and bone. Energy balance was one of the leading factors for how anthropometrics and body composition shifted around training. For example, tactical training events that included caloric restriction (in some cases severe) [32] combined with high energy expenditures were associated with high adrenal and oxidative stress, lower body weight, reduced bone formation, higher BF percentage, higher visceral adipose tissue, worsening metabolic health outcomes, decreased reproductive function, low energy availability (LEA) or nutrient-status symptoms, and/or higher systemic inflammation [32,33,35,36,45,49,53,56,57,59,60,64,66]. These effects were more pronounced among women entering training with a higher BMI. In contrast, tactical training events that resulted in positive body mass and body composition changes were associated with overall improvements in health, including (but not limited to) adequate energy availability, maintained or improved strength and power-based performance, and/or improved metabolic health and mood outcomes [51,55,61,62]. Overall, cardiorespiratory fitness and physical activity readiness assessment scores were associated with women’s body composition profile [41,42]. More specific to women’s health, women undergoing basic combat training on progestin-only contraceptives were found to have decreased bone mineral density and worsening bone health when compared to women on no contraceptive or the combined estrogen progestin oral contraceptive pill, but insight into these important interactions was only examined in one study [61]. Key body composition sex differences were observed. When compared to men, women tended to start military training with higher BF and lower muscle mass [57,69]. Women also tended to lose more total BF and/or preserve more muscle mass and maintain or regain lower body strength quicker compared to men undergoing the same tactical training [34,37,38,64,68].

### 3.4. Occupational Performance

This review included 21 studies (2,186 women; [Mean ± SD] age = 25 ± 5 y; BMI = 24.9 ± 1.8 kg/m^2^; BF = 29.4% ± 4.2%) that evaluated primary outcomes related to occupational performance in female tactical populations. Consistent with our other outcomes of interest, most studies focused on military settings (86%; 18 studies), and three focused on law enforcement [40,41,50]. The majority were conducted in military recruit or officer cadet cohorts (76%), with studies based in the U.S., United Kingdom, Israel, Norway, and South Korea. Only three studies involved a police population [40,41,50], and none were identified that assessed occupational performance in firefighting, EMS, or corrections populations. Notably, only one-third of studies (33%, seven studies) focused exclusively on women. See Table 4 for the individual summary of the coded dimensions for each study.

One-fourth (24%) of the included studies used a cross-sectional [33,41,47] or retrospective [40,50] design; the remaining studies were prospective cohort studies. Studies that examined occupational performance outcomes achieved “moderate” reporting quality (65.0% ± 8.3%) despite some variability in scores (52–81%), and only one study completely reported on ≥80% of checklist items (82.8%) [34].

Performance domains varied but commonly included anaerobic power (e.g., countermovement vertical jump), maximal force production (e.g., isometric mid-thigh pull, one-repetition maximum tests), military fitness assessments (e.g., Army physical fitness test, U.S. Marine Corps fitness tests), and tasks simulating tactical performance (e.g., casualty drag, water can carry, loaded march). Several studies [24,54,55] incorporated these field-based tasks along with assessment of physiological stress biomarkers (e.g., salivary cortisol). Eleven studies assessed body composition-related variables such as lean mass, fat mass, or BF percentage [33,34,37,38,41,42,56,57,61,68,69], while six studies reported BMI [22,40,49,50,53,55]. Notably, seven of the included studies assessed stress hormone concentrations in conjunction with performance outcomes [22,24,33,54,55,56,68]. Across studies, occupational performance metrics often declined during periods of high physiological strain, particularly under conditions of caloric deficit, sleep deprivation, or heavy cumulative training load, but generally recovered with adequate rest [57,68]. Only one study monitored recovery dynamics and contextual workload through wearable technology [55].

## 4. Discussion

The purpose of this scoping review was to synthesize the existing literature that comprehensively evaluated the impacts of stress on adrenal and neuroendocrine responses, body composition, and physical performance amongst women in demanding tactical occupations. A consistent theme was that occupation-related stress negatively impacted neuroendocrine, body composition, and performance outcomes. Sex differences in endocrine markers, energy metabolism, body composition profiles, and performance markers influenced the extent and manner by which tactical training impacted women. In addition to these contributions, this paper also provided key insights specific to the quality and reporting of studies in this research area. Notably, few studies achieved “high” reporting quality (no study achieved a perfect score), and on average, most were only considered to be of “moderate” reporting quality. For all studies included in this scoping review, one of the most poorly reported items was related to sample size determination (only nine studies completely reported on this item; see Table S3) [34,35,37,38,47,59,61,64,65]. The lack of sufficiently powered studies, with a high degree of reporting completeness, specific to women in tactical occupations, highlights a critical knowledge gap. The implications of our findings are summarized below, and when applicable, we have documented where knowledge is the weakest or poorly reported and have emphasized which research areas warrant additional investigation.

### 4.1. Adrenal Stress Response

The literature highlights both acute and chronic stress responses in women serving in tactical occupations. Tactical training courses are often designed to deliberately impose stress (i.e., stress inoculation), providing a controlled model to examine HPA axis and sympathoadrenal responses. Cortisol was consistently elevated in response to training stress, although longitudinal data suggest adaptive reductions in cortisol over time [52,54]. This potential adaptive response was also shown in two studies included in this review (Lieberman et al., 2012 [52]; Strahler et al., 2015 [65]). Sex differences in stress responses emerged across several studies. Women demonstrated heightened HPA axis sensitivity to adrenocorticotropic hormone stimulation [46], and cortisol remained elevated well into recovery after field exercises in female conscripts [68]. Cho et al. [36] reported alterations in reproductive hormones and stress-related neuropeptides during a 16-week officer training course, including decreases in estradiol and neuropeptide Y and increases in prolactin and cortisol, which corresponded with high rates of menstrual disruption. Further, we note the omission of estrogen as a marker of interest in studies evaluating stress response in women. Estrogen has been shown to modulate HPA axis activity in women, leading to heightened stress reactivity [23], which may help explain female-specific patterns of cortisol response.

Despite these insights, a major limitation of this literature is the absence of catecholamine measurement. Cortisol alone does not provide a complete picture of acute sympathoadrenal activity. Catecholamines (epinephrine, norepinephrine) are crucial for understanding short-term, SNS-driven stress responses, especially those linked to immediate operational performance. Their omission impairs the ability to evaluate the full spectrum of physiological stress responses. Several studies indirectly addressed the relationship between stress and performance. For instance, McFadden et al. [55] linked cortisol responses to physical performance metrics, but real-time integration of physiological and occupational performance data remains rare. Moreover, few studies considered environmental occupational stressors such as sleep deprivation, energy deficit, or urban exposure. Tomei et al. [67] observed higher testosterone concentrations in urban-exposed female police officers, potentially reflecting chronic environmental stress despite lacking cortisol or catecholamine data. Intersections between stress, metabolism, and immune function were noted in only a few studies. For example, Nindl et al. [56] found IGF-I concentrations increased while inflammatory markers decreased over the course of military basic training, suggesting positive adaptation. However, the role of stress hormone interactions (e.g., cortisol’s impact on IGF-I or immune function) was not evaluated. Conkright et al. [24] observed increased cortisol with suppressed growth hormone and IGF-I responses during a simulated operational stress protocol, supporting the need to study stress, metabolic, and immune interactions together. Furthermore, the reliance on salivary cortisol, while practical, presents limitations due to its high variability and lower specificity compared to plasma cortisol. A small number of studies used both methods, while only two incorporated alpha-amylase [31,65] as an index of psychological stress, despite its relevance.

### 4.2. Body Composition

Optimal body composition profiles, with an emphasis on supporting fat-free mass while maintaining healthy BF percentage, are hallmark components of the tactical athlete [70], leading to positive impacts on metabolic health [71], physical performance, and tactical occupation-specific tasks, such as load carriage, endurance, and strength [72]. Specific to women’s health, worsening body composition profiles are associated with reduced bone mineral density [73] and suboptimal reproductive function [74]. Body composition is highly energy and nutrient-sensitive, which was evident in the available literature, where muscle mass decreased in training environments that included particularly high energy expenditures, low quantity intake (caloric restriction/energy deficit), and/or low-quality nutrient intake. These outcomes were consistently associated with increased systemic inflammation, reduced metabolic health, and decreased performance, all of which led to negative impacts on readiness and resiliency.

A major observation in the current literature was the connection of energy balance to body composition profiles. The importance of energy balance was reflected in many of the LEA-related markers reported, such as decreased muscle mass, despite LEA itself not being a focal area for most of the studies. Traditionally, LEA has been most studied in the context of exercise and sport; however, women in tactical environments may be at increased risk for LEA and its downstream, deleterious consequences. A recent review by O’Leary et al. highlighted that LEA, common during intense field training, can trigger Relative Energy Deficiency in Sport (RED-S) in soldiers, which can lead to serious physiological and psychological sequelae [75]. Emphasizing this point, select studies in this review reported some of the more severe LEA phenotypes [76] such as reduced sleep quality, reduced reproductive function, training-induced anovulation [45], and poor bone metabolism [59]. Interestingly, these negative outcomes were observed in both acute and chronic training environments. Although LEA is typically discussed as a more chronic condition, even acute bouts of LEA (less than 5 days) can lead to physiological disruptions in women, such as negative impacts on metabolic health, musculoskeletal health, and hormonal profiles [76,77], and can eventually lead to reproductive health dysfunction. This is particularly relevant in tactical settings where undulating periods of highly stressful occupational demands combined with energy deficits may spiral into LEA. Indeed, Cho et al. [36] reported that a 16-week military training led to menstrual disruptions, including amenorrheic episodes for some women, coupled with hormonal changes, as has also been demonstrated by O’Leary and colleagues in their cross-sectional study of menstrual disturbances in British Service women [78]. These works underscore the broader physiological implications of LEA and the interconnectedness of training-specific stressors on anthropometrics, body composition, and reproductive health in women in tactical professions.

While LEA is composed of a series of physiological dysfunctions [79,80], it does not necessarily result in performance decrements [81,82], making it difficult to recognize. Identifying other objective and reliable markers to track LEA symptoms, such as body composition changes, is vital. Future research investigating how and to what extent chronic stress and energy deficits together negatively impact musculoskeletal health and body composition can help develop appropriate intervention strategies in this population. Furthermore, providing education on adequate energy availability, how to recognize LEA signs and symptoms, and offering a framework and solutions aimed at addressing LEA [79] is of great benefit to women in the tactical space, especially as women tend to be at higher risk for developing RED-S [83].

A unique theme in the available literature was women’s ability to preserve muscle mass and preferentially rely on adipose stores for energy (evidenced by BF percent changes with training), an advantage related to sex differences in energy metabolism. Several studies found that while women began training with a higher BF percentage, they tended to lose more BF and less muscle mass when compared to men [34,37,38,64,68]. One reason for this body composition shift may be due to women’s preferential reliance on fat for energy, specifically during exercise, reducing the need to turn to muscle breakdown for energy production [27]. Countless benefits are connected to maintaining muscle mass, emphasizing the importance of developing tactics aimed at supporting healthy body composition in this population. In addition to sustained performance, the ability to preserve and maintain skeletal muscle is closely tied to fatigue resiliency, improved metabolic (and overall) health, improved management of inflammation and oxidative stress, improved recovery processes [27], reduced injury risks [84], and improved cognitive function [85], all essential components of readiness. This observation opens the door for several different future research directions to better understand how to capitalize on women’s advantageous differences in energy metabolism. These include tactical-specific training and dietary approaches to support, maintain, and increase muscle mass, both in general settings and especially in adapting to stressful environments.

As expected, a wide range of methodologies were used in this body of work, with a mix of two- and three-compartment models used to assess body composition. Additionally, very few details were reported on the testing environment in which the assessments were collected. These inconsistencies bring an additional layer of consideration when comparing results across studies. Body composition assessments are highly sensitive to the testing environment, and results can be impacted by the assessment method itself, hydration state (i.e., dehydrated vs. euhydrated), nutrient status (i.e., fasting vs. postprandial) [86], environmental stress [87], medications (especially those that impact fluid balance), and more. It is also important to consider that body composition may be assessed in laboratory, clinical, and even field settings in this population, further introducing potential confounding variables that may impact results. Researchers should consider assessment tools that are valid, accurate, as well as feasible and appropriate for the testing environment. Future investigations may consider exploring how multi-compartment models for body composition can be included in this line of work, which would offer more precise and reliable assessments [88].

### 4.3. Occupational Performance

The literature on women’s occupational performance in tactical settings emphasizes assessments during recruit training or structured short-term courses. While these environments offer controlled opportunities to evaluate performance and adaptation, they do not fully capture operational readiness across the career lifespan or under real-world occupational demands. Load carriage emerged as a recurring and central theme in many studies (e.g., Conkright et al., 2021 [24]; O’Leary et al., 2023 [59]), reflecting its importance in military and first responder tasks. However, very few studies assessed how repeated or cumulative load carriage, especially under caloric or sleep deficits, impacts recovery and injury risk in women over time.

While some studies measured anaerobic power and task-specific performance, gold-standard strength assessments (e.g., one- or three-repetition maximum) were often absent, likely due to field testing constraints. This limits our understanding of absolute strength capacity and how it may support or constrain tactical readiness in women. In contrast, McFadden et al. [54] included both countermovement jumps and isometric mid-thigh pulls, paired with sleep, stress, and workload monitoring, offering a more complete view of performance under stress. Studies such as Vikmoen et al. [68] and Øfsteng et al. [57] show that women’s performance in anaerobic tasks declines in military training conditions but recovers with rest. However, recovery trajectories and resilience thresholds in female tactical personnel remain underexplored.

Perhaps most notably, few studies incorporated comprehensive physiological assessments, including stress markers, body composition, workload, and sleep data. McFadden et al. [54] and Conkright et al. [24] are exceptions, linking workload with hormonal and performance outcomes. Still, many studies did not evaluate key variables such as lean body mass or hormonal cycles, factors especially relevant to women’s performance and recovery. Preserving lean body mass is crucial in women. While women typically present lower baseline muscle mass, specific and personalized strength training programs can improve tactical occupation-relevant physical variables, including muscular strength and subsequent performance outcomes [89,90], supporting the need for comprehensive physiological assessments in this line of research. We also note a significant lack of data on women in non-military tactical populations. Indeed, no studies assessed occupational performance in firefighting (including wildland fire), EMS, or corrections. These settings involve unique demands (e.g., heat exposure, shift work, prolonged incident response) and may present sex-specific risk factors related to thermoregulation, cardiovascular strain, or reproductive suppression.

### 4.4. Limitations

Certain limitations must be applied to the current scoping review. First, we performed a critical appraisal of individual evidence to gauge the completeness of reporting (i.e., reporting quality) using existing checklists (e.g., The STROBE and CONSORT Checklists). Although not all scoping reviews include a critical appraisal of evidence, we acknowledge that our evaluation does not *directly* address methodological quality or risk of bias. Nonetheless, we posit that the quality and completeness of reporting by authors are related to higher quality and more robust research and are necessary for developing evidence-based recommendations. Importantly, we also note that a clear consensus on what tool, scale, or approach should constitute the ‘gold standard’ in assessing the quality of evidence included in scoping reviews has yet to emerge [28], particularly for research involving tactical occupations. Another potential limitation is that our search strategy may not have identified all potentially eligible sources of evidence despite using multiple databases and platforms. However, given the paucity of data involving women in tactical occupations, we are confident that we have identified the relevant research aligned with the objectives of this scoping review. Finally, the sheer volume of literature (or lack thereof) that explicitly examines the unique physiological and performance-related demands placed on women in tactical occupations is a major limitation of the literature, beyond this scoping review. While there has been progress in this regard, there are persistent critical gaps that must be addressed.

One of the major limitations of this line of research is the lack of female reproductive health insight despite its connection to the adrenal/neuroendocrine system, energy balance and body composition, and physical performance outcomes. This was a uniformly identified gap in the research studies included in this review. Most studies failed to incorporate, and much less stratify by, information on contraception use and/or type, menstrual cycle phase details, reproductive health state (i.e., menstruating, pregnant, peri-menopausal, or menopausal), or reproductive health insights in general (amenorrheic, menstrual cycle dysfunction, reproductive hormone disruption, etc.). Including reproductive health data would provide much-needed context for the observed stress-influenced outcomes. While incorporating reproductive health measures is not yet standard practice in the field, recent works have commented on the critical need to include female reproductive health markers, properly identify reproductive health stage, and track menstrual cycle phase to strengthen female athlete research [91]. This need extends to female tactical populations, as military training has been associated with marked reproductive and menstrual cycle dysfunction [92]. Future research that includes both subjective and objective measures associated with reproductive physiology is desperately needed.

### 4.5. Evidence-Informed Recommendations for Future Research

**Figure 3:**
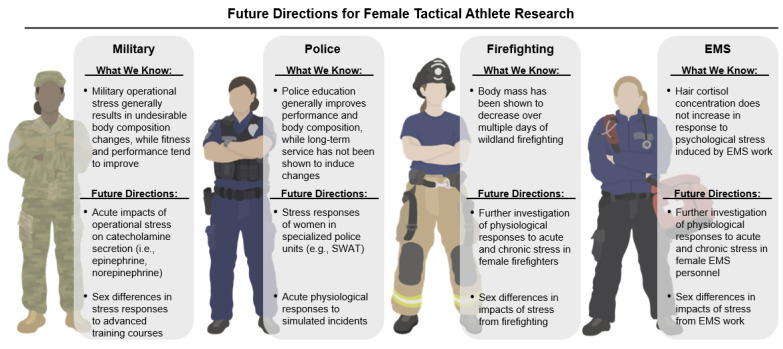
Known responses to occupational stressors within each tactical domain and directions for future research regarding female tactical athletes. We note the lack of data specific to women in the corrections tactical domain, highlighting this domain’s importance for future research directions. EMS, Emergency Medical Services. SWAT, Special Weapons and Tactics unit. This image was created using Procreate^®^ (version 5.3.15) and PowerPoint (version 2.98.4).

The available research lays the groundwork for a wide span of future directions investigating the impact of stress on female physiology, body composition, and performance amongst women in tactical occupations. Future research must characterize women’s overall health, body composition, fitness, and performance profiles in tactical domains in depth. Even more pressing, research must explore how women’s profiles shift in both acute and chronic stressful tactical environments. This comprehensive approach allows for broader contextual insight into the unique phenotypes associated with women in tactical occupations and more closely identifies areas of both strength and concern for more targeted discoveries. For example, works investigating how adrenal and neuroendocrine biomarkers, such as catecholamines, shift with occupational stress and potentially impact metabolic and immune responses would add much-needed depth relative to the available cortisol data in this population. The inclusion of more specific and robust assessment techniques such as body composition measurements that include multi-compartment models and surveying LEA, its associated risk factors, and RED-S incidences would further strengthen this line of work. Targeted interventions specific to women in tactical occupations should address energy and nutrient deficiencies [26] and include structured physical fitness and strength and conditioning approaches aimed at supporting and enhancing skeletal muscle mass specific to women in tactical occupations [93,94,95]. Future research specific to tactical women must incorporate key measures associated with occupational readiness and should be aimed at a comprehensive understanding of the relationship between resiliency, muscle mass, and women’s health. These areas can, and must be, combined with comprehensive reproductive health assessments that include both objective and subjective measures. Finally, ample opportunity lies in expanding tactical research beyond military populations to other tactical domains where we know far less, such as firefighting, EMS, and corrections. While some domain-specific challenges in this space have been identified, the breadth and depth of this area of research is severely lacking. Addressing these knowledge gaps would provide much benefit and further emphasize awareness and understanding of occupation-specific demands and stressors. Our current understanding of the impact of stress on adrenal and neuroendocrine responses, body composition, and performance amongst women within each tactical domain and the areas for future research in these tactical spaces are summarized in Figure 3.

## 5. Conclusions

This scoping review highlights the progress in evaluating how stress affects occupational performance in women serving in tactical roles, but our understanding remains incomplete due to methodological and conceptual limitations in the literature. The extent of the consensus found 40 studies of moderate reporting quality with overarching themes focused on how stress dysregulates the HPA axis, has mixed effects on body composition, with most as adverse, and results in performance declines, particularly under heavy external loads and energy deficits. Current studies emphasize early-career military cohorts and field-based testing methods, with limited integration of comprehensive physiological monitoring or stress–response data. Cortisol, while widely used as a measure of adrenal stress response, cannot substitute for a comprehensive assessment of sympathoadrenal activity, especially in the absence of catecholamine measurement and physical performance correlation. While body composition is closely associated with tactical health, performance, and recovery outcomes, assessment discrepancies, coupled with a lack of dietary intake and energy expenditure insight, result in conflicting outcomes and an incomplete understanding of this performance metric relative to women’s tactical performance. Findings specific to occupational performance suggest that anaerobic power and task-specific performance decline under stress but may recover with appropriate rest. Lean body mass likely contributes to performance potential, yet its role is underassessed.

Key gaps include a lack of integration of sympathoadrenal stress responses (i.e., catecholamines) and evaluation of associated metabolic and immune responses, underrepresentation of female-specific hormonal and reproductive health measures, limited contextualization of stress responses in operational settings (e.g., sleep deprivation or energy deficit), and sparse linkage between stress biomarkers and occupational readiness or injury outcomes.

As women comprise a growing proportion of tactical professionals, including military, police, fire service, EMS, and corrections, tailored research is needed to address their unique physiological demands [96]. Holistic research strategies are needed to capture the complexity of performance readiness in women, integrating stress and muscle physiology, reproductive health, energy and nutrient balance, and resilience in realistic operational contexts. Comprehensive assessment of these interrelated aspects of performance readiness addresses current knowledge gaps and is vital for informing policy, improving readiness, and enhancing the health and career longevity of female tactical personnel.

## Figures and Tables

**Table 1:** Summary of included studies for the total sample and by tactical occupation.

	Total (*k =* 40) *N* = 3693				Military (*k* = 32) *n* = 2702				Police (*k* = 6) *n* = 864				EMS (*k* = 1) *n* = 28				Fire (*k* = 1) *n* = 3			
	*k*	M	SD	Min, Max	*k*	M	SD	Min, Max	*k*	M	SD	Min, Max	*k*	M	SD	Min, Max	*k*	M	SD	Min, Max
Age (y)	38	26.9	7.5	18.8, 47.6	30	24.4	5.4	18.8, 44.1	6	35.6	4.4	27.5, 39.6	1	47.6	9.4	-	1	26	3	-
Recruits/ Cadets, *n* (%)	28	*3228*	87.4%		24	*1917*	70.9%		1	*682*	78.9%		0	*0*	0%		0	*0*	0%	
Servicewomen, *n* (%)	12	*953*	25.8%		3	*673*	24.9%		4	*249*	28.8%		1	*28*	100%		1	*3*	100%	
Service (y)	6	8.2	4.1	3.0, 14.1	2	3.2	0.2	3.0, 3.3	4	9.8	2.4	7.4, 13.1	1	14.1	8.2	-	1	6	2	-
Body mass (kg)	31	67.1	5.6	60.9, 82.3	33	66.8	5.6	60.9, 82.3	3	72.2	6.2	67.8, 76.5	-				1	66.7	4.4	-
BF (%)	14	28.3	4.9	17.2, 36.0	13	27.9	4.8	17.2, 36.0	1	33.7		-	-				-			
BMI (kg/m^2^)	26	25.1	2.1	22.4, 30.2	25	25.1	2.1	22.4, 30.2	6	25.4	2.2	23.0, 28.0	-				1	24.3	1.7	-
WC (cm)	2	76.0	6.3	71.5, 80.4	1	71.5		-	1	80.4		-	-				-			
VO_2max_ (mL/kg/min)	9	36.0	4.3	27.2, 40.5	6	36.5	7.0	32.5, 40.5	3	35.2	7.0	27.2, 40.4	-				-			

BF, Body fat. BMI, Body mass index. EMS, Emergency medical services. *k*, Number of studies. M, mean. Max, Maximum. Min, Minimum. *N*, Total number of women included in the scoping review. *n*, Number of women included in the scoping review within the tactical domain. SD, Standard deviation. VO_2max_, Maximal oxygen uptake. WC, Waist circumference.

**Table 2:** Summary of the studies included in the scoping review that evaluated adrenal stress and neuroendocrine responses.

Author, Year	Tactical Domain and Study Characteristics	Sample Characteristics	Adrenal and Neuroendocrine Responses		Other Markers Analyzed	Impact of Stress and Sex on Adrenal and Neuroendocrine Responses
			Outcomes and Assessment Details	Aggregate-Level Study Data (Mean ± SD) ^a^		
Andrews et al., 2010 † [33] RQ: 51.6%	Military Design: Cross-sectional; service members completing the Army Physical Fitness Test (Washington, DC, USA) Primary outcomes: Oxidative stress	60 overweight or obese active-duty service members (35 M, 25 F) M: 33.1 ± 8.3 y, 99.8 ± 9.9 kg, 31.9 ± 2.8 kg/m^2^ F: 34.4 ± 7.4 y, 82.3 ± 11.0 kg, 29.9 ± 2.3 kg/m^2^	Oxidative stress biomarkers: Creatine kinase, C-reactive protein, glutathione peroxidase, superoxide dismutase Methods: Serum (Creatine kinase and C-reactive protein) and plasma (glutathione peroxidase and superoxide dismutase) Fasted status: NR	Creatine kinase, U/L (F): Pre (*n* = 18): 117.0 ± 57.3 Post (*n* = 17): 153.9 ± 63.5 Post 24 h (*n* = 7): 169.0 ± 70.2 * C-reactive protein, mg/dL (F): Pre (*n* = 17): 0.29 ± 0.28 Post (*n* = 14): 0.29 ± 0.23 Post 24 h (*n* = 7): 0.34 ± 0.27 Glutathione peroxidase, ng/dL (F): Pre (*n* = 17): 76.2 ± 42.0 Post (*n* = 17): 70.8 ± 29.3 Superoxide dismutase, ng/dL (F): Pre (*n* = 16): 0.80 ± 0.62 Post (*n* = 16): 0.95 ± 0.63 * *p* < 0.05 (time)	Baseline: Body composition, fitness level, dietary intake	The Army Physical Fitness Test induces oxidative stress in male and female overweight soldiers. Soldiers could potentially accrue immeasurable cellular damage from exercise-induced oxidative stress. Modification of dietary intake, fitness level, and body composition may mitigate the negative effects of long-term oxidative stress during military exercise.
Cho et al., 2017 [36] RQ: 42.4%	Military Design: Prospective cohort (8 wk); during 16-wk Officer training course at the Korea Third Military Academy (Yeongcheon, South Korea) Primary outcomes: Reproductive function	40 women cadets 22–28 y, 63.6 ± 7.8 kg, 24.1 ± 2.7 kg/m^2^, waist circumference = 71.5 ± 7.0 cm; regular menstrual cycles	Hormones: Cortisol, CRH, estradiol Methods: Serum Fasted status: Overnight	Cortisol, μg/dL: Baseline: 16.1 ± 3.9 Wk 4: 18.1 ± 2.2 Wk 8: 18.7 ± 2.2 * CRH, pg/dL: Baseline: 84.4 ± 65.1 Wk 4: 57.7 ± 28.3 Wk 8: 22.0 ± 21.7 * Estradiol, pg/dL: Baseline: 106.0 ± 120.7 Wk 4: 44.6 ± 24.4 Wk 8: 55.1 ± 43.1 * * *p* < 0.01 (time)	Reproductive function: regularity, prolactin, endorphin-β, NPY, leptin, orexin-A, ghrelin, follicle-stimulating hormone, luteinizing hormone, thyroid-stimulating hormone, thyroxine	Cortisol, prolactin, and thyroid-stimulating hormone increased in response to intensive military training, but CRH endorphin-β, NPY, orexin-A, ghrelin, estradiol, and thyroxine decreased. Outcomes assessed were not different between women with normal menstruation and women with irregular menstruation.
Conkright et al., 2021 ‡ [24] RQ: 57.6%	Military Design: Prospective cohort; 5-day simulated military operational stress protocol (Pittsburgh, PA, USA) Primary outcomes: Neuromuscular performance, mood state, and hormonal responses	69 healthy U.S. service members (54 M, 15 F); 4.3% Air Force, 81.2% Army, 8.7% Marine Corps, 5.8% Reserve Officers’ Training Corps M: 26.4 ± 5.3 y, 85.2 ± 14.0 kg, BF = 20.2 ± 7.1%, VO_2peak_ = 47.8 ± 7.6 mL/kg/min F: 25.6 ± 5.6 y, 67.0 ± 9.0 kg, BF = 27.4 ± 7.2%, VO_2peak_ = 40.5 ± 5.0 mL/kg/min	Hormones: Cortisol, IGF-1 Methods: Serum (PRE and POST tactical mobility test) Fasted status: Overnight	Cortisol, μg/dL (PRE) (F): Day 1: 14.4 ± 3.8 Day 3: 12.6 ± 4.7 Day 4: 12.7 ± 4.3 Cortisol, μg/dL (POST) (F): Day 1: 23.5 ± 5.7 * Day 3: 26.8 ± 6.5 * Day 4: 25.1 ± 9.2 * IGF-1, ng/mL (PRE) (F): Day 1: 409.3 ± 118.9 Day 3: 353.1 ± 93.3 Day 4: 321.7 ± 95.8 IGF-1, ng/mL (POST) (F): Day 1: 397.7 ± 93.3 Day 3: 360.6 ± 108.1 * Day 4: 335.8 ± 98.2 *^,^** * *p* < 0.05 (time, POST vs. PRE), ** *p* < 0.05 (day)	Neuromuscular performance: Lower body power, tactical mobility test Mood state: POMS subscales (tension, depression, anger, fatigue, confusion, vigor) Other hormones: Growth hormone, brain-derived neurotrophic factor	Changes in mood and hormone concentration were associated with physical performance outcomes. Hormones associated with anabolic status and energy metabolism were expressed differently between sexes. Changes in mood and hormones were associated with physical performance.
Flegr et al., 2012 [43] RQ: 43.8%	Military Design: Cross-sectional; psychological performance battery as part of entrance examination (Central Military Hospital, Prague, Czech Republic) Primary outcomes: Psychological health and performance, hormones	193 Czech military personnel (100 M, 93 F) M: 27.9 ± 7.9 y F: 29.2 ± 7.3 y	Hormones: Cortisol, testosterone, estradiol Methods: Serum Fasted status: NR	Cortisol, nmol/L (F): 728 ± 121 * Testosterone, nmol/L (F): 1.10 ± 3.87 * Estradiol, nmol/L (F): 0.29 ± 0.03 * * *p* < 0.001 (sex)	Psychological health: Questionnaires (N-70, OD-1, Buss–Dürker Inventory) Psychological performance: Meili selective memory test, TOPP test (attention and short-term memory), Wiener Matrizen-Test, OTIS test (verbal intelligence)	In female military personnel, cortisol was associated with hypochondria, psychopathology, and aggression. Testosterone was associated with hypochondria, psychastheny, indirect aggression, irritability, and paranoia. Estradiol was associated with phobia and negativism. In male military personnel, cortisol was correlated with emotion and impulsivity, while testosterone was associated with psychopathology and paranoia, and estradiol with psychopathology.
Gifford et al., 2019 ‡ [44] RQ: 78.8%	Military Design: Prospective cohort (11 months); Commissioning Course (infantry-based training) at the Royal Military Academy (Sandhurst, UK) Primary outcomes: HPA axis function, mental health Part of the Female Endocrinology in Arduous Training (FEAT) Study	52 women recruits 24.0 ± 2.5 y	Hormones: Cortisol Methods: HCC and salivary cortisol (measured AM and PM), plasma cortisol (measured in AM, separated into non-CCP vs. CCP users) Fasted status: Overnight (plasma only)	HCC, pg/mg (ln): Month 1: 2.0 ± 0.9 * Month 2: 2.1 ± 0.8 * Month 3: 2.1 ± 1.0 * Month 4: 2.0 ± 1.1 * Month 5: 2.0 ± 0.9 Month 6: 2.2 ± 0.7 Month 7: 2.2 ± 0.9 Month 8: 2.1 ± 0.9 Month 9: 2.2 ± 0.9 * Month 10: 2.4 ± 0.9 * Month 11: 2.4 ± 0.7 * Month 12: 2.2 ± 0.9 * Cortisol (saliva), μg/dL ** T1: Wk 1 = 0.4 ± 0.3, Wk 7 = 0.6 ± 0.2, Wk 14 = 0.5 ± 0.3 T2: Wk 1 = 0.6 ± 0.3, Wk 5 = 0.5 ± 0.1, Wk 14 = 0.4 ± 0.3 T3: Wk 1 = 0.5 ± 0.2, Wk 5 = 0.5 ± 0.2, Wk 14 = 0.4 ± 0.2 Cortisol (plasma), nmol/L: non-CCP users ** T1: Wk 1 = 701.0 ± 134.6 T2: Wk 14 = 669.3 ± 162.4 T3: Wk 13 = 558.4 ± 182.2 CCP users ** T1: Wk 1 = 1061.4 ± 198.0 T2: Wk 14 = 966.3 ± 166.4 T3 Wk 13 = 855.4 ± 190.1 * *p* < 0.05 (time, vs. pre-6 to pre-4), ** *p* < 0.001 (main effect, time)	Mental health: Anxiety, depression, resilience	Obvious psychological and physical stress was observed in early training and was quickly followed by habituation. No evidence of HPA axis maladaptation was observed, which is beneficial for women undertaking intense military training.
Gifford et al., 2025 ‡ [46] RQ: 72.7%	Military Design: Prospective cohort (11 months); Commissioning Course (infantry-based training) at the Royal Military Academy (Sandhurst, UK) Primary outcomes: HPA axis function, HPG axis function Part of the Female Endocrinology in Arduous Training (FEAT) Study	78 Officer Cadets (10 M, 68 F) 24.9 ± 2.9 y, 66.7 ± 8.2 kg	Hormones: Cortisol Methods: HCC, saliva, plasma (in response to 1 μL ACTH over 1 h) Fasted status: Overnight (plasma)	Ln-HCC, pg/mg (F): Month 0: 2.1 (0.2) Month 1: 2.3 (0.1) Month 2: 2.1 (0.2) Month 3: 2.0 (0.2) Month 4: 1.8 (0.2) Month 5: 2.1 (0.2) * Month 6: 2.2 (0.1) * Month 7: 2.0 (0.2) * Month 8: 2.2 (0.2) Month 9: 2.3 (0.2) * Month 10: 2.4 (0.2) * Month 11: 2.1 (0.2) * Cortisol, μg/dL—saliva (F): Wk 1: AM = 0.45 (0.06) vs. PM = 0.11 (0.01) Wk 8: AM = 0.62 (0.05) * vs. PM = 0.09 (0.03) Wk 14: AM = 0.55 (0.05) vs. PM = 0.09 (0.02) Wk 16: AM = 0.58 (0.06) vs. PM = 0.09 (0.02) Wk 20: AM = 0.47 (0.04) * vs. PM = 0.11 (0.03) Wk 29: AM = 0.44 (0.05) vs. PM = 0.15 (0.03) Cortisol, nmol/L—plasma (F) * Wk 1 Min 0: 197.0 (24.1) Wk 29 Min 0: 255.6 (53.0) Wk 1 Min 20: 512.1 (27.9) Wk 29 Min 20: 574.1 (23.4) Wk 1 Min 30: 564.2 (35.5) Wk 29 Min 30: 672.2 (25.9) Wk 1 Min 40: 532.4 (35.6) Wk 29 Min 40: 540.7 (18.6) Wk 1 Min 60: 466.3 (30.5) Wk 29 Min 60: 479.0 (18.5) * *p* < 0.05 (sex × time)	Other hormones: Gonadotrophins (follicle-stimulating hormone, gonadotrophin-releasing hormone, luteinizing hormone)	HPA axis responses to intense military training were greater in women vs. men, while HPG responses appear to be down-regulated in women and not men. Cortisol progressively increased in women in response to stress, but not in men.
Johnsen et al., 2023 [48] RQ: 71.9%	Emergency medical services Design: Cross-sectional Primary outcomes: Physiological and psychosocial stress	79 ambulance workers (51 M, 28 F) in southern Sweden M: 47.7 ± 9.2 y, 19.8 ± 11.0 y experience; 82% registered nurses, 18% emergency medical technicians F: 47.6 ± 9.4 y, 14.1 ± 8.2 y experience; 93% registered nurses, 7% emergency medical technicians	Hormones: Cortisol Methods: HCC Fasted status: NR	Cortisol, pg/mg (F): 23.5 [IQR: 11.6–47.0] *p* = 0.719 (sex)	Psychosocial stress: 17-item Demand–Control–Support Questionnaire	HCC was not different between ambulance personnel and a population-based reference sample for men or women. There was no difference in HCC between men and women, and work-related factors were not associated with HCC.
Lieberman et al., 2008 [51] RQ: 66.7%	Military Design: Prospective cohort (13 wk); U.S. Marine Corps basic training (Parris Island, SC, USA) Primary outcomes: Body composition, metabolic status, mood state	50 women recruits 19.7 ± 2.1 y, 63.9 ± 0.8 kg, FM = 19.5 ± 0.6 kg, FFM = 41.7 ± 0.5 kg, BF = 30.2 ± 0.7%	Hormones: Cortisol Methods: Serum Fasted status: Overnight	Cortisol, μg/dL: Wk 1: 13.2 ± 0.7 Wk 12: 10.4 ± 0.7 *p* < 0.003 (time)	Body composition: BM, FM, FFM, BF, BMM Metabolic status: Cholesterol (total, LDL, HDL), free fatty acids, glucose Mood state: POMS subscales (fatigue, confusion, depression, tension, anger, vigor)	U.S. Marine Corps training provokes considerable changes in several biomarkers associated with nutrition and physical status, including LDL, free fatty acids, and cortisol. U.S. Marine Corps recruit training substantially modifies the physical and psychological state of female trainees in a manner similar to that identified by U.S. Marine Corps recruit training doctrine.
Lieberman et al., 2012 [52] RQ: 60.6%	Military Design: Prospective cohort (12 wk); U.S. Marine Corps basic training (Parris Island, SC, USA) Primary outcomes: Body composition, mood state, metabolic status	35 women recruits 19.3 ± 1.7 y, 23.1 ± 1.8 kg/m^2^	Hormones: ACTH Methods: Serum Fasted status: Overnight	ACTH, pg/mL: Pre: 16.2 ± 9.7 Post: 15.4 ± 8.0 *p* = 0.583 (time)	Body composition: BM, FM, LM, BMM Mood state: POMS subscales (fatigue, confusion, depression, tension, anger, vigor) Metabolic status: Substance P, fructosamine, cholesterol (total, HDL, LDL), triglycerides, free fatty acids, DHEA-S	Increased LDL cholesterol, triglycerides, fructosamine, and ACTH were associated with better overall mood. ACTH is not typically associated with mood; however, its administration has been reported to provoke psychological benefits.
McFadden et al., 2024a ‡ [54] RQ: 72.7%	Military Design: Prospective cohort (13 wk); U.S. Marine Corps basic training (Parris Island, SC, USA) Primary outcomes: Sex differences in workload, sleep, stress, and performance Part of a larger study, the U.S. Marine Corps Gender-Integrated Recruit Training study	281 recruits (182 M, 99 F); healthy, naïve to military life 19 ± 2 y, 64.1 ± 7.1 kg, 23.3 ± 2.1 kg/m^2^, FM = 15.6 ± 3.8 kg, FFM = 48.5 ± 5.3 kg	Hormones: Cortisol Methods: Saliva Fasted status: NR	Cortisol, μg/dL: Wk 2: 0.78 (0.03) Wk 7/8: 0.63 (0.02) Wk 11: 0.77 (0.07) *p* = 0.01 (sex × time)	Performance: Lower body strength and power Workload: Relative energy expenditure, distance, steps Sleep: Continuity and duration	The greatest physical demands occur earlier in the training program, yet the stress response was maintained throughout the training. Women experienced significantly higher cortisol and sleep continuity than men.
McFadden et al., 2024b [55] RQ: 72.7%	Military Design: Prospective cohort (11 wk); U.S. Marine Corps basic training (Parris Island, SC, USA) Primary outcomes: Performance, resilience, wearable tracking Part of a larger study, the U.S. Marine Corps Gender-Integrated Recruit Training study	196 recruits (97 M, 99 F) Baseline characteristics NR	Hormones: Cortisol Methods: Saliva Fasted status: NR	Cortisol, μg/dL: Wk 2: 0.8 ± 0.4 Wk 7: 0.6 ± 0.3 Wk 11: 0.8 ± 0.7 *p*-value NR	Performance: U.S. Marine Corps—specific performance, lower body strength, and power Resilience: Connor–Davidson Resilience Scale, workload, self-reported sleep, stress Wearable tracking: energy expenditure, distances, sleep, acceleration	Increased cortisol was negatively associated with workload (energy expenditure and distance) during basic recruit training. This indicates that recruits who managed stress better may be able to perform more work than recruits who exhibited exaggerated stress responses.
McGraw et al., 2013 † [31] RQ: 64.3%	Military Design: Quasi-experimental (within-subjects, repeated measures); 10-min combat casualty simulation Primary outcomes: Biological reactivity	38 (10 M, 28 F) Army nurses 28.5 ± 6.5 y, physical fitness test score = 247 ± 52, 79% < 12 months of military nursing experience	Hormones: Cortisol, α-amylase Cardiovascular: HR, SBP, DBP Methods: Saliva (cortisol, α-amylase); measured at baseline (−20 min), immediately pre-simulation (−5 min), midway simulation (+5 min), post-simulation (+10 min), and during recovery (+20 min and +40 min) Fasted status: NR	Cortisol, μg/dL (F): Baseline (−20 min): 0.2 ± 0.1 Pre (−5 min): 0.2 ± 0.1 Mid (+5 min): 0.2 ± 0.1 * Post (+10 min): NR Post 2 (+20 min): 0.2 ± 0.1 * Post 3 (+40 min): 0.2 ± 0.1 * α-amylase, U/mL (F): Baseline (−20 min): 122.1 ± 69.7 Pre (−5 min): 136.6 ± 82.4 * Mid (+5 min): 193.1 ± 142.9 * Post (+10 min): NR Post 2 (+20 min): 141.3 ± 120.6 Post 3 (+40 min): 117.7 ± 91.5 HR, beats/min (F): Baseline (−20 min): 78.9 ± 13.7 Pre (−5 min): 81.6 ± 15.3 * Mid (+5 min): 126.9 ± 18.3 * Post (+10 min): 89.9 ± 17.1 Post 2 (+20 min): 84.9 ± 13.4 Post 3 (+40 min): 76.6 ± 12.8 SBP, mmHg (F): Baseline (−20 min): 116.0 ± 11.0 Pre (−5 min): 128.1 ± 12.7 * Mid (+5 min): NR Post (+10 min): 128.6 ± 11.4 Post 2 (+20 min): 118.6 ± 11.2 Post 3 (+40 min): 115.9 ± 10.3 DBP, mmHg (F): Baseline (−20 min): 74.5 ± 8.5 Pre (−5 min): 80.2 ± 8.4 * Mid (+5 min): NR Post (+10 min): 81.7 ± 7.9 Post 2 (+20 min): 76.0 ± 8.4 Post 3 (+40 min): 73.9 ± 9.3 * *p* < 0.01 (time, vs. baseline)	None	Age, gender, perceived difficulty of the simulation, and previous nursing experience were associated with differences in physiological stress responses. Men experienced greater stress response (cortisol levels) but a more rapid recovery (*p* < 0.01 for both) than women. Men also displayed a more rapid recovery for SBP (*p* = 0.03) than women. Individual perceptions of performance, stress, and task difficulty were associated with the degree of reactivity to and recovery from the simulated combat casualty.
Nindl et al., 2012 [56] RQ: 57.6%	Military Design: Prospective cohort (~4 months); Israeli Defense Force gender-integrated basic recruit training program (Tel Hashomer, Israel) Primary outcomes: Body composition, inflammation, fitness	194 (29 M, 93 F) recruits M: 19.1 ± 1.3 y, 72.6 ± 2.7 kg, VO_2max_ = 51.6 ± 1.1 mL/kg/min F: 18.8 ± 0.6 y, 61.6 ± 0.6 kg, VO_2max_ = 36.8 ± 0.7 mL/kg/min Collected in conjunction with Evans et al., 2008 [42]	Hormones: IGF-1, free IGF-1 Methods: Serum Fasted status: Overnight	IGF-1 (F): Pre: 470.0 (15.8) ng/mL Post: 524.6 (15.3) ng/mL *p* > 0.05 (sex) *p* < 0.05 (time) Free IGF-1 (F): Pre: 0.49 (0.04) ng/mL Post: 0.52 (0.05) ng/mL *p* > 0.05 (Sex) *p* > 0.05 (Time)	Body composition: BM, FM, FFM, BF Inflammation: IL-1β, IL-6, TNF-α, IGFBP-1, IGFBP-2, IGFBP-3, IGFBP-4, IGFBP-5, IGFBP-6	IGF-1 responses to basic military training were similar between sexes. Fitness level at entry was associated with IGF-1 responses to training only in women, and not in men. IGF-1 was associated with body composition and fitness improvements in men, but not women.
O’Leary et al., 2023 †,‡ [59] RQ: 87.9%	Military Design: Prospective cohort (36 h); field exercise in energy deficit as part of Commissioning Course at the Royal Military Academy (Sandhurst, UK) Primary outcomes: Bone turnover, diet, energy expenditure	14 F British Army Officer Cadets 23 ± 1 y, 61.6 ± 6.6 kg, LM = 45.3 ± 5.4 kg, FM = 14.2 ± 2.4 kg	Hormones: Cortisol, testosterone Methods: Plasma Fasted status: Overnight	Cortisol, nmol/L: Baseline: 650.8 ± 229.9 Post: 578.5 ± 219.5 Recovery: 606.9 ± 165.3 *p* > 0.05 (time) Testosterone, nmol/L: Baseline: 1.4 ± 1.2 Post: 0.8 ± 0.5 Recovery: 0.8 ± 0.3 *p* > 0.05 (time)	Bone turnover: βCTX, PINP, parathyroid hormone, total 25(OH)D, albumin-adjusted calcium, total 1,25(OH)2D, phosphate, total 24,25(OH)2D Diet: carbohydrate, protein, and fat intake Energetics: energy expenditure and balance (accelerometry and doubly labeled water)	Testosterone went unchanged throughout training for women and changed dramatically for men. Cortisol was unchanged between time points for both men and women.
O’Leary et al., 2024 ‡ [60] RQ: 66.7%	Military Design: Prospective cohort (44 wk); Commissioning Course (basic combat training program) at the Royal Military Academy (Sandhurst, UK) Primary outcomes: Energy balance, bone turnover, metabolic and endocrine statuses	23 (9 M, 14 F) British Army Officer Cadets M: 25 ± 3 y, 85.3 ± 7.2 kg F: 24 ± 2 y, 66.4 ± 6.2 kg	Hormones: Cortisol, IGF-1, testosterone Methods: Plasma (cortisol, testosterone) and serum (IGF-1) Fasted status: Overnight	Cortisol, nmoll/L (F) * Baseline: 776.3 ± 174.6 Term 2: 724.3 ± 226.6 Term 3: 733.6 ± 202.4 IGF-1, nmmol/L (F) Baseline: 215.5 ± 52.5 Term 2: 230.4 ± 65.2 Term 3: 233.7 ± 52.7 Testosterone, nmoll/L (F) * Baseline: 0.7 ± 0.2 Term 2: 0.7 ± 0.3 Term 3: 1.2 ± 1.5 * *p* < 0.05 (time)	Body composition: LM, FM, BF Energetics: energy intake, energy balance, energy expenditure, macronutrient intake Bone turnover: Bone alkaline phosphatase, βCTX, PINP Metabolic and endocrine statuses: Leptin, triiodothyronine, free thyroxine, thyroid-stimulating hormone, sex hormone-binding globulin, free androgen index	Cortisol decreased significantly between terms 1 and 3. IGF-1 remained unchanged across timepoints for women, while testosterone only increased slightly during term 3.
Strahler et al., 2015 ‡ [65] RQ: 65.6%	Police Design: Cross-sectional; simulated school shooting exercise as part of basic or refresher training session Primary outcomes: Psychobiological stress	50 police officers (21 M, 9 F) within the German police force M: 39.9 ± 8.7 y, 26.4 ± 3.1 kg/m^2^, 16.4 ± 8.0 y experience F: 37.4 ± 9.1 y, 23.0 ± 2.0 kg/m^2^, 17.2 ± 9.4 y experience	Hormones: α-amylase Methods: Saliva Fasted status: NR	α-amylase, U/mL (F): Basal: 125.8 (26.6) +1 min: 259.2 (69.2) +20 min: 199.7 (40.4) +40 min: 218.2 (32.9) *p*-value NR	Psychological state: Chronic and acute stress, mood Physiological stress: Cortisol, HR, HR variability (only α-amylase data were disaggregated by sex)	Female officer reports greater strain and anxiety during the simulated school shooting. Salivary α-amylase was significantly increased immediately post-simulation and was highest in officers in the front of the formation (12:00 position). Salivary α-amylase remained elevated during recovery in female officers. Cortisol was highest at the start of the simulation and progressively decreased throughout.
Szivak et al., 2018 †,# [22] RQ: 65.6%	Military Design: Prospective cohort (2 wk); U.S. Navy SERE training (Kittery and Rangeley, ME, USA) Primary outcomes: Neuroendocrine markers and performance	24 Marines (20 M, 4 F) Men were separated into high and low fit groups (n = 10 for each); women were not included in the final analysis High-fit M: 25.3 ± 4.4 y, 82.2 ± 17.9 kg Low-fit M: 25.2 ± 9.0 y, 85.2 ± 30.4 kg F: 22.3 ± 2.5 y, 67.2 ± 5.1 kg	Hormones: Epinephrine, norepinephrine, dopamine, cortisol, testosterone, NPY Methods: Serum (cortisol, testosterone), plasma (NPY, epinephrine, norepinephrine, dopamine) Fasted status: Yes (time-period not specified)	Cortisol, nmol/L (F): Baseline: 139.8 ± 60.6 Stress: 937.4 ± 276.4 Recovery: 251.1 ± 60.5 Testosterone, nmol/L (F): Baseline: 1.1 ± 0.2 Stress: 1.8 ± 0.3 Recovery: 1.0 ± 0.2 NPY, pg/mL (F): Baseline: 356.7 ± 53.5 Stress: 317.3 ± 92.2 Recovery: 174.3 ± 26.6 Epinephrine, pmol/L (F): Baseline: 234.7 ± 88.8 Stress: 361.6 ± 155.5 Recovery: 182.8 ± 82.3 Norepinephrine, pmol/L (F): Baseline: 2291.5 ± 360.0 Stress: 6511.0 ± 2089.6 Recovery: 3855.5 ± 1267.4 Dopamine, pmol/L (F): Baseline: 87.0 ± 13.3 Stress: 169.6 ± 36.0 Recovery: 133.7 ± 30.8 *p*-values: NR for all outcomes	Physical performance: Dominant handgrip strength, vertical jump height	SERE training appears to induce increases in all blood biomarkers except NPY. Similar trends were observed for male participants. Women were not included in the final analysis; therefore, future studies investigating stress responses to SERE training should include female participants whenever possible.
Tomei et al., 2008 [67] RQ: 34.4%	Police Design: Cross-sectional; urban stressor exposure (Rome, Italy) Primary outcomes: Testosterone	192 police officers (96 traffic officers and 96 controls) Officers: 38.7 ± 4.3 y, 7.4 ± 5.2 y experience Controls: 39.8 ± 4.0 y, 6.6 ± 4.7 y experience	Hormones: Free testosterone Methods: Plasma Fasted status: Overnight	Free testosterone, pg/mL: Baseline: 1.4 ± 0.6 *p* < 0.001 (control)	None	Testosterone concentrations were higher in female traffic officers compared with controls. Differences in testosterone concentrations may be due to chronic work-related exposure to mild environmental urban stressors.
Vikmoen et al., 2020 † [68] RQ: 63.6%	Military Design: Prospective cohort (14 days) during a 6-day field-based Selection Exercise at Rena Military Camp (Rena, Norway) Primary outcomes: Body composition and performance	35 conscripts recruited from the Parachute Ranger Platoon (23 M) and the Special Reconnaissance Platoon (12 F) M: 19.3 ± 1.8 y, 79.5 ± 6.3 kg F: 19.4 ± 1.5 y, 67.7 ± 5.5 kg	Hormones: Cortisol, IGF-1, testosterone Methods: Serum Fasted status: Overnight	Cortisol, ug/dL (F): Pre: 343 ± 219 Post 24 h: 771 ± 155 * Post 72 h: 677 ± 196 * Post 1 wk: 666 ± 101 * Post 2 wk: 711 ± 82 * IGF-1, nmol/L (F): Pre: 17.6 ± 5.1 Post 24 h: 10.1 ± 2.6 * Post 72 h: 13.7 ± 4.3 * Post 1 wk: 23.7 ± 6.9 * Post 2 wk: 26.8 ± 7.9 * Testosterone, nmol/L (F): Pre: 1.0 ± 0.5 Post 24 h: 1.2 ± 0.4 Post 72 h: 1.1 ± 0.4 Post 1 wk: 1.1 ± 0.3 Post 2 wk: 1.0 ± 0.3 * *p* < 0.05 (time, vs. pre)	Body composition: BM, MM, FM Performance: CMJ height and maximal power, medicine ball throw, anaerobic performance (Evacuation test) Other: Creatine kinase	Decreased IGF-1 and increased cortisol were similar between sexes. Cortisol progressively declined during the recovery period for men, but not women. More prominent increases in serum cortisol in women may be due to lower concentrations at baseline.

^a^ Summarized as mean ± standard deviation (SD), mean (standard error), or median [interquartile range; IQR]. ACTH, Adrenocorticotropic hormone. AM, Morning. βCTX, Beta-C telopeptide cross-links of type I collagen. BF, Body fat. BM, Body mass. CCP, Combined contraceptive pill. CMJ, Countermovement jump. CRH, Corticotropin-releasing hormone. DBP, Diastolic blood pressure. F, Female. FM, Fat mass. HCC, Hair cortisol concentration. HDL, High-density lipoprotein. HPA, Hypothalamic–pituitary–adrenal. HPG, Hypothalamic–pituitary–gonadal. HR, Heart rate. IGF-1, Insulin-like growth factor 1. LDL, Low-density lipoprotein. LM, Lean mass. Ln, Natural logarithm. M, Male. MM, Muscle mass. NPY, Neuropeptide Y. NR, Not reported. PINP, Procollagen I N-terminal peptide. PM, Evening. POMS, Profile of mood states. RQ, Reporting quality. SBP, Systolic blood pressure. SERE, Survival, Evasion, Resistance, and Escape. Wk, Week. ‡ Data were extracted via WebPlotDigitizer v5 (https://automeris.io/) for the following studies: Conkright et al., 2021 [24], Gifford et al., 2019 [44], Gifford et al., 2025 [46], McFadden et al., 2024a [54], O’Leary et al., 2023 [59], O’Leary et al., 2024 [60], Strahler et al., 2015 [65]. # Identified via hand-searching (not with an electronic database search). † Study includes a recovery period.

**Table 3:** Summary of the studies included in the scoping review that evaluated body composition.

Author, Year	Tactical Domain and Study Characteristics	Sample Characteristics	Body Composition Assessment and Outcomes		Other Markers Analyzed	Impact of Stress and Sex on Body Composition
			Outcomes and Assessment Details	Aggregate-Level Study Data (Mean ± SD) ^a^		
Ahmed et al., 2020 [32] RQ: 53.1%	Military Design: Prospective cohort (5 days); artic-like field training exercise at the Canadian Forces Base (Meaford, Ontario, Canada) Primary outcomes: Energy intake and expenditure	10 Class A Reservists (6 M, 4 F) M: 29 ± 5 y, 81.9 ± 8.5 kg, 24.9 ± 2.7 kg/m^2^, FM = 19.3 ± 5.3 kg, FFM = 62.6 ± 7.0 kg, BF = 29.2 ± 7.9% F: 34 ± 9 y, 81.8 ± 11.7 kg, 29.0 ± 4.5 kg/m^2^, FM = 27.9 ± 6.4 kg, FFM = 53.9 ± 5.2 kg, BF = 31.4 ± 6.1%	Total: BMI (clothed), BM (clothed), FM, FFM, BF Methods: Deuterium isotope dilution (0.12 g ^2^H per est. kg TBW) Hydration status: NR Fasted status: NR	BMI (F): Pre: 29.0 ± 4.5 kg/m^2^ Post: 28.3 ± 4.4 kg/m^2^ * Total BM (F): Pre: 81.8 ± 11.7 kg Post: 80.1 ± 11.0 kg * Total FM (F): Pre: 27.9 ± 6.4 kg Post: 24.9 ± 7.9 kg * Total FFM (F): Pre: 53.9 ± 5.2 kg Post: 55.3 ± 3.2 kg * Total BF (F): Pre: 31.4 ± 6.1% Post: 27.4 ± 8.2% * * *p* < 0.05 (time)	Energetics: energy expenditure, energy intake, energy deficit, energy availability	Energy deficits of 2540 kcal/day (2377 to 4917 kcal/day) were observed during winter weather field training exercise. Individual meal pack and/or light meal combat were sufficient to meet energy requirements, but adequate intake did not occur (voluntary anorexia). Low energy and nutrient intakes coupled with high energy expenditure resulted in a significant loss (2.7%) of total BM and a 4% decrease in BF% after a 5-day training exercise.
Andrews et al., 2010 † [33] RQ: 51.6%	Military Design: Cross-sectional; service members completing the Army Physical Fitness Test (Washington, DC, USA) Primary outcomes: Oxidative stress	60 overweight or obese active-duty service members (35 M, 25 F) M: 33.1 ± 8.3 y, 99.8 ± 9.9 kg, 31.9 ± 2.8 kg/m^2^ F: 34.4 ± 7.4 y, 82.3 ± 11.0 kg, 29.9 ± 2.3 kg/m^2^	Total: BMI, BM, LM, FM, BF Regional: Trunk FM and trunk BF Methods: DXA (Hologic QDR Discovery Wi, Bedford, MA, USA) Hydration status: NR Fasted status: NR	BMI (F): 29.9 ± 2.3 kg/m^2^ Total BM (F): 82.3 ± 11.0 kg * Total LM (F): 48.4 ± 5.9 kg * Total FM (F): 28.7 ± 4.0 kg Total BF (F): 36.0 ± 3.7% * Trunk FM (F): 13.1 ± 2.8 kg Trunk BF (F): 35.4 ± 5.2 kg * * *p* < 0.05 (sex)	Baseline: Fitness level, dietary intake Oxidative stress: Creatine kinase, C-reactive protein, glutathione peroxidase, superoxide dismutase	The Army Physical Fitness Test causes oxidative stress in overweight soldiers. Body composition appears to influence the degree of oxidative stress incurred by soldiers undergoing this exercise.
Beckner et al., 2023 [34] RQ: 84.4%	Military Design: Prospective cohort (17 days); Cadet Leader Development Training at the U.S. Military Academy (West Point, NY, USA) Primary outcomes: Body composition, performance, energy expenditure, endocrine and metabolic status, metabolomics	72 Cadets (54 M, 18 F) M: 21.7 ± 1.4 y, 84.7 ± 11.1 kg, 26.7 ± 3.1 kg/m^2^, FM = 12.2 ± 5.6 kg, dry LM = 19.5 ± 2.3 kg, BF = 14.2 ± 5.1% F: 21.4 ± 1.2 y, 71.6 ± 10.6 kg, 26.2 ± 3.8 kg/m^2^, FM = 18.6 ± 7.6 kg, dry LM = 14.2 ± 1.3 kg, BF = 25.3 ± 7.0%	Total: BM, dry LM, FM, TBW Methods: BIA (InBody 770, Cerritos, CA, USA) Hydration status: NR Fasted status: NR	Total BM: Pre: 71.2 ± 10.6 kg * Post: 68.6 ± 10.7 kg ** Total dry LM: Pre: 14.2 ± 1.3 kg * Post: 14.2 ± 1.4 kg Total FM: Pre: 18.6 ± 7.6 kg * Post: 15.7 ± 7.3 kg Total TBW: Pre: 38.5 ± 3.3 kg Post: 38.7 ± 3.9 kg * *p* ≤ 0.05 (sex) ** *p* ≤ 0.05 (sex, post vs. pre change)	Energetics: total daily energy expenditure (doubly labeled water) Endocrine status: estradiol, progesterone, total testosterone, free testosterone Metabolic status: serum glycerol, free fatty acids, serum leptin Metabolomics: all metabolites within the lipid super pathway Performance: lower body power	Independent of sex, changes in metabolites related to lipid metabolism were inversely associated with changes in BM and positively associated with changes in endocrine and metabolic status. Women preferentially mobilize fat stores vs. men in response to sustained, physically demanding military training, as evidenced by increased lipid metabolites and enhanced fat oxidation. This may be beneficial for mitigating loss of LM and lower body power.
Charles et al., 2008 [35] RQ: 75.0%	Police Design: Cross-sectional; Buffalo Cardio-metabolic Occupational Police Stress Study (Buffalo Police Department, Buffalo, NY, USA) Primary outcomes: Adiposity and oxidative stress	110 incumbent police officers (67 M, 43 F) 39.6 ± 7.6 y, 28.0 ± 4.4 kg/m^2^, 13.1 ± 8.9 y experience	Total: BMI, WC, waist-to-hip ratio, waist-to-height ratio, abdominal height Methods: Digital scale (clothed, without shoes), tape measure after exhale (nearest 0.5 cm) Hydration status: NR Fasted status: 12-h (for blood collection)	BMI (F): 26.3 ± 4.6 kg/m^2^ * WC (F): 80.4 ± 10.2 cm * Waist-to-hip ratio (F): 0.77 ± 0.06 * Waist-to-height ratio (F): 0.48 ± 0.06 * Abdominal height (F): 19.0 ± 3.0 cm * * *p* < 0.001 (sex)	Oxidative stress: Oxidative stress score, glutathione, glutathione peroxidase, vitamin C, thiobarbituric acid reactive substances, trolox equivalent antioxidant capacity	Adiposity is associated with oxidative stress and decreased antioxidant defense. Sex modifies the interaction between adiposity and oxidative stress, yet the mechanisms behind this require further investigation. People with higher BMI may experience exaggerated oxidative stress and decreased plasma antioxidant levels during exercise.
Cho et al., 2017 [36] RQ: 42.4%	Military Design: Prospective cohort (8 wk); during 16-wk Officer training course at the Korea Third Military Academy (Yeongcheon, South Korea) Primary outcomes: Reproductive function	40 women cadets 22–28 y, 63.6 ± 7.8 kg, 24.1 ± 2.7 kg/m^2^, WC = 71.5 ± 7.0 cm; regular menstrual cycles	Total: BM, BMI, WC Methods: Tape measurement during minimal respiration (WC) Hydration status: NR Fasted status: Overnight	Total BM: 4 wk: 60.0 ± 6.8 kg 8 wk: 59.3 ± 6.4 kg * BMI: 4 wk: 22.7 ± 2.3 kg/m^2^ 8 wk: 22.4 ± 2.2 kg/m^2^ * WC: 4 wk: 67.0 ± 5.8 cm 8 wk: 67.1 ± 4.6 cm * *p* < 0.05 (time)	Reproductive function: regularity, CRH, cortisol, prolactin, endorphin-β, NPY, leptin, orexin-A, ghrelin, follicle-stimulating hormone, luteinizing hormone, estradiol, thyroid-stimulating hormone, thyroxine	BM and BMI decreased progressively throughout arduous military training WC decreased at the beginning of the training (weeks 1–4), then plateaued. Outcomes assessed did not differ by menstrual cycle regularity (i.e., normal vs. irregular).
Coge et al., 2024 ‡ [37] RQ: 65.6%	Military Design: Prospective cohort (34 wk); recruit basic training (Instituto Superior Técnico Militar of Angola) Primary outcomes: Body composition, fitness, and performance	74 recruits (40 M, 37 F; authors did not report drop-outs or why the total sample differed when disaggregated by sex) 23.1 ± 1.99 y (M), 20.9 ± 1.6 y (F), 69.8 ± 11.0 kg, 25.0 ± 4.0 kg/m^2^	Total: BM, BMI, FM Methods: BIA (OMRON HBF 510, Omron Healthcare, Inc., Hoffman Estates, IL, USA) Hydration status: NR Fasted status: NR	Total BM (F): Pre: 65.5 ± 12.0 kg Post: 63.8 ± 11.4 kg *, ** BMI (F): Pre: 24.9 ± 5.3 kg/m^2^ Post: 24.2 ± 5.0 kg/m^2^ ** FM (F): Pre: 28.7 ± 4.6 kg Post: 27.2 ± 4.4 kg * *p* < 0.05 (sex), ** *p* < 0.01 (time)	Fitness: VO_2max_, sprint performance Performance: CMJ, medicine ball throw, push-ups, curl-ups	Both men and women experienced significant changes in BM during the training program. Men experienced greater reductions in BM than women (M: 3.47%, F: 2.70%, *p* < 0.05). Women experienced a greater reduction in FM than men (M: 3.15%, F: 5.34%, *p* < 0.01).
Conkright et al., 2022 [38] RQ: 68.6%	Military Design: Prospective cohort (5 days); simulated military operational stress protocol with restricted sleep and caloric intake Primary outcomes: Extracellular vesicle biomarkers	20 U.S. service members (10 M, 10 F) M: 25.6 ± 5.8 y F: 27.1 ± 5.9 y	Total: BM, BF Methods: Air displacement plethysmography (BOD POD, Cosmed, Concord, CA, USA) Hydration status: NR Fasted status: Overnight	Total BM (F): 70.8 ± 8.1 kg * Total BF (F): 28.2 ± 6.7% * * *p* < 0.05 (sex)	Performance: Baseline VO_2peak_, average knee extensor maximal voluntary contraction Extracellular vesicle biomarkers: Concentration, size Other: Contraceptive use, sleep, caloric intake, perceived exertion, myoglobin, creatine kinase	Women had lower BM and higher BF compared to men. The severity of caloric and sleep restriction did not differ between sexes, while total sleep and caloric intake were also similar between sexes. No extracellular vesicle subpopulation concentration or size was different between men and women.
Cuddy et al., 2015 [39] RQ: 27.3%	Fire Design: Prospective cohort (3 days); live wildland fire suppression (Fort Collins, CO, USA) Primary outcomes: Physiological strain, thermal responses, energy expenditure	15 wildland firefighters (12 M, 3 F) from two Type I Interagency Hot Shot fire crews 26 ± 3 y, 78.3 ± 8.6 kg, 24.3 ± 1.7 kg/m^2^, 6 ± 2 y experience	Total: BM Methods: Digital scale Hydration status: NR Fasted status: NR	Total BM (F): Pre: 66.7 ± 4.4 Post: 65.7 ± 4.7 *p*-value NR	Physiological strain: Physiological strain index rating, heart rate Thermal responses: Core and skin (chest) temperature Energetics: Energy expenditure, activity, water turnover	Total energy expenditure during wildland firefighting suggests occupational demands have not deviated from recent trends. In the total sample, there were no significant changes in BM across the 3-day period; sex-specific outcomes were NR. Wildland firefighters sustain relatively high chest skin temperature throughout their work shift, yet they appear to modulate work activity to avoid excessive cardiovascular and thermal strain.
Dawes et al., 2023 [40] RQ: 64.5%	Police Design: Retrospective cohort; archived health and fitness records from officers with ≥5 y experience Primary outcomes: Body composition and performance	523 state patrol officers (494 M, 29 F) M: 39.0 ± 7.4 y F: 36.5 ± 7.7 y	Total: BM, BMI Methods: Digital scale Hydration status: NR Fasted status: NR	Total BM (F, *n* = 23): Year 1: 76.5 ± 14.9 kg Year 5: 79.1 ± 15.9 kg *p* = 0.106 (time) BMI (F, *n* = 24): Year 1: 26.2 ± 4.3 kg/m^2^ Year 5: 27.1 ± 4.4 kg/m^2^ *p* = 0.105 (time)	Performance: Vertical jump height, sit-ups, push-ups Fitness: VO_2max_	This study suggests minimal differences in BM and BMI occur in response to 5 y of service in law enforcement.
Dicks et al., 2023 [41] RQ: 56.3%	Police Design: Cross-sectional; physical readiness assessment (Midwestern Police Department) Primary outcomes: Physical Readiness Assessment performance and body composition	30 incumbent police officers (25 M, 5 F) 33.9 ± 8.3 y, 9.2 ± 8.6 y experience	Total: BM, BMI, BF, FFM Methods: BIA (Tanita, TBF-300A, Tokyo, Japan) Hydration status: NR Fasted status: NR	Total BM (F): 73.3 ± 12.2 kg * BMI (F): 26.6 ± 2.5 kg/m^2^ Total BF (F): 33.7 ± 5.0% Total FFM (F): 48.2 ± 5.6 kg ** ** *p* < 0.001 (sex), * *p* < 0.05 (sex)	Performance: Handgrip strength, physical activity rating, moderate-to-vigorous physical activity, time to complete the physical readiness assessment	A higher BF percentage is associated with a longer time to complete the Physical Activity Readiness Assessment.
Evans et al., 2008 [42] RQ: 60.6%	Military Design: Prospective cohort (~4 months); Israeli Defense Force gender-integrated basic recruit training program (Tel Hashomer, Israel) Primary outcomes: Body composition, fitness, bone turnover, endocrine regulation, inflammation	194 (41 M, 153 F) recruits from three training cohorts (November–March, April–August, December–April) M: 19.3 ± 1.2 y, 70.0 ± 14.4 kg, 22.4 ± 3.7 kg/m^2^, BF = 17.5 ± 5.4% F: 19.0 ± 1.0 y, 60.9 ± 10.2 kg, 23.2 ± 3.3 kg/m^2^, BF = 30.7 ± 4.9%	Total: LM, FM, BF Methods: four-site skinfolds (BF); weight multiplied by BF (FM); FM subtracted from weight (LM) Hydration status: NR Fasted status: Overnight	Total LM (F): Pre: 41.9 ± 5.3 kg Post: 43.8 ± 5.0 kg * Total FM (F): Pre: 19.0 ± 5.8 kg Post: 18.3 ± 5.4 kg * Total BF (F): Pre: 30.7 ± 4.9% Post: 29.0 ± 4.5% * *p* < 0.002 (time)	Fitness: VO_2max_, 2-km run time Bone turnover: Bone alkaline phosphatase, PINP, tartrate-resistant acid phosphatase, C-telopeptide cross-links of type I collagen Endocrine regulation: Albumin, calcium, PTH Inflammation: TNF-α, IL-1b, IL-6	Bone turnover markers increase similarly for both sexes for the first 2 months of basic combat training. Endocrine regulators are significantly correlated with bone turnover for both sexes. Body composition changes were similar between men and women. Fitness and serum calcium are associated with baseline bone formation markers.
Gifford et al., 2021 [45] RQ: 81.8%	Military Design: Prospective cohort (11 months); Commissioning Course (basic combat training) at the Royal Military Academy (Sandhurst, UK) Primary outcomes: Reproductive and metabolic function Part of the Female Endocrinology in Arduous Training (FEAT) Study	47 women recruits; healthy and naïve to military life 24.1 ± 2.6 y, 64.1 ± 7.1 kg, 23.3 ± 2.1 kg/m^2^, FM = 15.6 ± 3.8 kg, FFM = 48.5 ± 5.3 kg Women grouped by contraception use: None (*n* = 18), combined oral contraceptive pill (*n* = 13), progestogen-only contraception (*n* = 16)	Total: BM, FM, FFM, VAT Regional: FM and FFM for arms, legs, trunk, gynoid, android Methods: DXA (GE Lunar iDXA, GE Healthcare, Madison, WI, USA) Hydration status: NR Fasted status: 12-h	Total BM: 14 wk: 63.3 ± 7.2 kg 29 wk: 64.7 ± 6.8 kg 43 wk: 64.3 ± 6.9 kg * Total FM: 14 wk: 14.5 ± 3.4 kg 29 wk: 16.2 ± 3.2 kg 43 wk: 15.6 ± 3.3 kg *** Total FFM: 14 wk: 49.1 ± 5.1 kg 29 wk: 48.5 ± 4.9 kg 43 wk: 48.7 ± 4.9 kg * VAT: 14 wk: 95.4 ± 72.5 g 29 wk: 132.5 ± 93.4 g 43 wk: 137.2 ± 72.6 g ** Regional FM—arms: 14 wk: 1.7 ± 0.4 kg 29 wk: 1.9 ± 0.4 kg 43 wk: 1.8 ± 0.4 kg ** Regional FM—legs: 14 wk: 6.2 ± 1.4 kg 29 wk: 6.7 ± 1.4 kg 43 wk: 6.5 ± 1.4 kg *** Regional FM—trunk: 14 wk: 5.8 ± 1.8 kg 29 wk: 6.8 ± 1.8 kg 43 wk: 6.4 ± 1.8 kg *** Regional FM—gynoid: 14 wk: 3.0 ± 0.7 kg 29 wk: 3.4 ± 0.7 kg 43 wk: 3.3 ± 0.7 kg *** Regional FM—android: 14 wk: 0.7 ± 0.3 kg 29 wk: 0.9 ± 0.3 kg 43 wk: 0.8 ± 0.3 kg *** Regional FFM—arms: 14 wk: 5.2 ± 0.7 kg 29 wk: 5.3 ± 0.7 kg 43 wk: 5.1 ± 0.7 kg * Regional FFM—legs: 14 wk: 16.8 ± 2.0 kg 29 wk: 61.6 ± 1.9 kg 43 wk: 16.6 ± 1.8 kg Regional FFM—trunk: 14 wk: 23.6 ± 2.5 kg 29 wk: 23.1 ± 2.4 kg 43 wk: 23.5 ± 2.6 kg *** *p* < 0.0001 (time) Regional FFM—gynoid: 14 wk: 7.7 ± 0.9 kg 29 wk: 7.5 ± 0.9 kg 43 wk: 7.6 ± 1.0 kg ** Regional FFM—android: 14 wk: 3.2 ± 0.4 kg 29 wk: 3.2 ± 0.4 kg 43 wk: 3.3 ± 0.4 kg * *** *p* < 0.0001, ** *p* ≤ 0.001, * *p* ≤ 0.02 (time)	Fasting metabolic: Leptin, HOMA2 IR, IGF-1, glucose, nonesterified fatty acids, total triiodothyronine, free thyroxine, thyroid-stimulating hormone Basal reproductive: Luteinizing hormone, follicle-stimulating hormone (and its ratio), gonadotropin-releasing hormone, inhibin B, SHBG, free androgen index, DHEA, androstenedione, progesterone Others: C-peptide, creatinine, estradiol, anti-Müllerian hormone, prolactin, testosterone	HPG axis suppression with anovulation resulted from multistressor military training without evidence of low energy availability. VAT increased, while FM and FFM did not change from baseline. HPG axis suppression may have been exacerbated by various aspects of the training (i.e., sleep disturbances) that upregulate the HPA axis.
Kargl et al., 2024 [49] RQ: 60.6%	Military Design: Prospective cohort (10 wk); U.S. Marine Corps Officer Candidate School Primary outcomes: Inflammation, oxidative stress, stress, sleep, performance	163 Marine recruits (101 M, 62 F) M: 24 ± 3 y, 80.4 ± 8.3 kg, 25.9 ± 2.3 kg/m^2^ F: 24 ± 3 y, 66.3 ± 6.6 kg, 24.2 ± 1.7 kg/m^2^	Total: BM, BMI Methods: Digital scale Hydration status: NR Fasted status: Not fasted	Total BM (F): Wk 0: 66.3 ± 6.6 kg Wk 10: 66.2 ± 6.0 kg *p* > 0.05 (time) *p* < 0.05 (sex) BMI (F): Wk 0: 24.2 ± 1.7 kg/m^2^ Wk 10: 24.2 ± 1.9 kg/m^2^ *p* > 0.05 (time) *p* < 0.05 (sex)	Inflammation: C-reactive protein, IL-6, IL-8, IL-10, TNF-α, interferon-γ Oxidative stress: Peroxidized lipid, protein carbonyls, and antioxidative capacity Sleep: Disturbances (Athlete Sleep Screening Questionnaire) Stress: Perceived Stress Scale Performance: Physical fitness and combat fitness test scores	Officer Candidate School yielded non-significant changes in BM and BMI for women over the course of 10 wks. Officer Candidate School is pro-inflammatory and produces minor improvements in oxidative stress.
Krugly et al., 2023 [50] RQ: 54.5%	Police Design: Retrospective cohort; three semesters of police education in Sweden Primary outcomes: Fitness and mental health	2 recruit cohorts; Group 1: *n* = 1736 (1183 M, 553 F) and Group 2: *n* = 407 (276 M, 129 F, 2 other) Group 1: 26.3 ± 4.9 y Group 2: 28.7 ± 6.9 y Combined: 79.1 ± 12.4 kg, 24.6 ± 2.7 kg/m^2^	Total: BM, BMI Methods: Digital scale Hydration status: NR Fasted status: NR	Total BM (F): Semester 1: 67.8 ± 7.9 kg Semester 3: 68.4 ± 7.9 kg BMI (F): Semester 1: 23.3 ± 2.4 kg/m^2^ Semester 3: 23.5 ± 2.3 kg/m^2^ *p*-values: NR	Fitness: Push-ups, sit-ups, grip strength, VO_2max_, standing long jump, agility (Harres test and L-run test), self-reported physical activity Mental health: Self-reported mental health and perceived police ability	Police education in Sweden may not provide students with adequate means of maintaining their physical fitness. BM and BMI did not appear to differ greatly between semester 1 and semester 3 measurements.
Lieberman et al., 2008 [51] RQ: 66.7%	Military Design: Prospective cohort (13 wk); U.S. Marine Corps basic training (Parris Island, SC, USA) Primary outcomes: Body composition, metabolic status, mood state	50 women recruits 19.7 ± 2.1 y, 63.9 ± 0.8 kg, FM = 19.5 ± 0.6 kg, FFM = 41.7 ± 0.5 kg, BF = 30.2 ± 0.7%	Total: BM, FM, FFM, BF, BMM Methods: DXA (DPX-L, Lunar Radiation Corp) Hydration status: NR Fasted status: Overnight	Total BM: Wk 1: 63.9 ± 0.8 kg Wk 5: 61.8 ± 0.8 kg Wk 8: 61.4 ± 0.8 kg Wk 12: 61.7 ± 0.7 kg * Total FM: Wk 1: 19.5 ± 0.6 kg Wk 5: 16.2 ± 0.6 kg Wk 8: 15.2 ± 0.5 kg Wk 12: 14.7 ± 0.5 kg * Total FFM: Wk 1: 41.7 ± 0.5 kg Wk 5: 42.7 ± 0.5 kg Wk 8: 43.3 ± 0.5 kg Wk 12: 44.1 ± 0.5 kg * Total BF: Wk 1: 30.2 ± 0.7% Wk 5: 26.1 ± 0.7% Wk 8: 24.6 ± 0.7% Wk 12: 23.7 ± 0.7% * Total BMM: Wk 1: 2.8 ± 0.1 kg Wk 5: 2.8 ± 0.1 kg Wk 8: 2.8 ± 0.1 kg Wk 12: 2.9 ± 0.1 kg * *p* < 0.001 (time, vs. wk 1)	Metabolic status: Cholesterol (total, LDL, HDL), free fatty acids, cortisol, glucose Mood state: POMS subscales (fatigue, confusion, depression, tension, anger, vigor)	Both mood state and body composition improved substantially over the course of U.S. Marine Corps recruiting training. U.S. Marine Corps training appears to provoke larger improvements in body composition compared to other branch-specific training programs.
Lieberman et al., 2012 [52] RQ: 60.6%	Military Design: Prospective cohort (12 wk); U.S. Marine Corps basic training (Parris Island, SC, USA) Primary outcomes: Body composition, mood state, metabolic status	35 women recruits 19.3 ± 1.7 y, 23.1 ± 1.8 kg/m^2^	Total: BM, FM, LM, BMM Methods: DXA (model DPX-L, LUNAR Radiation Corp, Madison, WI, USA) Hydration status: NR Fasted status: Overnight	Total BM: Pre: 63.6 ± 5.5 kg Post: 62.1 ± 4.9 kg * Total FM: Pre: 19.0 ± 4.4 kg Post: 14.8 ± 3.4 * Total LM: Pre: 41.7 ± 3.7 kg Post: 44.4 ± 3.9 kg * Total BMM: Pre: 2.9 ± 0.4 kg Post: 3.0 ± 0.4 kg * *p* ≤ 0.001 (time)	Mood state: POMS subscales (fatigue, confusion, depression, tension, anger, vigor) Metabolic status: Substance P, fructosamine, adrenocorticotropic hormone, cholesterol (total, HDL, LDL), triglycerides, free fatty acids, DHEA-S	Women undergo relatively rapid changes in mood state, body composition, and metabolic status over the course of basic U.S. Marine Corps recruit training. A limited number of metabolic status markers predict changes in mood with recruit training.
McClung et al., 2009 [53] RQ: 60.6%	Military Design: Prospective cohort (8 wk); U.S. Army basic combat training course (Fort Jackson, SC, USA) Primary outcomes: Iron status, performance, mood state	219 women soldiers Participants separated into two supplementation groups: Iron (n = 86) vs. Placebo (n = 85) Iron: 20.4 ± 4.2 y Placebo: 20.8 ± 4.4 y	Total: BM Methods: Digital scale Hydration status: NR Fasted status: Overnight	Total BM—Iron: Pre: 61.8 ± 9.4 kg Post: 61.8 ± 8.2 kg Total BM—Placebo: Pre: 62.2 ± 8.5 kg Post: 61.9 ± 6.9 kg *p*-values: NR	Iron status: Hemoglobin, red blood cell distribution width, ferritin, transferrin saturation, soluble transferrin receptor Performance: 2-mile run time Mood state: POMS subscales (fatigue, confusion, depression, tension, anger, vigor)	Iron status does not appear to influence BM changes resulting from basic combat training. Female recruits should be screened for iron status upon entry to basic combat training.
McFadden et al., 2024b [55] RQ: 72.7%	Military Design: Prospective cohort (11 wk); U.S. Marine Corps basic training (Parris Island, SC, USA) Primary outcomes: Performance, resilience, wearable tracking Part of a larger study, the U.S. Marine Corps Gender-Integrated Recruit Training study	196 recruits (97 M, 99 F) Baseline characteristics NR	Total: BM Methods: Digital scale Hydration status: NR Fasted status: NR	Total BM (F): Wk 2: 62 ± 8 kg Wk 11: 61 ± 7 kg *p*-value NR	Performance: Physical and combat fitness tests, lower body strength and power Resilience: Connor–Davidson Resilience Scale, workload, self-reported sleep, stress Wearable tracking: energy expenditure, distances, sleep, acceleration Other: Salivary cortisol	Increases in BM during recruit training were associated with increased strength and power, and with lower physical fitness test scores. There is a need to balance aerobic conditioning with strength and power training in tactical training if military readiness is to be prioritized.
Nindl et al., 2012 [56] RQ: 57.6%	Military Design: Prospective cohort (~4 months); Israeli Defense Force gender-integrated basic recruit training program (Tel Hashomer, Israel) Primary outcomes: Body composition, inflammation, fitness	194 (29 M, 93 F) recruits M: 19.1 ± 1.3 y, 72.6 ± 2.7 kg, VO2max = 51.6 ± 1.1 mL/kg/min F: 18.8 ± 0.6 y, 61.6 ± 0.6 kg, VO_2max_ = 36.8 ± 0.7 mL/kg/min Collected in conjunction with Evans et al., 2008 [42]	Total: BM, FM, FFM, BF Methods: Digital scale, four-site skinfolds (biceps, triceps, suprailiac, subscapular) Hydration status: NR Fasted status: Overnight (for blood collection)	Total BM (F): Pre: 61.6 (1.1) kg Post: 62.7 (1.1) kg * Total FM (F): Pre: 19.6 (0.6) kg Post: 19.0 (0.6) kg *^,^** Total FFM (F): Pre: 42.0 (0.6) kg Post: 43.7 (0.5) kg *^,^** Total BF (F): Pre: 31.3 (0.5)% Post: 29.7 (0.5)% *^,^** * *p* < 0.05 (sex), ** *p* < 0.05 (time)	Inflammation: IL-1β, IL-6, TNF-α, IGF-1, free IGF-1, IGF binding proteins-1, -2, -3, -4, -5, and -6 Fitness: VO_2max_	Gender-integrated military recruit training promotes body composition changes in both men and women. Both women and men experience exaggerated inflammation resulting from prolonged basic military training.
Øfsteng et al., 2020 † [57] RQ: 63.6%	Military Design: Prospective cohort (17 days); 10-day military field exercise followed by 7 days of recovery Primary outcomes: Body composition and performance	38 2nd year soldiers (31 M, 7 F) at the Norwegian Defense Cyber Academy Participants (21.6 ± 0.8 y) divided into groups by HIGH and LOW protein intake: LOW: 76.2 ± 12.2 kg HIGH: 75.9 ± 12.2 kg	Total: BM, FM, FFM Methods: DXA (Lunar Prodigy densitometer, Prodigy Advance PA + 302 047, Lunar) Hydration status: NR Fasted status: Overnight	Total BM (F): Data NR *p* = 0.02 (sex) *p* = 0.17 (sex × time) Total FM (F): Data NR *p* = 0.58 (sex) *p* = 0.64 (sex × time) Total FFM (F): Data NR *p* < 0.001 (sex) *p* = 0.15 (sex × time)	Performance: Lower body strength and power, upper body strength, anaerobic power Energetics: Energy expenditure Sleep: Quantity Other: Testosterone, free testosterone, SHBG, IGF-1, cortisol, triiodothyronine, thyroxine, thyroid-stimulating hormone, creatine kinase, testosterone/cortisol ratio	While men displayed higher BM and FFM than women, there was no effect of sex on loss of BM, FM, or FFM over the course of the training exercise. Increased protein intake did not mitigate body composition changes resulting from strenuous 10-day training.
O’Leary et al., 2023 † [59] RQ: 75.8%	Military Design: Prospective cohort (36 h); field exercise in energy deficit as part of Commissioning Course at the Royal Military Academy (Sandhurst, UK) Primary outcomes: Bone turnover, diet, energy expenditure	14 female British Army Officer Cadets 23 ± 1 y, 61.6 ± 6.6 kg, LM = 45.3 ± 5.4 kg, FM = 14.2 ± 2.4 kg	Total: BM Methods: Digital scale Hydration status: NR Fasted status: Overnight	Total BM: Baseline: 61.6 ± 6.6 kg Exercise: 60.8 ± 7.2 kg Recovery: 61.5 ± 7.2 kg *p* > 0.05 (time) *p* < 0.05 (sex)	Bone turnover: βCTX, PINP, PTH, total 25(OH)D, albumin-adjusted calcium, total 1,25(OH)2D, phosphate, total 24,25(OH)2D Diet: carbohydrate, protein, and fat intake Energetics: energy expenditure and balance (accelerometry and doubly labeled water) Other: Testosterone, cortisol	Men and women experience similar changes in bone metabolism over the course of the Royal Military Academy Commissioning Course. The mechanism behind decreased bone formation remains unclear, yet it may be due to low energy availability and its effect on regulators of bone metabolism.
O’Leary et al., 2024 ‡ [60] RQ: 66.7%	Military Design: Prospective cohort (44 wk); Commissioning Course (basic combat training program) at the Royal Military Academy (Sandhurst, UK) Primary outcomes: Energy balance, body composition, bone turnover, metabolic and endocrine statuses	23 (9 M, 14 F) British Army Officer Cadets M: 25 ± 3 y, 85.3 ± 7.2 kg F: 24 ± 2 y, 66.4 ± 6.2 kg	Total: LM, FM, BF Methods: DXA (Lunar iDXA, GE Healthcare, UK) Hydration status: NR Fasted status: ~10-h	Total LM (F): Baseline: 47.4 ± 3.9 kg * Term 1: 47.0 ± 4.3 kg * Term 2: 46.7 ± 3.9 kg * Term 3: 46.5 ± 3.9 kg * Total FM (F): Baseline: 15.9 ± 4.0 kg Term 1: 14.6 ± 2.9 kg Term 2: 16.17 ± 2.7 kg ** Term 3: 16.6 ± 2.9 kg ** Total BF (F): Baseline: 24.7 ± 4.7% * Term 1: 23.5 ± 3.7% * Term 2: 25.6 ± 3.1% *^,^*** Term 3: 26.2 ± 2.7% *^,^*** * *p* < 0.05 (sex), ** *p* < 0.05 (time, vs. Term 1), *** *p* < 0.05 (time, vs. Baseline and Term 1)	Energetics: energy intake, energy balance, energy expenditure, macronutrient intake Bone turnover: Bone alkaline phosphatase, βCTX, PINP Metabolic and endocrine statuses: Leptin, IGF-1, triiodothyronine, free thyroxine, TSH, testosterone, SHBG, free androgen index, cortisol	LM did not change in response to military training, while FM and BF both increased at each term. Men experienced a greater energy deficit than women due to greater energy expenditure. Athlete models of chronic energy deficiency may not be appropriate for multistressor military environments.
O’Leary et al., 2025 # [61] RQ: 75.8%	Military Design: Prospective cohort (13 wk); basic combat training program (Army Training Centre, Pirbright, UK) Primary outcomes: Body composition, performance, iron status, vitamin D status, bone metabolism markers	450 female British Army recruits Participants separated by oral contraceptive pill use: Non-users (*n* = 182), Combined (*n* = 124), Progestin-only (*n* = 144) Non-users: 22.9 ± 3.7 y, 64.8 ± 8.0 kg, 23.8 ± 2.4 kg/m^2^ Combined: 22.3 ± 3.3 y, 65.8 ± 8.1 kg, 23.9 ± 2.3 kg/m^2^ Progestin-only: 21.5 ± 3.1 y, 63.6 ± 8.3 kg, 23.4 ± 2.5 kg/m^2^	Total: FM, LM, full-body aBMD Regional: aBMD for trunk, arms, and legs Methods: DXA (Lunar iDXA; GE Healthcare, Buckinghamshire, UK) Hydration status: NR Fasted status: Overnight fast not possible for all participants	Total FM, kg * Non-users Δ: −2.1 (−2.6, −1.5) Combined Δ: −2.2 (−3.0, −1.4) Progestin Δ: −2.0 (−2.6, −1.5) Total LM, kg * Non-users Δ: 2.1 (1.8, 2.5) Combined Δ: 2.1 (1.7, 2.6) Progestin Δ: 2.4 (2.1, 2.8) Full-body aBMD, mg/cm^2^ *^,^** Non-users Δ: 0.01 (0.01, 0.01) Combined Δ: 0.01 (−0.00, 0.02) Progestin Δ: 0.01 (−0.00, 0.01) Trunk aBMD, mg/cm^2^ ** Non-users Δ: 0.00 (−0.01, 0.00) Combined Δ: 0.00 (−0.01, 0.00) Progestin Δ: 0.00 (−0.01, 0.01) Arms aBMD, mg/cm^2^ * Non-users Δ: 0.01 (0.00, 0.02) Combined Δ: 0.01 (0.00, 0.03) Progestin Δ: 0.01 (0.00, 0.03) *p* < 0.001 (time) Δ Leg aBMD, mg/cm^2^ *^,^** Non-users Δ: 0.02 (0.01, 0.02) Combined Δ: 0.02 (0.01, 0.03) Progestin Δ: 0.01 (0.01, 0.02) * *p* < 0.001 (time), ** *p ≤* 0.03 (group), group × time: all *p* > 0.05	Performance: 2.4-km run time, maximal lift strength, peak power output Iron status: Ferritin, hemoglobin Vitamin D status: Total 25(OH)D Bone metabolism markers: PTH, βCTX, PINP	Basic combat training increased LM, full-body aBMD, arm and leg aBMD, and decreased FM. Progestin-only oral contraceptive pill use was associated with decreased aBMD and increases in markers of bone metabolism.
Pasiakos et al., 2012 [62]; Margolis et al., 2012 [63] RQ: 63.6%	Military Design: Prospective cohort (10 wk); basic combat training course (Fort Jackson, SC, USA) Primary outcomes: Body composition, cardiometabolic risk, diet, lifestyle factors	209 (118 M, 91 F) U.S. Army recruits M: 21 (19–25) y, 27.0 ± 4.3 kg/m^2^, 14.2 ± 4.6% F: 21 (19–24) y, 25.0 ± 2.9 kg/m^2^, 26.7 ± 5.8%	Total: BM, BF, FFM, BF by BMI^2^ Methods: three-site skinfolds (chest, triceps, subscapular sites for men, triceps, suprailiac, abdominal sites for women) Hydration status: NR Fasted status: Overnight	Total BM (F): Wk 0: 66.3 ± 8.3 kg Wk 3: 66.2 ± 7.8 kg Wk 6: 66.8 ± 7.6 kg * (vs. wk 3) Wk 9: 66.4 ± 7.4 kg Total BF (F): Wk 0: 26.6 ± 5.6% Wk 9: 22.8 ± 5.1% * Total FFM (F): Wk 0: 48.2 ± 4.8 kg Wk 9: 51.0 ± 5.3 kg * Total BF by BMI^2^ (F; <30 kg/m^2^): Wk 0: 26.3 ± 5.4 kg/m^2^ Wk 9: 22.9 ± 5.0 kg/m^2^ Total BF by BMI^2^ (F; ≥30 kg/m^2^): Wk 0: 35.9 ± 3.9 kg/m^2^ Wk 9: *n* = 0 * *p* < 0.05 (time)	Cardiometabolic risk: Glucose, cholesterol (total, LDL, HDL), triglycerides Diet: Total fat, saturated fat, cholesterol, sodium, fiber, fruits, and vegetables Lifestyle factors: Family medical history (myocardial infarction, stroke, diabetes), smoking, sedentary behavior	This study demonstrated the prevalence of health behaviors and biomarkers associated with elevated cardiometabolic risk in U.S. Army recruits. Military training may be effective for reducing cardiometabolic risk via improvements in lipid profiles and glycemic control. BM declined in men but not women, while BF decreased in both men and women.
Popp et al., 2024 [64] RQ: 78.8%	Military Design: Prospective cohort (10 wk); basic combat training course (Fort Jackson, SC, USA) Primary outcomes: Body composition and reproductive function	55 women U.S. Army recruits 22 (22, 23) y, 61.9 (59.6, 64.2) kg, 23.9 (23.1, 24.7) kg/m^2^, FFM = 40.8 (39.3, 42.2) kg, FM = 19.1 (17.8, 20.5) kg, BF = 31.7 (30.1, 33.2)%; Non-hormonal contraceptive-using	Total: BM, BMI, FFM, FM, BF Methods: DXA (Lunar Prodigy, GE Healthcare, Madison, WI, USA) Hydration status: NR Fasted status: Overnight	Δ Total BM, kg: 1.1 (0.3, 1.9) * Δ BMI, kg/m^2^: 0.2 (−0.1, 0.6) Δ Total FFM, kg: 3.1 (2.7, 3.5) ** Δ Total FM, kg: −1.7 (−2.4, −1.0) ** Δ Total BF, %: −3.3 (−4.0, −2.6) ** *p* < 0.001, * *p* < 0.02 (time)	Reproductive function: Leptin, free triiodothyronine, triiodothyronine, free thyroxine, thyroxine, IGF-1 Other: Cortisol	HPO axis suppression with no evidence of luteal activity is evident in most women undergoing basic combat training, including women who report normal menstrual cycles. On average, women gained BM and LM and lost FM. Changes in BM and composition appear to be similar across luteal activity groups.
Szivak et al., 2018 †,# [22] RQ: 56.3%	Military Design: Prospective cohort (2 wk); U.S. Navy SERE training (Kittery and Rangeley, ME, USA) Primary outcomes: Neuroendocrine markers and performance	24 Marines (20 M, 4 F) Men were separated into high and low fit groups (n = 10 for each); women were not included in the final analysis High-fit M: 25.3 ± 4.4 y, 82.2 ± 17.9 kg Low-fit M: 25.2 ± 9.0 y, 85.2 ± 30.4 kg F: 22.3 ± 2.5 y, 67.2 ± 5.1 kg	Total: BM Methods: Digital scale Hydration status: NR Fasted status: Yes (time-period not specified)	Total BM (F): Baseline: 67.2 ± 5.1 kg Stress: 63.5 ± 5.2 kg Recovery: 63.3 ± 5.0 kg *p*-value NR	Physical performance: Dominant handgrip strength, vertical jump height Neuroendocrine markers: Epinephrine, norepinephrine, dopamine, cortisol, testosterone, NPY	BM is likely to decrease for both men and women over the course of SERE training. Women were not included in the final analysis; therefore, future studies investigating stress responses to SERE training should include female participants whenever possible.
Szivak et al., 2023 [66] RQ: 62.5%	Military Design: Cross-sectional; survey of U.S. Military Academy graduates between 1980 and 2011 (West Point, NY, USA) Primary outcomes: Obesity status, activity level, self-reported health status	1342 U.S. Military Academy graduates (701 M, 641 F) M: 47.2 ± 9.3 y, 92.3 ± 14.4 kg, 27.8% >20 y served F: 44.1 ± 9.0 y, 71.7 ± 14.8 kg, 17.2% >20 y served	Total: BMI, BMI category Methods: Self-report from survey Hydration status: NR Fasted status: NR	BMI (F): 25.9 ± 5.5 kg/m^2^ BMI Category (F): Underweight: 0.7% Normal: 53.1% Overweight: 29.6% Obese: 16.6% *p*-values (sex): NR	Activity level: Self-reported; volume per week and activity level rating (low, moderate, high) vs. peers of the same age and sex Self-reported health status: Cardiovascular risk factors, eating behaviors, concerns about appearance, weight, and health	Overweight and obesity prevalence is a concern for U.S. Military Academy graduates, which aligns with that of the greater military population, as well as Veterans and adult civilians.
Vikmoen et al., 2020 † [68] RQ: 66.7%	Military Design: Prospective cohort (14 days) during a 6-day field-based Selection Exercise at Rena Military Camp (Rena, Norway) Primary outcomes: Body composition and performance	35 conscripts recruited from the Parachute Ranger Platoon (23 M) and the Special Reconnaissance Platoon (12 F) M: 19.3 ± 1.8 y, 79.5 ± 6.3 kg F: 19.4 ± 1.5 y, 67.7 ± 5.5 kg	Total: BM, MM, FM Methods: BIA (InBody 720, Biospace Co., Fresno, CA, USA) Hydration status: NR Fasted status: Overnight	Total BM (F): Pre: 67.7 ± 5.5 kg Post 0 h: 65.1 ± 5.4 kg * Post 24 h: 65.8 ± 5.5 kg * Post 72 h: 67.4 ± 6.3 kg Post 1 wk: 68.0 ± 5.7 kg Post 2 wk: 68.1 ± 5.7 kg Total MM (F): Pre: 32.0 ± 1.9 kg Post 0 h: 31.9 ± 2.3 kg Post 24 h: 31.9 ± 2.3 kg Post 72 h: 32.6 ± 2.4 kg Post 1 wk: 32.6 ± 2.1 kg * Post 2 wk: 31.5 ± 2.0 kg Total FM (F): Pre: 10.8 ± 3.7 kg Post 0 h: 8.0 ± 3.3 kg * Post 24 h: 8.5 ± 3.1 kg * Post 72 h: 9.1 ± 3.1 kg * Post 1 wk: 10.2 ± 3.2 kg Post 2 wk: 12.0 ± 3.4 kg * * *p* < 0.05 (time, vs. pre)	Performance: CMJ height and maximal power, medicine ball throw, anaerobic performance Other: IGF-1, cortisol, testosterone, creatine kinase	Men lost more BM and MM than women during field-based military training. Body composition changes were not associated with sex differences observed for changes in strength and anaerobic performance. Decreased IGF-1 and increased cortisol were similar between sexes. Women recovered lower body strength faster than men.
Zurek et al., 2022 [69] RQ: 51.5%	Military Design: Prospective cohort (7 months); command training at the Military University of Land Forces (Wroclaw, Poland) Primary outcomes: Body composition and performance	126 cadets (108 M, 18 F) recruited in two phases (January and July 2021) Demographics NR	Total: BM, BMI, BF, MM Methods: BIA (TANITA, model NR) Hydration status: NR Fasted status: NR	Total BM (F): Phase 1: 63.1 ± 3.8 kg * Phase 2: 62.0 ± 3.5 kg * BMI (F): Phase 1: 22.4 ± 1.2 kg/m^2^ * Phase 2: 22.1 ± 1.3 kg/m^2^ * Total BF (F): Phase 1: 22.4 ± 3.2% * Phase 2: 22.0 ± 3.1% * Total MM (F): Phase 1: 45.0 ± 2.3 kg * Phase 2: 44.9 ± 1.9 kg * * *p* ≤ 0.0006 (sex)	Performance: Handgrip strength, horizontal jump distance, sit-ups, shuttle run, 1000-m run, executive function, shooting performance	Women had high BF and lower BM and MM compared to men, and this was maintained over the course of military training.

^a^ Summarized as mean ± standard deviation (SD), mean (standard error) or Δ (95% confidence interval; CI). aBMD, Areal bone mineral density. βCTX, Beta-C telopeptide cross-links of type I collagen. BIA, Bioelectric impedance analysis. BF, Body fat. BM, Body mass. BMI, Body mass index. BMM, Bone mineral mass. CMJ, Countermovement jump. CRH, Corticotropin-releasing hormone. DHEA, Dihydroepiandrostenedione. DHEA-S, Dehydroepiandrosterone sulfate. DXA, Dual energy X-ray absorptiometry. F, Female. FFM, Fat-free mass. FM, Fat mass. HDL, High-density lipoprotein. HOMA2 IR, Homeostatic modeling assessment of insulin resistance 2. HPA, Hypothalamic–pituitary–adrenal. HPG, Hypothalamic–pituitary–gonadal. HPO, Hypothalamic–pituitary–ovarian. IL, Interleukin. IGF, Insulin-like growth factor. LM, Lean mass. LDL, Low-density lipoprotein. M, Male. MM, Muscle mass. NPY, Neuropeptide Y. PINP, Procollagen I N-terminal peptide. POM, Profile of mood states. PTH, Parathyroid hormone. RQ, Reporting quality. SERE, Survival, Evasion, Resistance, and Escape. SHBG, Sex hormone-binding globulin. TBW, Total body water. TNF-α, Tumor necrosis factor α. VAT, Visceral adipose tissue. VO_2max_, maximal oxygen uptake. WC, Waist circumference. ‡ Data were extracted via WebPlotDigitizer v5 (https://automeris.io/) for the following studies: Coge et al., 2024 [37], O’Leary et al., 2024 [60]. # Identified via hand-searching (not with an electronic database search). † Study includes a recovery period.

**Table 4:** Summary of the studies included in the scoping review that evaluated occupational performance.

Author, **Year**	Tactical Domain and Study Characteristics		Occupational Performance Assessment and Outcomes		Other Markers Analyzed	Impact of Stress and Sex on Performance
		Sample Characteristics	Outcomes and Assessment Details	Aggregate-Level Study Data (Mean ± SD) ^a^		
Andrews et al., 2010 † [33] RQ: 51.6%	Military Design: Cross-sectional; service members completing the Army Physical Fitness Test (Washington, DC, USA) Primary outcomes: Oxidative stress	60 overweight or obese active-duty service members (35 M, 25 F) M: 33.1 ± 8.3 y, 99.8 ± 9.9 kg, 31.9 ± 2.8 kg/m^2^ F: 34.4 ± 7.4 y, 82.3 ± 11.0 kg, 29.9 ± 2.3 kg/m^2^	Fitness: Estimated VO_2max_ METHODS: YMCA submaximal protocol to estimate VO_2max_; tested ≥ 48 h before undergoing the Army Physical Fitness Test	Estimated VO_2max_ (F): 32.5 ± 5.1 mL/kg/min *p* > 0.05 (sex)	Baseline: Body composition, dietary intake Oxidative stress: Creatine kinase, C-reactive protein, glutathione peroxidase, superoxide dismutase	Significant correlations were identified between fitness level and glutathione peroxidase, indicating that fitness level may influence oxidative stress elicited by the Army Physical Fitness Test.
Beckner et al., 2023 [34] RQ: 81.3%	Military Design: Prospective cohort (17 days); Cadet Leader Development Training at the U.S. Military Academy (West Point, NY, USA) Primary outcomes: Body composition, performance, energy expenditure, endocrine and metabolic status, metabolomics	72 Cadets (54 M, 18 F) M: 21.7 ± 1.4 y, 84.7 ± 11.1 kg, 26.7 ± 3.1 kg/m^2^, FM = 12.2 ± 5.6 kg, dry LM = 19.5 ± 2.3 kg, BF = 14.2 ± 5.1% F: 21.4 ± 1.2 y, 71.6 ± 10.6 kg, 26.2 ± 3.8 kg/m^2^, FM = 18.6 ± 7.6 kg, dry LM = 14.2 ± 1.3 kg, BF = 25.3 ± 7.0%	Lower body power: Peak power, average power METHODS: Vertical jump test (assessed in triplicate with ≥1 min rest between each attempt)	Peak power (F): Pre: 3780 [IQR: 499] W Post: 3634 [IQR: 776] W *p*-value NR (time) *p* = 0.085 (sex) Average power (F): Pre: 3788 [IQR: 588] W Post: 3659 [IQR: 8680] W *p*-value NR (time) *p* = 0.079 (sex)	Body composition: BM, dry LM, FM, TBW Energetics: total daily energy expenditure (doubly-labeled water) Endocrine status: estradiol, progesterone, total testosterone, free testosterone Metabolic status: serum glycerol, free fatty acids, serum leptin Metabolomics: all metabolites within the lipid superpathway	Women preferentially mobilize fat stores vs. men in response to sustained, physically demanding military training, which may be beneficial for mitigating loss of LM and lower body power. Changes in lower body power following training did not differ between sexes, but men tended to have greater declines in peak and average lower body power vs. women (∆ [95% CIs]: −244 [−314, −174] vs. −130 [−209, −51] W, *p* = 0.085, *d =* 0.49 and −264 [−321, −208] vs. −169 [−243, −95] W, *p* = 0.079, *d =* 0.50).
Coge et al., 2024 ‡ [37] RQ: 65.6%	Military Design: Prospective cohort (34 wk); recruit basic training (Instituto Superior Técnico Militar of Angola) Primary outcomes: Body composition, fitness, and performance	74 recruits (40 M, 37 F; authors did not report drop-outs or why the total sample differed when disaggregated by sex) 23.1 ± 1.99 y (M), 20.9 ± 1.6 y (F), 69.8 ± 11.0 kg, 25.0 ± 4.0 kg/m^2^	Fitness: VO_2max_, sprint performance Performance: CMJ height, medicine ball throw, push-ups, curl-ups METHODS: Estimated VO_2max_: 20 m shuttle run at a speed dictated by stereo system (initial speed: 8.5 km/h for first 1 min; increased by 0.5 km/h each min), continued until volitional fatigue Sprint performance: Maximal sprinting speed obtained during 2’ 80 m linear sprints CMJ: three attempts with 3 min rest between attempts using Optojump photocell system (Microgate, Bolzano, Italy) Medicine ball throw: three attempts with 3-kg medicine ball (1 min rest between each attempt) Push-ups and curl-ups: Maximum number of attempts in 1 min	VO_2max_ (F): Pre: 33.2 ± 6.5 mL/kg/min Post: 34.1 ± 6.3 mL/kg/min * Sprint performance (F): Pre: 5.4 ± 0.8 m/s Post: 5.8 ± 0.7 m/s * CMJ height (F): Pre: 24.8 ± 7.4 cm Post: 28.5 ± 7.8 cm * Medicine ball throw (F): Pre: 3.9 ± 0.9 m Post: 4.7 ± 0.9 m * Push-ups completed (F): Pre: 21.0 ± 5.6 Post: 23.5 ± 5.1 * Curl-ups completed (F): Pre: 63.6 ± 20.8 Post: 67.7 ± 20.2 * * *p* < 0.01 (time)	Body composition: BM, BMI, FM	Female cadets exhibited low fitness levels upon entering the training program, highlighting the need for pre-training conditioning. Men experienced a greater increase in medicine ball throwing distance than women (M: 11.5%, F: 7.7%, *p* < 0.01). All performance metrics increased significantly over the training period for both men and women.
Conkright et al., 2021 [24] RQ: 57.6%	Military Design: Prospective cohort; 5-day simulated military operational stress protocol (Pittsburgh, PA, USA) Primary outcomes: Neuromuscular performance, mood state, and hormonal responses	69 healthy U.S. service members (54 M, 15 F); 4.3% Air Force, 81.2% Army, 8.7% Marine Corps, 5.8% Reserve Officers’ Training Corps M: 26.4 ± 5.3 y, 85.2 ± 14.0 kg, BF = 20.2 ± 7.1%, VO_2peak_ = 47.8 ± 7.6 mL/kg/min F: 25.6 ± 5.6 y, 67.0 ± 9.0 kg, BF = 27.4 ± 7.2%, VO_2peak_ = 40.5 ± 5.0 mL/kg/min	Lower body power: Jump height (cm), maximum force prior to takeoff (N) Tactical mobility test (see below for test battery) METHODS: Jump height: Vertical jump test (assessed 3x/day: Pre, Mid, Post) Tactical mobility test: Assessed each day (1–4) at ~12:00 h (~90 min); familiarization on Day 0; includes the following: Water can carry, Fire and movement, Casualty drag, 300-m shuttle run (loaded and unloaded), and 4-mile ruck march.	Jump height (F) *** *Day 1*, Pre: 21.0 ± 4.6 cm Mid: 19.2 ± 4.6 cm ** Post: 18.2 ± 5.2 cm ** *Day 2*, Pre: 19.4 ± 4.4 cm Mid: 19.0 ± 4.5 cm ** Post: 19.1 ± 4.4 cm ** *Day 3*, Pre: 19.6 ± 4.5 cm Mid: 18.6 ± 5.1 cm ** Post: 17.9 ± 5.5 cm ** *Day 4*, Pre: 19.3 ± 4.2 cm Mid: 18.9 ± 5.5 cm ** Post: 18.3 ± 5.1 cm ** Maximum force prior to takeoff (F) *** *Day 1*, Pre: 1519 ± 240 N Mid: 1642 ± 228 N Post: 1661 ± 197 N *Day 2*, Pre: 1523 ± 204 N Mid: 1678 ± 255 N Post: 1713 ± 237 N *Day 3*, Pre: 1572 ± 247 N Mid: 1694 ± 240 N Post: 1642 ± 340 N *Day 4*, Pre: 1609 ± 254 N Mid: 1691 ± 228 N Post: 1642 ± 340 N ** Water can carry (F) *** Day 1: 0.98 ± 0.35 m/s Day 2: 1.07 ± 0.42 m/s Day 3: 0.96 ± 0.31 m/s Day 4: 1.00 ± 0.37 m/s Fire and movement (F) Day 1: 152.2 ± 13.3 s * Day 2: 157.4 ± 19.2 s * Day 3: 150.7 ± 17.0 s * Day 4: 145.7 ± 15.2 s * Casualty drag (F) *** Day 1: 82.0 ± 32.2 s Day 2: 72.7 ± 23.1 s Day 3: 67.5 ± 11.0 s Day 4: 67.1 ± 15.3 s 300-m shuttle (F) *** Day 1: 109.1 ± 14.8 s Day 2: 109.8 ± 15.7 s Day 3: 113.3 ± 16.7 s * Day 4: 113.5 ± 17.9 s * 300-m shuttle (loaded) (F) Day 1: 128.9 ± 19.8 s Day 2: 128.9 ± 21.4 s Day 3: 133.5 ± 23.7 s Day 4: 130.3 ± 18.8 s 4-mile ruck march (F) Day 1: 1659.9 ± 149.8 s Day 2: 1599.4 ± 138.6 s Day 3: 1645.8 ± 252.5 s Day 4: 1604.7 ± 146.5 s * *p* ≤ 0.001 (day, vs. day 1), ** *p* ≤ 0.001 (time, vs. pre), *** *p* ≤ 0.02 (main effect, sex)	Hormonal responses: Growth hormone, IGF-1, brain-derived neurotrophic factor., cortisol Mood state: POMS subscales (tension, depression, anger, fatigue, confusion, vigor)	Men and women experience similar changes in tactical mobility following 5 days of simulated military occupational stress. Men performed better than women in events requiring increased strength, power, and speed.
Conkright et al., 2022 [38] RQ: 68.8%	Military Design: Prospective cohort (5 days); simulated military operational stress protocol with restricted sleep and caloric intake Primary outcomes: Extracellular vesicle biomarkers	20 U.S. service members (10 M, 10 F) M: 25.6 ± 5.8 y F: 27.1 ± 5.9 y	Fitness: VO_2peak_ Lower body strength: Knee extensor MVC METHODS: VO_2peak_: Bruce protocol; measured at baseline MVC: 4 3–5 s bilateral isometric knee extensions with ≥1 min between attempts (SSM-AJ-500, Interface Inc., Scottsdale, AZ, USA); measured at baseline	VO_2peak_ (F): 39.0 ± 5.2 mL/kg/min * MVC—knee extensor (F): 900.1 ± 234.5 N * *p* < 0.05 (sex)	Body composition: BM, BF Extracellular vesicle biomarkers: Concentration, size Other: Contraceptive use, sleep, caloric intake, perceived exertion, myoglobin, creatine kinase	Women had lower Fitness and similar knee extensor MVC to men. Caloric and sleep restriction severity did not differ between sexes. Total sleep and caloric intake were also similar between sexes. No extracellular vesicle subpopulation concentration or size was different between sexes.
Dawes et al., 2023 [40] RQ: 64.5%	Police Design: Retrospective cohort; archived health and fitness records from officers with ≥5 y experience Primary outcomes: Body composition and performance	523 state patrol officers (494 M, 29 F) M: 39.0 ± 7.4 y F: 36.5 ± 7.7 y	Lower body power: Vertical jump height Abdominal strength: Sit-ups Muscular endurance: Push-ups Fitness: VO_2max_ METHODS: Vertical jump: Highest recorded of three attempts using an electrical contact-operated system (Just Jump, ProBotics Inc., Huntsville, AL, USA) Sit-ups: Number of completed attempts in 60 s Push-ups: Number of completed attempts in 60 s VO_2max_: 20-m multistage test starting at 8.5 km/h and increasing by 0.5 km/h each stage, standardized by prerecorded audio beeps	Vertical jump height (F, *n* = 21): Year 1: 35.0 ± 8.0 cm Year 5: 34.4 ± 5.4 cm Sit-ups completed (F, *n* = 28): Year 1: 31.4 ± 8.0 Year 5: 35.0 ± 10.8 * Push-ups completed (F, *n* = 23): Year 1: 23.0 ± 11.8 Year 5: 25.9 ± 14.0 VO_2max_ (F, *n* = 29): Year 1: 27.2 ± 5.2 mL/kg/min Year 5: 29.0 ± 5.4 mL/kg/min * *p ≤* 0.001 (time)	Body composition: BM, BMI	Minimal changes in physical fitness and performance occur in response to 5 y of service in law enforcement.
Dicks et al., 2023 [41] RQ: 56.3%	Police; physical readiness assessment Design: Cross-sectional; physical readiness assessment (Midwestern Police Department) Primary outcomes: Physical Readiness Assessment performance and body composition	30 incumbent police officers (25 M, 5 F) 33.9 ± 8.3 y, 9.2 ± 8.6 y experience	Strength: Handgrip PA level: Moderate-to-vigorous PA (min/day), PA rating Fitness: Physical readiness assessment, estimated VO_2max_ METHODS: Handgrip strength: Average of three attempts with 30 s rest between trials Moderate-to-vigorous PA (min/day): Self-report; the International PA Questionnaire—Long Form PA rating: Self-reported on a scale of 0–15 Physical readiness assessment: Time to complete a six-lap mobility run (stair climbing, jump and crawl obstacles, barrier jump, wall vault), followed by simulating the arrest of a resistant subject Estimated VO_2max_: Equation using self-reported PA rating, age in years, BMI, and sex (1 = M, 0 = F)	Handgrip strength (F): 41.0 ± 9.6 kg ** Moderate-to-vigorous PA (F): 30.9 ± 11.5 min/day PA rating (F): 6.2 ± 0.5 * Physical readiness assessment time (F): 304.4 ± 45.0 s Estimated VO_2max_ (F): 37.9 ± 2.2 mL/kg/min * *p* < 0.05 (sex), ** *p* < 0.001 (sex)	Body composition: BM, BMI, BF, FFM	Officers who did not successfully complete the physical readiness assessment (i.e., completion time >4 min 40 s) displayed significantly lower estimated fitness (VO_2max_) than officers who successfully completed the assessment. Officers should focus on improving BF, VO_2max_, and participation in moderate-to-vigorous PA to decrease physical readiness assessment completion times.
Evans et al., 2008 [42] RQ: 60.6%	Military Design: Prospective cohort (~4 months); Israeli Defense Force gender-integrated basic recruit training program (Tel Hashomer, Israel) Primary outcomes: Body composition, fitness, bone turnover, endocrine regulation, inflammation	194 (41 M, 153 F) recruits from three training cohorts (November–March, April–August, December–April) M: 19.3 ± 1.2 y, 70.0 ± 14.4 kg, 22.4 ± 3.7 kg/m^2^, BF = 17.5 ± 5.4% F: 19.0 ± 1.0 y, 60.9 ± 10.2 kg, 23.2 ± 3.3 kg/m^2^, BF = 30.7 ± 4.9%	Fitness: VO_2max_, 2-km run time METHODS: VO_2max_: Continuous graded treadmill protocol (start: 3 min at 3.1 mph with 0% grade, then 2% grade increase every 2 min with unchanging speed [determined by heart rate during warm up]) 2-km run time: Provided by unit to research team	VO_2max_ (F): Pre: 36.7 ± 6.2 mL/kg/min Post: 39.7 ± 5.8 mL/kg/min *p* < 0.002 (time) 2-km run time (F): Pre: 742.5 ± 117.6 s Post: 688.3 ± 90.4 s *p* < 0.002 (time)	Body composition: LM, FM, BF Bone turnover: Bone alkaline phosphatase, PINP, tartrate-resistant acid phosphatase, C-telopeptide cross-links of type I collagen Endocrine regulation: Albumin, calcium, PTH Inflammation: TNF-α, IL-1b, IL-6	Fitness increased in women over the course of gender-integrated basic military training. 2-km run time significantly improved in women undergoing this training. Fitness and serum calcium are associated with baseline bone formation markers.
Greer et al., 2023 [47] RQ: 77.4%	Military Design: Cross-sectional; women seeking care in urogynecology, family medicine, and women’s health clinics between December 2019 and February 2020 Primary outcomes: Psychological stress, impacts of pelvic floor disorders on Naval duties	178 active-duty U.S. Navy service women 30.9 ± 8.8 y, 58.9% received urogynecologic care, 123 met criteria for having ≥ one pelvic floor disorder	Fitness: Physical fitness test failure METHODS: Survey including questions related to demographics, medical history, exercise tolerance, military service, and psychological stress	Physical fitness test failure rate by group: No pelvic floor disorder (*n* = 55): 14.6% ≥1 pelvic floor disorder (*n* = 123): 26.0% *p* = 0.064 (group)	Psychological stress: Perceived Stress Scale score Impact of pelvic floor disorders on Naval duties: Body composition assessment failure, days missed work, deployment, limited duty profile	There was no significant association between pelvic floor disorders and physical fitness test failures. Yet, the percentage of failures was slightly higher in the ≥1 pelvic floor disorder group.
Kargl et al., 2024 [49] RQ: 60.6%	Military Design: Prospective cohort (10 wk); USMC Officer Candidate School Primary outcomes: Inflammation, oxidative stress, stress, sleep, performance	163 Marine recruits (101 M, 62 F) M: 24 ± 3 y, 80.4 ± 8.3 kg, 25.9 ± 2.3 kg/m^2^ F: 24 ± 3 y, 66.3 ± 6.6 kg, 24.2 ± 1.7 kg/m^2^	USMC performance battery: Physical fitness test score, combat fitness test score METHODS: Physical fitness test: Pull-ups or push-ups, plank or sit-ups, time 3-mile run completed during the first and seventh week of Officer Candidate School; scored on a sex- and age-adjusted scale out of 300 Combat fitness test: 880-yard sprint, 30-lb ammunition lift, 300-yard combat-relevant course run completed during the fourth week of Officer Candidate School; scored on a sex- and age-adjusted scale out of 300	Physical fitness test (F): Wk 0: 273.6 ± 14.6 Wk 7: 267.9 ± 19.9 *p* < 0.05 (time) Combat fitness test (F): Wk 4: 281.4 ± 16.7 *p* > 0.05 (sex)	Inflammation: C-reactive protein, IL-6, IL-8, IL-10, TNF-α, IFN-γ Oxidative stress: Peroxidized lipid, protein carbonyls, and antioxidative capacity Sleep: Disturbance (Athlete Sleep Screening Questionnaire) Stress: Perceived stress (Perceived Stress Scale)	Physical fitness test scores decreased in women over the course of the USMC Officer Candidate School, indicating declines in military-specific performance with prolonged arduous training.
Krugly et al., 2023 [50] RQ: 54.5%	Police; police education program Design: Retrospective cohort; three semesters of police education in Sweden Primary outcomes: Fitness and mental health	2 recruit cohorts; Group 1: *n* = 1736 (1183 M, 553 F) and Group 2: *n* = 407 (276 M, 129 F, 2 other) Group 1: 26.29 ± 4.91 y Group 2: 28.72 ± 6.86 y Combined: 79.07 ± 12.36 kg, 24.62 ± 2.70 kg/m^2^	Fitness: Agility, strength, cardiorespiratory fitness METHODS: Agility: Harres and L-run tests (mean of two attempts) Strength: Assessed via grip strength (mean of two attempts), standing long jump (mean of two attempts), and 1-min push-up and sit-up tests Fitness (VO_2max_): Estimated by repeated 20-m shuttle runs with progressively increasing speeds	Push-ups completed (F): Semester 1: 24.7 ± 11.7 Semester 3: 29.7 ± 12.5 ** Sit-ups completed (F): Semester 1: 49.5 ± 12.5 Semester 3: 53.6 ± 11.9 ** Grip strength, N—right (F): Semester 1: 377.1 ± 66.6 Semester 3: 3921.0 ± 63.9 ** Grip strength, N—left (F): Semester 1: 353.3 ± 60.3 Semester 3: 369.3 ± 60.6 ** VO_2max_, mL/kg/min (F): Semester 1: 40.4 ± 5.3 Semester 3: 40.9 ± 4.9 * Standing long jump, cm (F): Semester 1: 183.1 ± 22.8 Semester 3: 186.2 ± 221.0 ** L-run test, s (F): Semester 1: 6.7 ± 1.2 Semester 3: 6.5 ± 0.3 * Harres test, s (F): Semester 1: 13.9 ± 1.3 Semester 3: 14.0 ± 1.8 * *p* ≤ 0.03 (time), ** *p* < 0.001 (time)	Mental health: Self-reported mental health and perceived police ability	Police education in Sweden may not provide students with adequate means of maintaining their physical fitness. Police education training resulted in improved fitness and grip strength and increased push-ups, sit-ups, and long jump distance in women. Agility was either unchanged (Harres test) or declined (L-run test).
McClung et al., 2009 ‡ [53] RQ: 57.6%	Military Design: Prospective cohort (8 wk); U.S. Army basic combat training course (Fort Jackson, SC, USA) Primary outcomes: Iron status, performance, mood state	219 women soldiers Participants separated into two supplementation groups: Iron (*n* = 86) vs. Placebo (*n* = 85) Iron: 20.4 ± 4.2 y Placebo: 20.8 ± 4.4 y	Physical performance: 2-mile run time METHODS: Assessed at the end of basic combat training; results separated into supplementation groups and within each group, by iron status: Placebo (normal, iron-deficient, iron-deficient—anemia) and Iron (normal, iron-deficient, iron-deficient –anemia)	2-mile run time: Placebo (normal): 1057.8 ± 71.6 s Iron (normal): 1086.1 ± 86.7 s Placebo (iron-deficient): 1101.7 ± 102.7 s Iron (iron-deficient): 1053.3 ± 73.4 s * Placebo (anemia): 1192.2 ± 95.6 s ** Iron (anemia): 1082.2 ± 125.0 s * *p* < 0.05 (within-group, iron-normal vs. iron-deficient) ** *p* < 0.001 (between-group, placebo vs. iron-treated)	Iron status: Hemoglobin, red blood cell distribution width, ferritin, transferrin saturation, soluble transferrin receptor Mood state: POMS subscales (fatigue, confusion, depression, tension, anger, vigor)	For women who entered basic training with iron deficiency anemia, iron supplementation elicited a faster 2-mile run time post-training, but not in women who were iron-deficient or had normal iron levels. This could be due to improvements in hemoglobin concentrations in participants with iron deficiency anemia.
McFadden et al., 2024a [54] RQ: 72.7%	Military Design: Prospective cohort (13 wk); USMC basic training (Parris Island, SC, USA) Primary outcomes: Sex differences in workload, sleep, stress, and performance Part of a larger study, the USMC Gender-Integrated Recruit Training study	281 recruits (182 M, 99 F) 19 ± 2 y, 64.1 ± 7.1 kg, 23.3 ± 2.1 kg/m^2^, FM = 15.6 ± 3.8 kg, FFM = 48.5 ± 5.3 kg; Healthy, naïve to military life.	Lower body power: CMJ and CMJ_REL_ (peak power and relative peak power) Lower body strength: IMTP and IMTP_REL_ (peak force and relative peak force) METHODS: CMJ and CMJ_REL_: Three maximal repetitions ~2-min apart using bilateral force platforms (FDLite Forcedeck, VALD Performance, Sydney, Australia) IMTP and IMTP_REL_: Three familiarization repetitions ~2-min apart (50, 75, and 90% perceived maximal effort, 3-s duration) followed by two maximal repetitions (~5-s each) using a custom apparatus with bilateral force platforms (FDLite Forcedeck, VALD Performance, Sydney, Australia)	CMJ (F): Wk 2: 2329 ± 372 W Wk 11: 2201 ± 342 W * CMJ_REL_ (F): Wk 2: 37.6 ± 4.9 W/kg Wk 11: 35.7 ± 4.2 W/kg * IMTP (F): Wk 2: 1734 ± 363 N Wk 11: 1736 ± 357 N IMTP_REL_ (F): Wk 2: 27.5 ± 4.6 N/kg Wk 11: 28.0 ± 4.8 N/kg * * *p* < 0.05 (time)	Workload: Energy expenditure (relative to BM), distance, steps Stress: Salivary cortisol Sleep: Continuity and duration	The greatest physical demands occur earlier in the training program, yet the stress response was maintained throughout the training. Sex differences were observed for relative energy expenditure, distance, CMJ_REL_, and IMTP_REL_, and men experienced greater overall workloads.
McFadden et al., 2024b [55] RQ: 72.7%	Military Design: Prospective cohort (11 wk); USMC basic training (Parris Island, SC, USA) Primary outcomes: Performance, resilience, wearable tracking Part of a larger study, the USMC Gender-Integrated Recruit Training study	196 recruits (97 M, 99 F) Baseline characteristics NR	USMC performance battery: Physical fitness test score, combat fitness test score Lower body power: CMJ (peak power) Lower body strength: IMTP (peak force) METHODS: USMC performance battery: Physical fitness test (day 35 and 55): maximal number of pull-ups, maximal number of sit-ups in 2 min, and 3-mile timed run. Combat fitness test (day 27 and 47): 880-yard movement to contact, maximal number of ammo overhead presses in 2 min, 300-yard maneuver-under-fire event. CMJ: three maximal repetitions ~2-min apart using bilateral force platforms (FDLite Forcedeck, VALD Performance, Sydney, Australia) IMTP: three familiarization repetitions ~2-min apart (50, 75, and 90% perceived maximal effort, 3-s duration) followed by two maximal repetitions (~5-s each) using a custom apparatus with bilateral force platforms (FDLite Forcedeck, VALD Performance, Sydney, Australia)	Physical fitness score (F): Wk 5: 231 ± 37 Wk 8: 250 ± 31 Combat fitness score (F): Wk 4: 238 ± 34 Wk 7: 264 ± 24 CMJ (F): Wk 2: 2358 ± 370 W Wk 11: 2201 ± 342 W IMTP (F): Wk 2: 1747 ± 373 N Wk 11: 1736 ± 357 N *p*-values: NR for outcomes of interest	Resilience: Connor–Davidson Resilience Scale, workload, self-reported sleep, stress Wearable tracking: energy expenditure, distances, sleep, acceleration Other: Salivary cortisol	Higher physical and physiological workloads were associated with physical fitness and combat fitness scores in women, but not in men. BM and peak power were moderately related in women recruits.
Nindl et al., 2012 [56] RQ: 57.6%	Military Design: Prospective cohort (~4 months); Israeli Defense Force gender-integrated basic recruit training program (Tel Hashomer, Israel) Primary outcomes: Body composition, inflammation, fitness	194 (29 M, 93 F) recruits M: 19.1 ± 1.3 y, 72.6 ± 2.7 kg, VO_2max_ = 51.6 ± 1.1 mL/kg/min F: 18.8 ± 0.6 y, 61.6 ± 0.6 kg, VO_2max_ = 36.8 ± 0.7 mL/kg/min Collected in conjunction with Evans et al., 2008 [42]	Fitness: VO_2max_ METHODS: Unspecified treadmill protocol	VO_2max_ (F): Pre: 36.9 (0.7) mL/kg/min Post: 39.5 (0.6) mL/kg/min *p* < 0.05 (sex) *p* < 0.05 (time)	Body composition: BM, FM, FFM, BF Inflammation: IL-1β, IL-6, TNF-α, IGF-1, free IGF-1, IGFBP-1, IGFBP-2, IGFBP-3, IGFBP-4, IGFBP-5, IGFBP-6	Women undergoing gender-integrated basic military training experienced enhancements in cardiorespiratory fitness. Training-induced changes in IGF-1 were influenced by initial fitness level in women, but not in men. IGF-1 influenced changes in body composition and fitness in men, but not women.
Øfsteng et al., 2020 † [57] RQ: 63.6%	Military Design: Prospective cohort (17 days); 10-day military field exercise followed by 7 days of recovery Primary outcomes: Body composition and performance	38 2nd year soldiers (31 M, 7 F) at the Norwegian Defence Cyber Academy Participants (21.6 ± 0.8 y) divided into groups by HIGH and LOW protein intake: LOW: 76.2 ± 12.2 kg HIGH: 75.9 ± 12.2 kg	Lower body strength: one-RM leg press Lower body power: CMJ Upper body strength: one-RM bench press Anaerobic power: Wingate test (mean and peak) METHODS: Leg press: Warm-up of 40% and 75% of the expected one RM, first attempt performed at 5% below the expected one RM, load increased by 5% with each attempt using a pneumatic bilateral seated leg press machine (Keiser A420, Keiser Sport Health Equipment Inc., Fresno, CA, USA) CMJ: Best recorded of three attempts (30 s rest between attempts) on force plate (SG-9, Advanced Medical Technologies) Bench press: Same procedure as the one-RM leg press for progression of load Wingate test: 30-s warm-up (100 W at 60 rev/min) followed by 30-s all-out pedaling (torque factor of 0.67 for females and 0.70 for males) using a cycle ergometer (Lode Excalibur Sport, Lode BV, Groningen, The Netherlands)	One-RM leg press (F) *p* < 0.05 (sex) CMJ power (F) *p* < 0.05 (sex) *p* ≤ 0.02 (sex × time) One-RM bench press (F) *p* < 0.05 (sex) *p* ≤ 0.001 (sex × time) Wingate—peak power (F) *p* < 0.05 (sex) *p* ≤ 0.001 (sex × time) Wingate—mean power (F) *p* < 0.05 (sex) *p* ≤ 0.001 (sex × time) Data NR for women; significance levels reported	Body composition: BM, FM, FFM Energetics: Energy expenditure Sleep: Quantity Other: Testosterone, free testosterone, SHBG, IGF-1, cortisol, T3, T4, TSH, creatine kinase, testosterone/cortisol ratio	While men exhibited higher performance capabilities at baseline, they experienced a greater decline in performance than women over the course of the field exercise. Women soldiers displayed better recovery in the one-RM bench press and CMJ after 7 days than men.
O’Leary et al., 2018 ‡ [58] RQ: 72.7%	Military Design: Prospective cohort (1 day); 9.7-km loaded march (~90-min, 11 or 16 kg backpack and 4 kg rifle) as part of 14-wk British Army SE Phase One training (Army Training Centre, Pirbright, UK) Primary outcomes: Neuromuscular function and physiological strain	42 British Army Recruits (23 M, 19 F) M: 21 ± 3 y, 77.0 ± 0.09 kg, LM = 58.2 ± 8.2 kg, BF = 21.0 ± 7.1% F: 22 ± 4 y, 64.0 ± 7.2 kg, LM = 42.2 ± 3.5 kg, BF = 29.4 ± 5.6%	Neuromuscular function: MVC, vertical jump height METHODS: MVC: Force of right knee extensor assessed with three repetitions (~3 s each, ~15 s rest) via strain gauge (MIE, Digital Myometer, MIE Medical Research, Leeds, UK) Vertical jump: Assessed in triplicate (peak of three attempts recorded)	MVC (F): Pre: 400 (17) N Post: 365 (13) N *p* < 0.05 (time) Vertical jump height (F): Pre: 33.9 (1.0) cm Post: 32.2 (1.2) cm *p* < 0.05 (time)	Physiological strain: heart rate, rating of perceived exertion	Women experience greater physiological stress during military load carriage exercise vs. men, yet this did not contribute to a greater degree of neuromuscular fatigue. Women demonstrate considerable fatigue resistance, which is likely the result of differences in skeletal muscle physiology between sexes.
O’Leary et al., 2025 # [61] RQ: 75.8%	Military Design: Prospective cohort (13 wk); basic combat training program (Army Training Centre, Pirbright, UK) Primary outcomes: Body composition, performance, iron status, vitamin D status, bone metabolism markers	450 female British Army recruits Participants separated by oral contraceptive pill use: Non-users (*n* = 182), Combined (*n* = 124), Progestin-only (*n* = 144) Non-users: 22.9 ± 3.7 y, 64.8 ± 8.0 kg, 23.8 ± 2.4 kg/m^2^ Combined: 22.3 ± 3.3 y, 65.8 ± 8.1 kg, 23.9 ± 2.3 kg/m^2^ Progestin-only: 21.5 ± 3.1 y, 63.6 ± 8.3 kg, 23.4 ± 2.5 kg/m^2^	Endurance performance: 2.4-km run time Strength and power: Maximal lift strength, CMJ peak power output METHODS: 2.4-km run: Time to complete a maximal effort 2.4-km run on a standardized course after an 800-m warm-up Maximal strength: Maximal weight lifted using the power clean movement; weight increased by 5 kg every attempt until failure CMJ: Highest peak power value achieved from three attempts using a jump mat (Takei Scientific Instruments)	2.4-km run time (Δ, Wk 13 vs. Wk 1) * Non-users: −25 s (−33, −17) Combined: −30 s (−42, −19) Progestin-only: −27 s (−39, −15) Maximal strength (Δ, Wk 13 vs. Wk 1) * Non-users: 1.5 kg (−0.2, 3.1) Combined: 1.2 kg (−0.8, 3.1) ** Progestin: 1.9 kg (−0.5, 4.2) CMJ peak power (Δ, Wk 13 vs. Wk 1) * Non-users: 55 W (−5, 114) Combined: 27 W (−65, 119) Progestin-only: 2 W (−74, 77) * *p ≤* 0.02 (main effect, time), ** *p ≤* 0.05 (group, vs. non-users)	Body composition: FM, LM, areal bone mineral density (whole-body and regional: trunk, arms, and legs) Iron status: Ferritin, hemoglobin Vitamin D status: Total 25(OH)D Bone metabolism markers: PTH, βCTX, PINP	Basic training improved 2.4-km run time, maximal strength, and peak power. Decreases in 2.4-km run time were not different between contraceptive users. Maximal strength was significantly lower in the combined group, indicating that combined oral contraceptive pill use may impact muscle strength without affecting strength adaptations to training.
Szivak et al., 2018 †,# [22] RQ: 62.5%	Military Design: Prospective cohort (2 wk); U.S. Navy SERE training (Kittery and Rangeley, ME, USA) Primary outcomes: Neuroendocrine markers and performance	24 Marines (20 M, 4 F) Men were separated into high and low fit groups (*n* = 10 for each); women were not included in the final analysis High-fit M: 25.3 ± 4.4 y, 82.2 ± 17.9 kg Low-fit M: 25.2 ± 9.0 y, 85.2 ± 30.4 kg F: 22.3 ± 2.5 y, 67.2 ± 5.1 kg ∙	Strength: Handgrip (dominant hand) Lower body power: Vertical jump height METHODS: Strength: Highest recorded of three attempts (~5 s each) using handgrip dynamometer (Takei model 5001, Takei Scientific Instruments Co., LTD, Niigata, Japan) Vertical jump: Highest recorded of three CMJs using Vertec (JumpUSA, Sunnyvale, CA, USA)	Handgrip strength: Baseline: 33.5 ± 3.2 kg Stress: 30.0 ± 5.0 kg Vertical jump height: Baseline: 39.4 ± 6.8 cm Stress: 35.9 ± 7.4 cm *p*-values: NR for outcomes of interest	Neuroendocrine markers: Epinephrine, norepinephrine, dopamine, cortisol, testosterone, NPY	Physical performance responses to SERE training were similar between men and women. For men, dominant handgrip strength and vertical jump height were unchanged from baseline. Women were not included in the final analysis; therefore, future studies investigating stress responses to SERE training should include female participants whenever possible.
Vikmoen et al., 2020 † [68] RQ: 66.7%	Military Design: Prospective cohort (14 days) during a 6-day field-based Selection Exercise at Rena Military Camp (Rena, Norway) Primary outcomes: Body composition and performance	35 conscripts recruited from the Parachute Ranger Platoon (23 M) and the Special Reconnaissance Platoon (12 F) M: 19.3 ± 1.8 y, 79.5 ± 6.3 kg F: 19.4 ± 1.5 y, 67.7 ± 5.5 kg	Performance: CMJ height and maximal power, medicine ball throw, anaerobic performance (Evacuation test) METHODS: CMJ: three attempts (30 s rest) performed on a force plate (HUR Labs, Tampere, Finland), with the highest attempt recorded Medicine ball throw: 10 kg medicine ball starting at chest height, best throw of three to four attempts recorded Anaerobic performance: Assessed via Evacuation test (2 maximal exertion laps involving manikin drag)	CMJ height (F): Pre: 29.0 ± 3.6 cm Post 0 h: 23.5 ± 3.8 cm * Post 24 h: 24.2 ± 3.8 cm * Post 72 h: 24.8 ± 3.4 cm * Post 1 wk: 24.1 ± 3.7 cm * Post 2 wk: 26.3 ± 3.1 cm * CMJ maximal power (F): Pre: 2650 ± 384 W Post 0 h: 2265 ± 280 W * Post 24 h: 2387 ± 357 W * Post 72 h: 2394 ± 309 W * Post 1 wk: 2388 ± 333 W * Post 2 wk: 2540 ± 310 W Evacuation test (F): Pre: 45.6 ± 2.5 s Post 0 h: 67.0 ± 15.0 s * Post 24 h: 57.4 ± 4.9 s * Post 72 h: 51.9 ± 4.9 s * Post 1 wk: 49.7 ± 3.6 s * Post 2 wk: 47.2 ± 2.4 s * Medicine ball throw (F): Pre: 3.7 ± 0.3 m Post 0 h: 3.2 ± 0.3 m * Post 24 h: 3.4 ± 0.3 m Post 72 h: 3.5 ± 0.3 m Post 1 wk: 3.5 ± 0.3 m Post 2 wk: 3.6 ± 0.3 m * *p* < 0.05 (time, vs. Pre)	Body composition: BM, MM, FM Other: IGF-1, cortisol, testosterone, creatine kinase	Women recovered lower body strength faster than men. MM reductions during the field exercise did not contribute to sex differences in physical performance reductions.
Zurek et al., 2022 [69] RQ: 51.5%	Military Design: Prospective cohort (7 months); command training at the Military University of Land Forces (Wroclaw, Poland) Primary outcomes: Body composition and performance	126 cadets (108 M, 18 F) recruited in two phases (January and July 2021) Demographics NR	Strength: Handgrip, sit-ups Lower body power: Horizontal jump distance Running performance: Shuttle run, 1000-m run Executive function: Color Trails Test-2, shooting performance METHODS: Strength: Highest result of three attempts using dominant handgrip dynamometry Sit-ups: Number of completed attempts in 30 s Horizontal jump distance: The longest distance of two attempts was recorded Shuttle run: Time to complete 10 consecutive 5-m sprints 1000-m run: Time to complete 2.5 laps of a 400-m track as fast as possible Color Trails Test-2: Time to connect circles of alternating colors in ascending order of the number inside each circle (1–25) without lifting the pencil; one warm-up trial Shooting performance: Marksmanship assessed via various shooting tasks using carbines, machine guns, and pistols	Handgrip strength (F): Phase 1: 87.5 ± 14.2 kg ** Phase 2: 93.3 ± 10.7 kg ** Sit-ups completed (F): Phase 1: 28.1 ± 3.1 ** Phase 2: 27.9 ± 3.7 * Horizontal jump distance (F): Phase 1: 187.0 ± 17.0 cm ** Phase 2: 186.8 ± 19.9 cm ** Shuttle run time (F): Phase 1: 19.8 ± 0.8 s ** Phase 2: 19.2 ± 1.4 s ** 1000-m run time (F): Phase 1: 245.4 ± 14.3 s ** Phase 2: 239.9 ± 22.7 s ** Color Trails Test-2 (F): Phase 1: 60.2 ± 12.0 s Phase 2: 58.7 ± 11.4 s Shooting performance (F): Phase 1: 3.1 ± 1.0 Phase 2: 4.4 ± 0.4 * *p ≤* 0.02 (sex), ** *p ≤* 0.0001 (sex)	Body composition: BM, BMI, BF, MM	All performance variables differed between men and women except for measures of executive function. Specific emphasis should be placed on marksmanship training and strength development during cadet training programs.

^a^ Summarized as mean ± standard deviation (SD), mean (standard error), Δ (95% confidence interval; CI) or median [interquartile range; IQR]. BF, Body fat. BM, Body mass. BMI, Body mass index. CMJ, Countermovement vertical jump. CMJ_REL_, CMJ relative to body mass. FFM, Fat-free mass. FM, Fat mass. F, Female. IGF-1, Insulin-like growth factor 1. IMTP, Isometric mid-thigh pull. IMTP_REL_, IMTP relative to body mass. LM, Lean mass. M, Male. MFPTT, Maximum force prior to takeoff. MM, Muscle mass. MVC, Maximal voluntary contraction. N, Newtons. NR, Not reported. PA, Physical activity. PINP, Procollagen I N-terminal peptide. RM, Repetition maximum. RQ, Reporting quality. SERE, Survival, Evasion, Resistance, and Escape. USMC, U.S. Marine Corps. W, Watts. VO_2max_, maximal oxygen uptake. VO_2peak_, peak oxygen uptake. ‡ Data were extracted via WebPlotDigitizer v5 (https://automeris.io/) for the following studies: Coge et al., 2024 [37], McClung et al., 2009 [53], O’Leary et al., 2018 [58]. # Identified via hand-searching (not with an electronic database search). † Denotes a study that includes a recovery period.

## Data Availability

All reported studies/experiments have been previously published with available data to support findings.

## Supplementary Materials

The following supporting information can be downloaded at: https://www.mdpi.com/article/10.3390/metabo15080506/s1. Table S1. Full search strategy used for each electronic database; Table S2. Description of potentially eligible studies that were excluded from the final sample following full-text review (*n* = 64); Table S3. Reporting completeness for individual checklist items (expressed as a percentage of items completely reported) for the total sample of included studies and each outcome of interest. References [92,95,96,97,98,99,100,101,102,103,104,105,106,107,108,109,110,111,112,113,114,115,116,117,118,119,120,121,122,123,124,125,126,127,128,129,130,131,132,133,134,135,136,137,138,139,140,141,142,143,144,145,146,147,148,149,150,151,152,153,154,155,156,157] are cited in the supplementary materials.

## Abbreviations

The following abbreviations are used in the main text of the manuscript:

BF	Body fat
BM	Body mass
BMI	Body mass index
BMM	Bone mineral mass
CCP	Combined contraceptive pill
CFT	Combat fitness test
CK	Creatine kinase
CMJ	Countermovement jump
CMJREL	CMJ relative to body mass
COCP	Combined oral contraceptive
CRH	Corticotropin-releasing hormone
CRP	C-reactive protein
CTT-2	Color trails test
CTx	C-telopeptide cross-links of type I collagen
DBP	Diastolic blood pressure
DHEA	Dihydroepiandrostenedione
DHEA-S	Dihydroepiandrostenedione sulfate
DLM	Dry lean mass
DLW	Doubly labeled water
DXA	Dual energy X-ray absorptiometry
EEREL	Energy expenditure relative to body mass
EMS	Emergency medical services
F	Female
FAI	Free androgen index
FFA	Free fatty acids
FFM	Fat-free mass
FM	Fat mass
FSH	Follicle-stimulating hormone
GnRH	Gonadotropin-releasing hormone
GSH	Glutathione
GPX	Glutathione peroxidase
HCC	Hair cortisol concentration
HDL	High-density lipoprotein
HOMA2 IR	Homeostatic modeling assessment of insulin resistance 2
HPA	Hypothalamic–pituitary–adrenal
HPG	Hypothalamic–pituitary–gonadal
HPO	Hypothalamic–pituitary–ovarian
HR	Heart rate
HRV	Heart rate variability
IFN-γ	Interferon γ
IGF-1	Insulin-like growth factor
LEA	Low energy availability
PRISMA-ScR	Preferred Reporting Items for Systematic Review and Meta-Analyses extension for Scoping Reviews
REDs	Relative Energy Deficiency in Sport
SD	Standard deviation
SNS	Sympathetic nervous system
U.S.	United States
