# Supplementary material for: Impact of Stress on Adrenal and Neuroendocrine Responses, Body Composition, and Physical Performance Amongst Women in Demanding Tactical Occupations: A Scoping Review
Source: Metabolites. 2025 Jul 29;15(8):506. doi: 10.3390/metabo15080506 (PMC12388157; doi:10.3390/metabo15080506)
Supplement: Supplementary file 1 [file metabolites-15-00506-s001.zip › TABLE-S1_Full-Search-Strategy.pdf]

TABLE S1. Full search strategy used for each electronic database.

Electronic search strategies for PubMed, Scopus (including EMBASE), and Web of Science are listed below. For each search, no start date was applied, and databases were searched from their inception or date of the earliest available publication.

<table>
<tr><td>PubMed (including MEDLINE)
Vendor/Platform: National Library of Medicine</td><td>Coverage: Date of inception 1940’s – February 26, 2025
Hits: 132</td></tr>
<tr><td colspan="2">PubMed was searched with appropriate Medical Subject Headings (MeSH) incorporated into hedges.
("tactical athlete"[Title/Abstract] OR "tactical athletes"[Title/Abstract] OR "war fighter"[Title/Abstract] OR "warfighter"[Title/Abstract] OR "war fighters"[Title/Abstract] OR "warfighters"[Title/Abstract] OR "police"[Title/Abstract] OR "police"[MeSH Terms] OR "military"[Title/Abstract] OR "military personnel*"[MeSH Terms] OR "emergency medical service*"[Title/Abstract] OR "firefighter*"[Title/Abstract] OR "fire fighter*"[Title/Abstract] OR "law enforcement"[Title/Abstract] OR "Occupations"[MeSH Terms])
AND ("stress, physiological"[MeSH Terms] OR "stress, psychological"[MeSH Terms] OR "psychological stress"[Title/Abstract] OR "psychophysiology*"[Title/Abstract] OR "musculoskeletal and neural physiological phenomena/immunology"[MeSH Terms] OR "physiological stress"[Title/Abstract] OR "Sympathoadrenal System"[MeSH Terms] OR "cortisol"[Title/Abstract] OR "catecholamine"[Title/Abstract] OR "catecholamines"[Title/Abstract] OR "inflammation"[Title/Abstract] OR "epinephrine/blood"[MeSH Terms] OR "hormones/immunology"[MeSH Terms] OR "hormones/physiology"[MeSH Terms] OR "norepinephrine/blood"[MeSH Terms] OR "hydrocortisone/blood"[MeSH Terms] OR "pituitary hormones/blood"[MeSH Terms] OR "hydrocortisone/blood"[MeSH Terms] OR "biomarkers"[MeSH Terms] OR "hormones/blood*"[MeSH Terms] OR "stress, physiological/blood*"[MeSH Terms] OR "occupational stress"[Title/Abstract] OR "occupational strain"[Title/Abstract] OR "job strain"[Title/Abstract] OR "job stress"[Title/Abstract] OR "work strain"[Title/Abstract] OR "work stress"[Title/Abstract])
AND ("Body Composition"[MeSH Terms] OR "body fat"[Title/Abstract] OR "muscle mass"[Title/Abstract] OR "lean mass"[Title/Abstract] OR "fat free mass"[Title/Abstract] OR "fat mass"[Title/Abstract] OR "occupational performance"[Title/Abstract] OR "performance"[Title/Abstract] OR "Exercise test"[MeSH Terms] OR "task performance"[All Fields] OR "recovery"[Title/Abstract] OR "readiness"[Title/Abstract] OR "Work tolerance"[Title/Abstract] OR "load carriage"[Title/Abstract] OR "muscle strength/physiology"[MeSH Terms] OR "physical endurance/physiology"[MeSH Terms])
AND (("women"[Title/Abstract] OR "woman"[Title/Abstract] OR "female"[Title/Abstract] OR "Sex Factors"[MeSH Terms] OR "sex differences"[Title/Abstract] OR "sex matched"[Title/Abstract])</td></tr>
<tr><td>Scopus (including EMBASE)
Vendor/platform: Elsevier SciVerse</td><td>Search dates: Date of inception 1960 – February 26, 2025
Hits: 298</td></tr>
<tr><td colspan="2">Scopus was searched for the following terms in the “Article title, abstract, keywords.” Filters were set for Document Type (include Journal articles and reviews).</td></tr>
</table>

Line 1 (in article, title, abstract, keywords): ("tactical athlete*" OR "war fighter*" OR "warfighter*" OR "police" OR "military" OR "Military Personnel*" OR "firefighter*" OR "fire fighter*" OR "law enforcement")

AND Line 2: ("physiological stress" OR "occupational stress" OR "occupational strain" OR "job strain" OR "job stress" OR "work strain" OR "work stress" OR "psychological stress" OR "psychophysiology stress" OR "occupational stress" OR "sympathoadrenal system" OR "sympathoadrenal*" OR "adrenal*" OR "sympathetic nervous system" OR "sympathetic activity" OR "cortisol" OR "catecholamine" OR "catecholamines" OR "inflammation" OR "immune function" OR "immunology" OR "epinephrine" OR "norepinephrine" OR "hydrocortisone*" OR "cortisol" OR "pituitary hormone*")

AND Line 3: ("body composition" OR "body fat" OR "muscle mass" OR "lean mass" "fat free mass" OR "adiposity" OR "fat mass" OR "performance" OR "exercise test*" OR "fitness test*" OR "task performance*" OR "occupational performance" OR "recovery" OR "readiness" OR "work tolerance" OR "load carriage" OR "muscle strength" OR "muscular strength" OR "*strength" OR "physical endurance" OR "physical fitness" OR "*endurance")

AND Line 4 (in article, title, abstract, keywords): (women OR woman OR female OR "sex factors" OR "sex differences" OR "sex matched")

**Web of Science (i.e., Web of Knowledge)**	Coverage: Earliest date available 1974 – February 26, 2025
Vendor/platform: Thomson Reuters	Hits: 240

Web of Science was searched twice using a slightly different combination of terms. For both, the following terms were searched as “Topic” words (i.e., searches the title, abstract, keywords plus, and author keywords).

Search 1 (Hits = 111):

Line 1 (in topic): ("tactical athlete*" OR "war fighter*" OR "warfighter*" OR "police" OR "military" OR "Military Personnel*" OR "firefighter*" OR "fire fighter*" OR "law enforcement")

AND Line 2 (in topic): ("physiological stress" OR "occupational stress" OR "occupational strain" OR "job strain" OR "job stress" OR "work strain" OR "work stress" OR "psychological stress" OR "psychophysiology stress" OR "occupational stress" OR "sympathoadrenal system" OR "sympathoadrenal*" OR "adrenal*" OR "sympathetic nervous system" OR "sympathetic activity" OR "cortisol" OR "catecholamine" OR "catecholamines" OR "inflammation" OR "immune function" OR "immunology" OR "epinephrine" OR "norepinephrine" OR "hydrocortisone*" OR "cortisol" OR "pituitary hormone*")

AND Line 3 (in topic): ("body composition" OR "body fat" OR "muscle mass" OR "lean mass" "fat free mass" OR "adiposity" OR "fat mass" OR "performance" OR "exercise test*" OR "fitness test*" OR "task performance*" OR "occupational performance" OR "recovery" OR "readiness" OR "work tolerance" OR "load carriage" OR "muscle strength" OR "muscular strength" OR "*strength" OR "physical endurance" OR "physical fitness" OR "*endurance")

AND Line 4 (in topic): ("women OR woman OR female OR "sex factors" OR "sex differences" OR "sex matched")

Search 2 (Hits = 129):

Line 1 (in topic): ("tactical athlete*" OR "war fighter*" OR "warfighter*" OR "police" OR "military" OR "Military Personnel*" OR "firefighter*" OR "fire fighter*" OR "law enforcement")

AND Line 2 (all fields topic): ("physiological stress" OR "occupational stress" OR "occupational strain" OR "job strain" OR "job stress" OR "work strain" OR "work stress" OR "psychological stress" OR "psychophysiology stress" OR "occupational stress" OR "sympathoadrenal system" OR "sympathoadrenal*" OR "adrenal*" OR "sympathetic nervous system" OR "sympathetic activity" OR "cortisol" OR "catecholamine" OR "catecholamines" OR "inflammation" OR "immune function" OR "immunology" OR "epinephrine" OR "norepinephrine" OR "hydrocortisone*" OR "cortisol" OR "pituitary hormone*")

AND Line 3 (all fields): ("body composition" OR "body fat" OR "muscle mass" OR "lean mass" "fat free mass" OR "adiposity" OR "fat mass" OR "performance" OR "exercise test*" OR "fitness test*" OR "task performance*" OR "occupational performance" OR "recovery" OR "readiness" OR "work tolerance" OR "load carriage" OR "muscle strength" OR "muscular strength" OR "physical endurance" OR "physical fitness")

AND Line 4 (in topic): ("women OR woman OR female OR "sex factors" OR "sex differences" OR "sex matched")
