# Supplementary material for: Impact of Stress on Adrenal and Neuroendocrine Responses, Body Composition, and Physical Performance Amongst Women in Demanding Tactical Occupations: A Scoping Review
Source: Metabolites. 2025 Jul 29;15(8):506. doi: 10.3390/metabo15080506 (PMC12388157; doi:10.3390/metabo15080506)
Supplement: Supplementary file 1 [file metabolites-15-00506-s001.zip › TABLE-S2_Excluded-Studies-Full-Text.pdf]

TABLE S2. Description of potentially eligible studies that were excluded from the final sample following full-text review (*n* = 64).

Primary Reason for Exclusion	Number of Studies	Proportion of Sample
Women included in study; data not disaggregated by sex	36	56.3%
Study design / article type	9	14.1%
No outcomes of interest evaluated in study	7	10.9%
No women included in study	6	9.4 %
Data not reported for outcome(s) of interest	2	3.1%
Non-tactical population (e.g., civilian)	3	4.7%
Non-English language publication	1	1.6%

	Author, Year	Domain (Occupation) [a]	Outcome(s) of Interest	Primary Reason for Exclusion
1	Bartman et al., 2024	Civilian (NA)	—	No outcomes of interest evaluated in study
2	Berryman et al., 2022	Military (U.S. Marine Corps; SERE candidates)	Body composition	No women included in study
3	Brown et al., 2018	Military (U.S. Active-duty and veterans)	Body composition	No women included in study
4	Bulmer et al., 2022	Military (Australian Army; Basic Military Training recruits)	Occupational performance	Women included in study; data not disaggregated by sex
5	Chitra et al., 2021	Police (Armed Reserve Police Unit – India)	—	No outcomes of interest evaluated in study
6	Choi et al., 2016	Fire (Career FFs)	Body composition	Women included in study; data not disaggregated by sex
7	Christison et al., 2021	Fire (Wildland FFs; Interagency Hotshot Crew)	Adrenal stress<br>Body composition<br>Occupational performance	Women included in study; data not disaggregated by sex
8	Christison et al., 2023	Fire (Wildland FFs)	Body composition	Women included in study; data not disaggregated by sex
9	Christodoulou et al., 2022	Fire (Career FFs)	Body composition	Women included in study; data not disaggregated by sex
10	Coker et al., 2019	Fire (Alaskan Wildland FFs)	Body composition	Women included in study; data not

	Author, Year	Domain (Occupation) [a]	Outcome(s) of Interest	Primary Reason for Exclusion
				disaggregated by sex
11	Conkright et al., 2022	Military		Narrative Review
12	Coombs et al., 2023	Military (Active and Reserve servicewomen and civilians – United Kingdom)	Body composition<br>Occupational performance	Clinical Trial Protocol (No data reported)
13	Corbett et al., 2023	Military		Invited (Narrative) Review
14	Di Nota et al., 2024	Police (Experienced Officers enrolled in the Masters of Police Services Program – Finland)	Adrenal stress	Women included in study; data not disaggregated by sex
15	Diaz-Manzano et al., 2018	Military (Spanish Army Soldiers)	Adrenal stress<br>Occupational performance	No women included in study
16	Drain et al., 2017	Military (Australian Army; Basic Military Training recruits)	Adrenal stress<br>Body composition	Women included in study; data not disaggregated by sex
17	Epstein et al., 2013	Military		Invited (Narrative) Review
18	Fagnant et al., 2019	Military (U.S. Army, Air Force, and Marine Corps recruits)	Body composition	Women included in study; data not disaggregated by sex
19	Farina et al., 2017	Military (U.S. Army Special Operation Forces Soldiers)	—	No outcomes of interest evaluated in study
20	Farina et al., 2025	Military (U.S. Army Soldiers; Special Forces Assessment and Selection course candidates)	Adrenal stress<br>Body composition<br>Occupational performance	No women included in study
21	Flood and Keegan, 2022	Military		Narrative Review
22	Forse et al., 2024	Military (U.S. Marine Corps Officer Candidate School trainees)	Adrenal stress<br>Occupational performance	Women included in study; data not disaggregated by sex
23	Friedl, 2025	Military		Special Report
24	Gaffney-Stomberg et al., 2014	Military (U.S. Army; Basic Combat Training recruits)	Body composition	Women included in study; data not disaggregated by sex
25	Giersch et al., 2022	Military		Narrative Review
26	Gifford et al., 2017	Military		Narrative Review
27	Gnacinski et al., 2016	Fire (Recruits)	Body composition<br>Occupational performance	Women included in study; data not disaggregated by sex
28	Gu et al., 2012	Police (Active-duty officers)	Body composition	No women included in study

	Author, Year	Domain (Occupation) [a]	Outcome(s) of Interest	Primary Reason for Exclusion
29	Harlow et al., 2024	Military (Active-duty personnel)		Systematic Review
30	Heblich et al., 2020 [b]	Civilian (NR – Germany)	Occupational performance	Non-tactical population (subjects simulated FF stress)
31	Hormeño-Holgado et al., 2019	Military (Spanish Air Force Soldiers; Air Security Force Unit)	Adrenal stress<br>Occupational performance	Women included in study; data not disaggregated by sex
32	Hourani et al., 2006	Military (Active-duty service members – all branches)	Occupational performance	Women included in study; data not disaggregated by sex
33	Ibrahim et al., 2024	Military (German Armed Forces recruits)	Occupational performance	Women included in study; data not disaggregated by sex
34	Jayne et al., 2020	Military (U.S. Army; Active-duty Soldiers)	Body composition	Women included in study; data not disaggregated by sex
35	Jayne et al., 2020b	Military (U.S. Army; Active-duty Soldiers)	—	No outcomes of interest evaluated in study
36	Korre et al., 2019	Police (Recruits)	Body composition<br>Occupational performance	Women included in study; data not disaggregated by sex
37	Kukić et al., 2020	Police (Cadets – Serbia)	Body composition<br>Occupational performance	Women included in study; data not disaggregated by sex
38	Kukić et al., 2023	Police (Cadets – Serbia)		Women included in study; data not disaggregated by sex
39	Lan et al., 2021	Fire (Recruits)	Body composition<br>Occupational performance	Women included in study; data not disaggregated by sex
40	Lieberman et al., 2015	Military (Active-duty U.S. Navy or Marine personnel; SERE candidates)	Adrenal stress	Women included in study (n=2); data not disaggregated by sex
41	Lin et al., 2021	Military (Taiwan Armed Forces; Active-duty service members)	Occupational performance	Women included in study; data not disaggregated by sex
42	McAllister et al., 2020	Civilian (University students)	Adrenal stress	Non-tactical population (subjects role-played as police officers)
43	McAllister et al., 2021	Civilian (University students)	Adrenal stress	Non-tactical population (subjects role-played as police officers)
44	McClung et al., 2009	Military (U.S. Army; Basic Combat Training recruits)	Occupational performance	Data not reported for outcome(s) of interest
45	Morse et al., 2024	Military (Active-duty U.S. Navy personnel)	Body composition	Women included in study; data not disaggregated by sex

	Author, Year	Domain (Occupation) [a]	Outcome(s) of Interest	Primary Reason for Exclusion
46	Ojanen et al., 2020	Military (Finnish Defense Forces; Active-duty soldiers)	Occupational performance	No women included in study
47	Penatzer et al., 2020	Military (U.S. Air Force Reserve Officers' Training Corps cadets)	—	No outcomes of interest evaluated in study
48	Pineda Calderón et al., 2023	Police (National Police of Ecuador; Active-duty officers)	Occupational performance	Non-English language publication (Abstract available in English)
49	Proessl et al., 2022	Military (Active-duty or recent service members; Army, Marines, Navy, Air Force or Reserve Officer Training Corps)	Occupational performance	Women included in study; data not disaggregated by sex
50	Ramey et al., 2011	Police (Active-duty officers)	—	No outcomes of interest evaluated in study
51	Rodas et al., 2022	Police (Custody Assistance Training Academy recruits)	Body composition<br>Occupational performance	Women included in study; data not disaggregated by sex
52	Rosalky et al., 2017	Fire (Career FFs)	Adrenal stress	Women included in study; data not disaggregated by sex
53	Schilling et al., 2019	Police (Active-duty officers – Switzerland)	Body composition<br>Occupational performance	Women included in study; data not disaggregated by sex
54	Schilling et al., 2020	Police (Active-duty officers – Switzerland)	Occupational performance	Women included in study; data not disaggregated by sex
55	Schilling et al., 2020b	Police (Active-duty officers – Switzerland)	Body composition	Women included in study; data not disaggregated by sex
56	Scott et al., 2019	Military (U.S. Marine Corps; Basic Training recruits)	Adrenal stress	Women included in study; data not disaggregated by sex
57	Tait et al., 2022	Military (Australian Army Soldiers in the Combat Engineer Training course)	Adrenal stress	Women included in study; data not disaggregated by sex
58	Taylor et al., 2014	Military (Active-duty U.S. soldiers; SERE candidates)	Adrenal stress	Data not reported for outcome(s) of interest
59	Tegeler et al., 2017	Military (Active-duty soldiers or recent veterans)	Adrenal stress<br>Occupational performance	Women included in study; data not disaggregated by sex
60	Tingestad et al., 2019	Military (Canadian Armed Forces members)	Adrenal stress<br>Occupational performance	Women included in study; data not disaggregated by sex
61	Toczko et al., 2023	Fire (Career FFs)	Body composition	Women included in study; data not

	Author, Year	Domain (Occupation) [a]	Outcome(s) of Interest	Primary Reason for Exclusion
				disaggregated by sex
62	Vicente-Rodríguez et al., 2020	Military (Spanish Air Force; Active-duty Air Crew members)	Adrenal stress<br>Occupational performance	Women included in study; data not disaggregated by sex
63	Visconti et al., 2024	Military (U.S. Army Operators; 10th Special Forces Group Operational Detachment)	Adrenal stress	Women included in study; data not disaggregated by sex
64	Watkins et al., 2019	Fire (Fire Service Instructors – United Kingdom)	—	No outcomes of interest evaluated in study

FF, Fire fighter. NA, Not applicable. SERE, Survival, Evasion, Resistance, and Escape.

[a] Study samples outside of the U.S. are noted. [b] Non-English language (German) publication (abstract available in English).

References

1. Bartman NE, Hess HW, Colburn D, Temple J, Hostler D. Heat strain in different hot environments hiking in wildland firefighting garments. Appl Physiol Nutr Metab. 2025 Jan 1;50:1-14. doi: 10.1139/apnm-2024-0240. Epub 2024 Oct 31.
2. Berryman CE, McClung HL, Sepowitz JJ, Gaffney-Stomberg E, Ferrando AA, McClung JP, Pasiakos SM. Testosterone status following short-term, severe energy deficit is associated with fat-free mass loss in US Marines. Physiol Rep. 2022 Sep;10(18):e15461. doi: 10.14814/phy2.15461.
3. Brown RM, Tang X, Dreer LE, Driver S, Pugh MJ, Martin AM, McKenzie-Hartman T, Shea T, Silva MA, Nakase-Richardson R. Change in body mass index within the first-year post-injury: A VA traumatic brain injury (TBI) model systems study. Brain Inj. 2018;32(8):986-993. doi: 10.1080/02699052.2018.1468575. Epub 2018 Apr 27
4. Bulmer S, Aisbett B, Drain JR, Roberts S, Gastin PB, Tait J, Main LC. Sleep of recruits throughout basic military training and its relationships with stress, recovery, and fatigue. Int Arch Occup Environ Health. 2022 Aug;95(6):1331-1342. doi: 10.1007/s00420-022-01845-9. Epub 2022 Feb 28.
5. Chitra T, Karunanidhi S. The impact of resilience training on occupational stress, resilience, job satisfaction, and psychological well-being of female police officers. J Police Crim Psych. 2021 Mar;36(1):8-23. doi: 10.1007/s11896-018-9294-9.
6. Choi B, Schnall P, Dobson M. Twenty-four-hour work shifts, increased job demands, and elevated blood pressure in professional firefighters. Int Arch Occup Environ Health. 2016 Oct;89(7):1111-25. doi: 10.1007/s00420-016-1151-5. Epub 2016 Jul 1.
7. Christison KS, Gurney SC, Sol JA, Williamson-Reisdorph CM, Quindry TS, Quindry JC, Dumke CL. Muscle damage and overreaching during wildland firefighter critical training. J Occup Environ Med. 2021 Apr 1;63(4):350-356. doi: 10.1097/JOM.0000000000002149.
8. Christison KS, Sol JA, Gurney SC, Dumke CL. Wildland Firefighter Critical Training Elicits Positive Adaptations to Markers of Cardiovascular and Metabolic Health. Wilderness Environ Med. 2023 Sep;34(3):328-333. doi: 10.1016/j.wem.2023.04.003. Epub 2023 May 29.
9. Christodoulou A, Christophi CA, Sotos-Prieto M, Moffatt S, Kales SN. Eating habits among US firefighters and association with cardiometabolic outcomes. Nutrients. 2022 Jul 4;14(13):2762. doi: 10.3390/nu14132762.
10. Coker RH, Murphy CJ, Johannsen M, Galvin G, Ruby BC. Wildland firefighting: adverse influence on indices of metabolic and cardiovascular health. J Occup Environ Med. 2019 Mar;61(3):e91-e94. doi: 10.1097/JOM.0000000000001535.
11. Conkright WR, O'Leary TJ, Wardle SL, Greeves JP, Beckner ME, Nindl BC. Sex differences in the physical performance, physiological, and psycho-cognitive responses to military operational stress. Eur J Sport Sci. 2022 Jan;22(1):99-111. doi: 10.1080/17461391.2021.1916082. Epub 2021 May 10.
12. Coombs CV, Wardle SL, Shroff R, Eisenhauer A, Tang JC, Fraser WD, Greeves JP, O'Leary TJ. The effect of calcium supplementation on calcium and bone metabolism during load carriage in women: protocol for a randomised controlled crossover trial. BMC Musculoskelet Disord. 2023 Jun 16;24(1):496. doi: 10.1186/s12891-023-06600-w
13. Corbett J, Wright J, Tipton MJ. Sex differences in response to exercise heat stress in the context of the military environment. BMJ Mil Health. 2023 Feb;169(1):94-101. doi: 10.1136/jramc-2019-001253. Epub 2020 Feb 23.

14. Di Nota PM, Scott SC, Huhta JM, Gustafsberg H, Andersen JP. Physiological responses to organizational stressors among police managers. Appl Psychophysiol Biofeedback. 2024 Mar;49(1):85-102. doi: 10.1007/s10484-023-09613-2. Epub 2024 Jan 20.
15. Diaz-Manzano M, Fuentes JP, Fernandez-Lucas J, Aznar-Lain S, Clemente-Suárez VJ. Higher use of techniques studied and performance in melee combat produce a higher psychophysiological stress response. Stress Health. 2018 Dec;34(5):622-628. doi: 10.1002/smi.2829. Epub 2018 Jul 27.
16. Drain JR, Groeller H, Burley SD, Nindl BC. Hormonal response patterns are differentially influenced by physical conditioning programs during basic military training. J Sci Med Sport. 2017 Nov;20 Suppl 4:S98-S103. doi: 10.1016/j.jsams.2017.08.020. Epub 2017 Sep 6.
17. Epstein Y, Yanovich R, Moran DS, Heled Y. Physiological employment standards IV: integration of women in combat units physiological and medical considerations. Eur J Appl Physiol. 2013 Nov;113(11):2673-90. doi: 10.1007/s00421-012-2558-7. Epub 2012 Dec 14.
18. Fagnant HS, Armstrong NJ, Lutz LJ, Nakayama AT, Guerriere KI, Ruthazer R, Cole RE, McClung JP, Gaffney-Stomberg E, Karl JP. Self-reported eating behaviors of military recruits are associated with body mass index at military accession and change during initial military training. Appetite. 2019 Nov 1;142:104348. doi: 10.1016/j.appet.2019.104348. Epub 2019 Jul 9.
19. Farina EK, Taylor JC, Means GE, Murphy NE, Pasiakos SM, Lieberman HR, McClung JP. Effects of deployment on diet quality and nutritional status markers of elite US Army special operations forces soldiers. Nutr J. 2017 Jul 3;16(1):41. doi: 10.1186/s12937-017-0262-5.
20. Farina EK, Stein JA, Thompson LA, Knapik JJ, Pasiakos SM, McClung JP, Lieberman HR. Longitudinal changes in psychological, physiological, and nutritional measures and predictors of success in Special Forces training. Physiol Behav. 2025 Mar 15;291:114790. doi: 10.1016/j.physbeh.2024.114790. Epub 2025 Jan 13.
21. Flood A, Keegan RJ. Cognitive resilience to psychological stress in military personnel. Front Psychol. 2022 Mar 16;13:809003. doi: 10.3389/fpsyg.2022.809003.
22. Forse JN, Koltun KJ, Bird MB, Lovalekar M, Feigel ED, Steele EJ, Martin BJ, Nindl BC. Low psychological resilience and physical fitness predict attrition from US Marine Corps Officer Candidate School training. Mil Psychol. 2024 Oct 21:1-10. doi: 10.1080/08995605.2024.2403826. Epub ahead of print.
23. Friedl KE. Biomedical research on health and performance of military women: accomplishments of the Defense Women's Health Research Program (DWHRP). J Womens Health (Larchmt). 2005 Nov;14(9):764-802. doi: 10.1089/jwh.2005.14.764.
24. Gaffney-Stomberg E, Lutz LJ, Rood JC, Cable SJ, Pasiakos SM, Young AJ, McClung JP. Calcium and vitamin D supplementation maintains parathyroid hormone and improves bone density during initial military training: a randomized, double-blind, placebo controlled trial. Bone. 2014 Nov;68:46-56. doi: 10.1016/j.bone.2014.08.002. Epub 2014 Aug 10.
25. Giersch GEW, Charkoudian N, McClung HL. The rise of the female warfighter: Physiology, performance, and future directions. Med Sci Sports Exerc. 2022 Apr 1;54(4):683-691. doi: 10.1249/MSS.0000000000002840.
26. Gifford RM, Reynolds RM, Greeves J, Anderson RA, Woods DR. Reproductive dysfunction and associated pathology in women undergoing military training. BMJ Military Health. 2017 Oct;163(5):301-310. doi: 10.1136/jramc-2016-000727. Epub 2017 Feb 17
27. Gnacinski SL, Ebersole KT, Cornell DJ, Mims J, Zamzow A, Meyer BB. Firefighters' cardiovascular health and fitness: An observation of adaptations that occur during firefighter training academies. Work. 2016 Mar 9;54(1):43-50. doi: 10.3233/WOR-162266.

28. Gu JK, Charles LE, Burchfiel CM, Fekedulegn D, Sarkisian K, Andrew ME, Ma C, Violanti JM. Long work hours and adiposity among police officers in a US northeast city. J Occup Environ Med. 2012 Nov;54(11):1374-81. doi: 10.1097/JOM.0b013e31825f2bea.
29. Harlow J, Blodgett K, Stedman J, Pojednic R. Dietary supplementation on physical performance and recovery in active-duty military personnel: A systematic review of randomized and quasi-experimental controlled trials. Nutrients. 2024 Aug 17;16(16):2746. doi: 10.3390/nu16162746.
30. Heblich F, Kähler W. Increased stress for firefighters due to wearing full-face masks? Zentralblatt Arb Arb. 2020 Jan;70:1-7.
31. Hormeño-Holgado AJ, Perez-Martinez MA, Clemente-Suárez VJ. Psychophysiological response of air mobile protection teams in an air accident manoeuvre. Physiol Behav. 2019 Feb 1;199:79-83. doi: 10.1016/j.physbeh.2018.11.006. Epub 2018 Nov 8.
32. Hourani LL, Williams TV, Kress AM. Stress, mental health, and job performance among active-duty military personnel: findings from the 2002 Department of Defense Health-Related Behaviors Survey. Mil Med. 2006 Sep;171(9):849-56. doi: 10.7205/milmed.171.9.849.
33. Ibrahim F, Schumacher J, Schwandt L, Herzberg PY. The first shot counts the most: Tactical breathing as an intervention to increase marksmanship accuracy in student officers. Mil Psychol. 2024 Nov;36(6):689-700. doi: 10.1080/08995605.2023.2258737. Epub 2023 Sep 21.
34. Jayne JM, Ayala R, Karl JP, Deschamps BA, McGraw SM, O'connor K, DiChiara AJ, Cole RE. Body weight status, perceived stress, and emotional eating among US Army Soldiers: A mediator model. Eat Behav. 2020 Jan;36:101367. doi: 10.1016/j.eatbeh.2020.101367. Epub 2020 Jan 16.
35. Jayne JM, Blake CE, Frongillo EA, Liese AD, Cai B, Nelson DA, Kurina LM, Funderburk L. Stressful life changes and their relationship to nutrition-related health outcomes among US Army Soldiers. J Prim Prev. 2020 Apr;41(2):171-189. doi: 10.1007/s10935-020-00583-3.
36. Korre M, Loh K, Eshleman EJ, Lessa FS, Porto LG, Christophi CA, Kales SN. Recruit fitness and police academy performance: a prospective validation study. Occup Med (Lond). 2019 Dec 31;69(8-9):541-548. doi: 10.1093/occmed/kqz110.
37. Kukić F, Koropanovski N, Janković R, Cvorovic A, Dawes J, Lockie R, Orr RM, Dopsaj M. Association of sex-related differences in body composition to change of direction speed in police officers while carrying load. Int J Morphol. 2020 Jun;38(3):731-6. doi: 10.4067/S0717-95022020000300731.
38. Kukić F, Streetman A, Heinrich KM, Popović-Mančević M, Koropanovski N. Association between police officers' stress and perceived health. Polic - J Policy Pract. 2023 Jan 1;17:paad058. doi: 10.1093/police/paad058
39. Lan FY, Yiannakou I, Scheibler C, Hershey MS, Cabrera JL, Gaviola GC, Fernandez-Montero A, Christophi CA, Christiani DC, Sotos-Prieto M, Kales SN. The effects of fire academy training and probationary firefighter status on select basic health and fitness measurements. Med Sci Sports Exerc. 2021 Apr 1;53(4):740-748. doi: 10.1249/MSS.0000000000002533.
40. Lieberman HR, Thompson LA, Caruso CM, Niro PJ, Mahoney CR, McClung JP, Caron GR. The catecholamine neurotransmitter precursor tyrosine increases anger during exposure to severe psychological stress. Psychopharmacology (Berl). 2015 Mar;232(5):943-51. doi: 10.1007/s00213-014-3727-7. Epub 2014 Sep 16.
41. Lin KH, Su FY, Yang SN, Liu MW, Kao CC, Nagamine M, Lin GM. Body mass index and association of psychological stress with exercise performance in military members: The cardiorespiratory fitness and hospitalization events in armed forces (CHIEF) study. Endocr Metab Immune Disord Drug Targets. 2021;21(12):2213-2219. doi: 10.2174/1871530321666210427090550.
42. McAllister MJ, Martaindale MH, Rentería LI. Active shooter training drill increases blood and salivary markers of stress. Int J Environ Res Public Health. 2020 Jul 13;17(14):5042. doi: 10.3390/ijerph17145042.

43. McAllister MJ, Martaindale MH. Women demonstrate lower markers of stress and oxidative stress during active shooter training drill. Compr Psychoneuroendocrinol. 2021 Mar 8;6:100046. doi: 10.1016/j.cpnec.2021.100046.
44. McClung JP, Karl JP, Cable SJ, Williams KW, Young AJ, Lieberman HR. Longitudinal decrements in iron status during military training in female soldiers. Br J Nutr. 2009 Aug;102(4):605-9. doi: 10.1017/S0007114509220873.
45. Morse JL, Wooldridge JS, Herbert MS, Afari N. The impact of COVID-19 on health behavior engagement and psychological and physical health among active-duty military enrolled in a weight management intervention: An exploratory study. Mil Med. 2024 Aug 30;189(9-10):e1840-e1845. doi: 10.1093/milmed/usae092.
46. Ojanen T, Häkkinen K, Hanhikoski J, Kyröläinen H. Effects of task-specific and strength training on simulated military task performance in soldiers. Int J Environ Res Public Health. 2020 Oct 30;17(21):8000. doi: 10.3390/ijerph17218000.
47. Penatzer JA, Miller JV, Han AA, Prince N, Boyd JW. Salivary cytokines as a biomarker of social stress in a mock rescue mission. Brain Behav Immun Health. 2020 Mar 28;4:100068. doi: 10.1016/j.bbih.2020.100068.
48. Pineda Calderón CF, Monteros Luzuriaga GS, Yepez Herrera ER, Guerron Varela ER. Physical conditioning and its relationship with stress in the National Police of the Metropolitan District of Quito. Retos. 2023 Mar;48:505-10.
49. Proessl F, Canino MC, Beckner ME, Conkright WR, LaGoy AD, Sinnott AM, Eagle SR, Martin BJ, Sterczala AJ, Roma PG, Dretsch MN. Use-dependent corticospinal excitability is associated with resilience and physical performance during simulated military operational stress. J Appl Physiol (1985). 2022 Jan 1;132(1):187-198. doi: 10.1152/japplphysiol.00628.2021. Epub 2021 Dec 2.
50. Ramey SL, Downing NR, Franke WD, Perkhounkova Y, Alasagheirin MH. Relationships among stress measures, risk factors, and inflammatory biomarkers in law enforcement officers. Biol Res Nurs. 2012 Jan;14(1):16-26. doi: 10.1177/1099800410396356. Epub 2011 Feb 28.
51. Rodas KA, Dulla JM, Moreno MR, Bloodgood AM, Thompson MB, Orr RM, Dawes JJ, Lockie RG. The effects of traditional versus ability-based physical training on the health and fitness of custody assistant recruits. Int J Exerc Sci. 2022 Dec 1;15(3):1641-1660. doi: 10.70252/KPRM9974.
52. Rosalky DS, Hostler D, Webb HE. Work duration does not affect cortisol output in experienced firefighters performing live burn drills. Appl Ergon. 2017 Jan;58:583-591. doi: 10.1016/j.apergo.2016.04.008. Epub 2016 May 1.
53. Schilling R, Colledge F, Ludyga S, Pühse U, Brand S, Gerber M. Does cardiorespiratory fitness moderate the association between occupational stress, cardiovascular risk, and mental health in police officers? Int J Environ Res Public Health. 2019 Jul 3;16(13):2349. doi: 10.3390/ijerph16132349.
54. Schilling R, Herrmann C, Ludyga S, Colledge F, Brand S, Pühse U, Gerber M. Does cardiorespiratory fitness buffer stress reactivity and stress recovery in police officers? A real-life study. Front Psychiatry. 2020 Jun 23;11:594. doi: 10.3389/fpsyt.2020.00594.
55. Schilling R, Colledge F, Pühse U, Gerber M. Stress-buffering effects of physical activity and cardiorespiratory fitness on metabolic syndrome: A prospective study in police officers. PLoS One. 2020 Jul 28;15(7):e0236526. doi: 10.1371/journal.pone.0236526.
56. Scott JM, Kazman JB, Palmer J, McClung JP, Gaffney-Stomberg E, Gasier HG. Effects of vitamin D supplementation on salivary immune responses during Marine Corps basic training. Scand J Med Sci Sports. 2019 Sep;29(9):1322-1330. doi: 10.1111/sms.13467. Epub 2019 Jun 3.
57. Tait JL, Drain JR, Corrigan SL, Drake JM, Main LC. Impact of military training stress on hormone response and recovery. PLoS One. 2022 Mar 10;17(3):e0265121. doi: 10.1371/journal.pone.0265121.

58. Taylor MK, Stone M, Laurent HK, Rauh MJ, Granger DA. Neuroprotective–neurotrophic effect of endogenous dehydroepiandrosterone sulfate during intense stress exposure. Steroids. 2014 Sep;87:54-8. doi: 10.1016/j.steroids.2014.05.011. Epub 2014 Jun 2.
59. Tegeler CL, Gerdes L, Shaltout HA, Cook JF, Simpson SL, Lee SW, Tegeler CH. Successful use of closed-loop allostatic neurotechnology for post-traumatic stress symptoms in military personnel: self-reported and autonomic improvements. Mil Med Res. 2017 Dec 22;4(1):38. doi: 10.1186/s40779-017-0147-0.
60. Tingestad HC, Filion LG, Martin J, Spivock M, Tang V, Haman F. Stress and immune mediators in the Canadian Armed Forces: Association between basal levels and military physical performance. J Mil Veterans Health. 2019 Oct 1;27(4):15-23.
61. Toczko M, Fyock-Martin M, McCrory S, Martin J. Effects of fitness on self-reported physical and mental quality of life in professional firefighters: An exploratory study. Work. 2023;76(4):1589-1596. doi: 10.3233/WOR-220673.
62. Vicente-Rodríguez M, Fuentes-Garcia JP, Clemente-Suárez VJ. Psychophysiological stress response in an underwater evacuation training. Int J Environ Res Public Health. 2020 Mar 30;17(7):2307. doi: 10.3390/ijerph17072307.
63. Visconti LM, Palombo LJ, Givens AC, Turcotte LP, Kelly KR. Stress Response to Winter Warfare Training: Potential Impact of Location. Mil Med. 2024 Aug 19;189(Suppl 3):196-204. doi: 10.1093/milmed/usae075.
64. Watkins ER, Hayes M, Watt P, Richardson AJ. Heat tolerance of fire service instructors. J Therm Biol. 2019 May;82:1-9. doi: 10.1016/j.jtherbio.2019.03.005. Epub 2019 Mar 16.
