# Supplementary material for: Impact of Stress on Adrenal and Neuroendocrine Responses, Body Composition, and Physical Performance Amongst Women in Demanding Tactical Occupations: A Scoping Review
Source: Metabolites. 2025 Jul 29;15(8):506. doi: 10.3390/metabo15080506 (PMC12388157; doi:10.3390/metabo15080506)
Supplement: Supplementary file 1 [file metabolites-15-00506-s001.zip › TABLE-S3_Reporting-Qual-Summary-Table.pdf]

TABLE S3. Reporting completeness for individual checklist items (expressed as a percentage of items completely reported) for the total sample of included studies and each outcome of interest.

STROBE Items		All Studies	Adrenal and Neuroendocrine	Body Composition	Performance
TITLE & ABSTRACT					
	1a	10.1%	0.0%	13.8%	15.0%
	1b	89.9%	88.9%	93.1%	90.0%
INTRODUCTION					
Background/rationale	2	97.1%	100.0%	96.6%	100.0%
Objectives	3	44.9%	50.0%	41.4%	50.0%
METHODS					
Study design	4	89.9%	83.3%	93.1%	100.0%
Setting	5	81.2%	77.8%	86.2%	85.0%
Participants	6	44.9%	50.0%	41.4%	45.0%
Variables	7	95.7%	94.4%	96.6%	100.0%
Data sources/measurement	8	97.1%	94.4%	100.0%	100.0%
Bias	9	43.5%	55.6%	41.4%	40.0%
Study size	10	18.8%	11.1%	20.7%	25.0%
Quantitative variables	11	94.2%	94.4%	93.1%	100.0%
Statistical methods	12a	95.7%	94.4%	96.6%	100.0%
	12b	85.5%	77.8%	86.2%	95.0%
	12c	37.7%	50.0%	34.5%	35.0%
	12d	27.5%	33.3%	24.1%	30.0%
	12e	7.2%	11.1%	6.9%	5.0%
RESULTS					
Participants	13a	39.1%	50.0%	37.9%	30.0%
	13b	20.3%	22.2%	24.1%	15.0%
	13c	5.8%	11.1%	3.4%	5.0%
Descriptive data	14a	95.7%	100.0%	96.6%	95.0%
	14b	26.1%	33.3%	27.6%	20.0%
	14c	73.9%	72.2%	75.9%	80.0%
Outcome data	15	97.1%	100.0%	96.6%	100.0%
Main results	16a	95.7%	94.4%	96.6%	100.0%
	16b	5.8%	16.7%	3.4%	0.0%
	16c	1.4%	0.0%	3.4%	0.0%
Other analyses	17	91.3%	88.9%	89.7%	100.0%
DISCUSSION					
Key results	18	97.1%	100.0%	96.6%	100.0%
Limitations	19	76.8%	66.7%	82.8%	85.0%
Interpretation	20	98.6%	100.0%	100.0%	100.0%
Generalisability	21	79.7%	66.7%	82.8%	95.0%
OTHER INFORMATION					
Funding	22	69.6%	66.7%	69.0%	75.0%
**Overall Reporting Score**		**64.3% ± 11.3%**	**63.0% ± 13.1%**	**63.4% ± 11.9%**	**65.0% ± 8.3%**
